# Mercaptan-Mediated Ethylene Formation in Sulfur Oxidative Ethane Dehydrogenation on Iron Sulfide (FeS_2_) Catalysts

**DOI:** 10.1021/acscatal.5c07402

**Published:** 2026-03-20

**Authors:** Anik Biswas, Tobin J. Marks, Jeffrey Greeley

**Affiliations:** 1 Davidson School of Chemical Engineering, 311308 Purdue University, West Lafayette, Indiana 47906, United States; 2 Department of Chemistry, 3270 Northwestern University, Evanston, Illinois 60208, United States; 3 Department of Chemical and Biological Engineering, University of Wisconsin-Madison, Madison, Wisconsin 53706, United States

**Keywords:** soft oxidative dehydrogenation, iron sulfide, ethane, density functional theory, catalysis

## Abstract

Ethylene is a key feedstock for numerous industrial chemicals. It is conventionally produced via steam cracking, yet considerations of selectivity and downstream separation costs continue to motivate investigations of alternate production pathways. Catalytic ethane dehydrogenation (EDH) offers such an alternative, while the use of soft oxidants, such as sulfur, may alleviate the overoxidation that is characteristic of traditional oxidative approaches. This study explores the effectiveness of sulfur (S_2_) as an alternative oxidant for EDH. Using periodic density functional theory calculations, we explore the surface structures and sulfur coverages of FeS_2_ catalysts, which we have recently shown to be effective for this chemistry, under relevant temperature and pressure conditions. The results reveal that the catalyst experiences temperature-dependent surface sulfidation and that surface S-dimer species are catalytically active. The associated reaction energetics demonstrate that ethylene may be formed via both a mixed surface/gas phase mercaptan-mediated pathway and a conventional surface-catalyzed route on sulfur-covered FeS_2_ facets at intermediate temperatures. The preferred pathways and associated activation barriers are sensitive to the local structure of surface facets, with the sulfur vacancy formation energy and the average valence p-state electronic energy of surface sulfur atoms identified as plausible descriptors for the catalytic activity. The aggregate results shed light on the mechanistic interplay of sulfur and ethane on FeS_2_ surfaces and offer a potential pathway for developing efficient catalysts utilizing sulfur-based oxidants for selective ethylene production.

## Graphical abstract

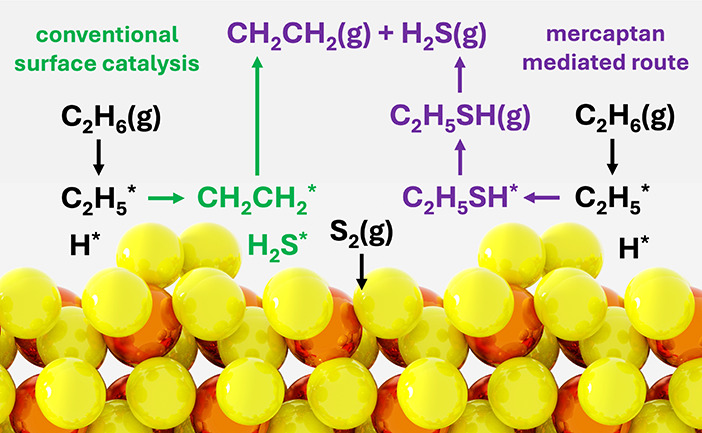

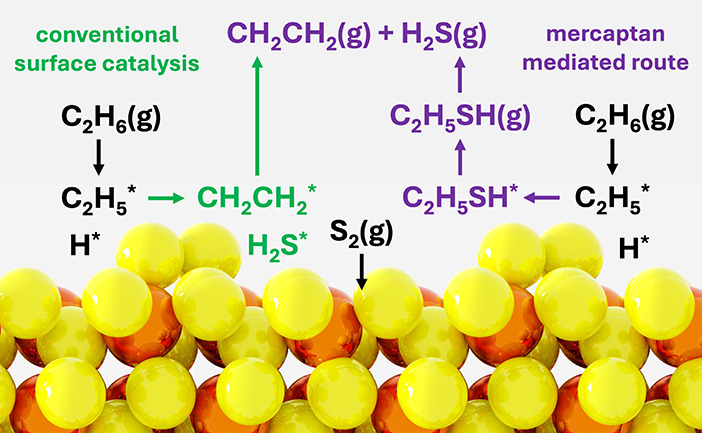

## 1 Introduction

Ethylene is a precursor to many industrially relevant chemicals such as polyethylene, ethylene oxide, ethylene glycol, vinyl chloride, and styrene.^1^ The global ethylene market size was over $150 billion in 2025 and is projected to expand at a compound annual growth rate of nearly six percent over the next decade.^2^ Ethylene is primarily produced via steam cracking of ethane and fluidized catalytic cracking of naphtha.^3^ However, concerns about the per-pass ethylene yield, coke formation, and energy-intensive downstream cryogenic separation continue to motivate explorations of alternative routes to ethylene production,^4^ including heterogeneous catalytic dehydrogenation of ethane. Nonoxidative ethane dehydrogenation (NO-EDH) on platinum-based alloy catalysts and chromium oxide catalysts has been extensively studied in the literature.^5^ However, the endothermic NO-EDH reaction is impeded by low single-pass conversion due to equilibrium limitations and by rapid deactivation due to coking and sintering.^6^ In contrast, oxidative ethane dehydrogenation (OEDH) can in theory achieve higher conversion than either steam cracking or NO-EDH.^7,8^ Gas phase oxygen (O_2_) is a typical oxidant, which makes the reaction exothermic and reduces external heat requirements for the reactor. In addition to ethylene, water is a coproduct of OEDH, and carbon deposits are readily removed from the catalyst sites as CO and CO_2_.^9^ However, there is a strong thermodynamic driving force for the overoxidation of ethylene to CO_2_, which reduces the selectivity for ethylene production. OEDH has predominantly been studied on supported metal oxide catalysts such as vanadium oxide, molybdenum oxide, and other readily reducible oxides that can exhibit multiple oxidation states during the redox cycle of the reaction.^10^ Detailed mechanistic analyses of OEDH over supported vanadium oxide catalysts, for example, have revealed that it follows a Mars-van Krevelen (MvK) pathway in which the surface lattice oxygen atoms participate in the reaction.^11–13^

As OEDH is still limited by suboptimal ethylene selectivity in the presence of strong oxidants, it is of interest to explore alternative oxidants for this chemistry.^14^ Carbon dioxide is the most widely investigated such alternative. CO_2_ can remove reaction byproduct H_2_ gas from the reaction environment through the reverse water–gas shift reaction, resulting in increased ethane conversion. However, while CO_2_-driven OEDH has demonstrated higher selectivity in some cases compared to purely O_2_-driven OEDH, it remains limited by equilibrium due to the endothermicity of the overall reaction, and coke may still form. N_2_O has also been assessed as an alternative oxidant for OEDH, but it is associated with similar challenges as CO_2_.^15^ Further, the presence of many elementary reaction steps involving CO_2_ or N_2_O leads to a highly intricate reaction network, making it challenging to develop fundamental mechanistic understanding.^16^

Another important class of alternative oxidants is that of sulfur-containing gases. Previously, it was reported that S_2_ vapor is relatively selective for partial alkane oxidation reactions such as methane coupling,^17–19^ ethane dehydrogenation,^20^ and propane dehydrogenation.^21–23^ O_2_ and S_2_ have similar valence shell electronic configurations, and thus in the presence of S_2_, OEDH is presumed to follow an analogous reaction pathway to ethylene and byproduct H_2_S. While S_2_–OEDH still remains exergonic, the thermodynamic driving force for overoxidation of ethylene to undesired CS_2_ is reduced significantly compared to overoxidation of ethylene to CO_2_ in the case of O_2_–OEDH.^20^ Some preliminary mechanistic insights have been obtained for these chemistries. For example, it has been reported that the initial C–H bond activation barrier for methane and propane is correlated with the metal–sulfur bond energy,^17,21^ while a MvK mechanism has been proposed for both S_2_-driven ethane and propane dehydrogenation reactions.^20,21^

This contribution focuses on the S_2_–OEDH reaction. Although many catalysts are plausible candidates for this chemistry, members of our team previously reported a maximum ethylene yield of 75.9% at 940 °C, and a maximum ethylene selectivity of 90.2% at 820 °C, over a sulfurized Fe_3_O_4_ catalyst.^20^ Detailed *ex situ* characterizations revealed that Fe_3_O_4_ completely converts to sulfide phases upon sulfurization, containing primarily FeS as the bulk phase with FeS_2_ nanocrystallites in the surface region.^20,19^ DFT calculations showed that the activation barrier for methane is notably lower on FeS_2_ surfaces than on FeS surfaces.^19^ As the initial C–H bond activation is usually the rate-determining step for alkane activation, therefore, the FeS surfaces are proposed to be catalytically irrelevant compared to the FeS_2_ surfaces. As the activation of ethane is chemically akin to the activation of methane on a catalyst surface, we further propose that the ethane dehydrogenation rate would be higher on FeS_2_ surfaces than on FeS. These considerations, in addition to the general observation that transition metal sulfide catalysts have been widely used for hydrotreating,^24,25^ motivate our computational exploration of the surface structures of FeS_2_ catalysts under the reaction conditions of S_2_–OEDH.

Previous computational studies have demonstrated that the (100) facet of FeS_2_ has the lowest surface energy among the (100), (110), (111), and (210) surface facets under vacuum conditions.^26,27^ However, when sulfur is present, the surface structures can be sensitive to the reaction temperature and partial pressure of gas phase S_2_. Alfonso addressed some aspects of these structural transformations using^28^ a grand canonical approach to estimate the surface free energies of multiple terminations of similar surface facets of FeS_2_ over a wide range of temperature and S_2_ partial pressures. Our analysis extends these results to consider a wide range of sulfur adspecies and defects. In general, the analysis indicates that increasing temperature reduces the extent of surface sulfidation, whereas decreasing temperature promotes sulfur clustering and chain formation. This dynamic evolution of surface sulfur coverage and structure plays a central role in determining catalytic behavior under S_2_–OEDH conditions.

While a MvK-type mechanism has been proposed for this reaction,^20^ the detailed reaction steps are a subject of continuing debate. To shed light on these questions, in this work, we investigate the energetics of a simple reaction scheme (Figure 1) for S_2_–OEDH, considering several possible pathways. This scheme begins with the physisorption of gas phase ethane, followed by its activation and dissociative adsorption to form C_2_H_5_ and H. Subsequently, we explore both selective C–H bond dissociation to form the desired product, CH_2_CH_2_ (ethylene), and nonselective C–H bond dissociation to form CH_3_CH. The resulting CH_2_CH_2_ can either desorb to form gas phase ethylene or undergo further dehydrogenation. Given that S_2_–OEDH reactions typically occur at elevated temperatures (700–900 °C), we additionally evaluate the likelihood of C_2_H_5_ radical desorption and its implications for gas phase chemistry. This mechanism has some analogies with radical-mediated pathways in industrial steam cracking, where C_2_H_5_ radicals drive CH_2_CH_2_ formation as a primary product.^29^ Under S_2_–OEDH conditions, desorbed surface-generated C_2_H_5_ radicals could similarly participate in gas phase reactions, enabling CH_2_CH_2_ production through β-scission or radical recombination/disproportionation. This mechanism thus explores a potential synergy between surface-mediated activation and gas phase radical chemistry, which could become increasingly important at elevated temperatures.

**Figure 1:**
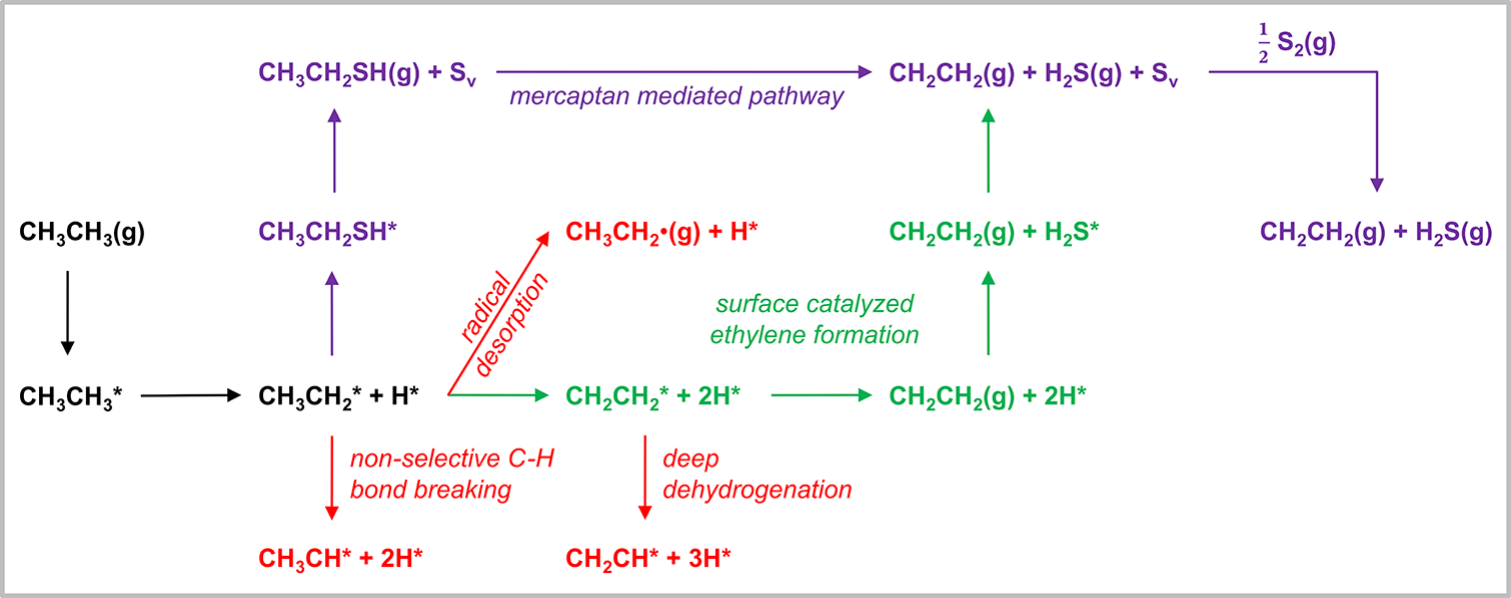
Considered reaction network for ethane (CH_3_CH_3_) conversion to ethylene (CH_2_CH_2_). The green pathway depicts a selective surface-catalyzed dehydrogenation pathway. The elementary steps marked in red denote primary nonselective C–H bond dissociation pathways and the pathway leading to gas phase radical chemistry. The purple reaction steps show a mercaptan-mediated pathway. S_v_ denotes a S-vacancy on the surface.

An additional scenario in which surface and gaseous reaction pathways could be linked involves direct insertion of S-species into hydrocarbon backbones. In OEDH with O_2_ as a reactant, oxygen insertion into the C_2_ molecule is challenging. Strong nonselective oxidation reactions typically dominate, resulting in minimal yields of partially oxygenated products and predominantly forming fully oxidized CO_2_. In contrast, substituting O_2_ with S_2_ in the analogous S_2_–OEDH process suppresses overoxidation and enables stable sulfur incorporation into surface intermediates, motivating exploration of a mercaptan-mediated pathway in which ethyl mercaptan (C_2_H_5_SH) forms on the catalyst surface and desorbs (Figure 1). Subsequent gas phase thermal decomposition of C_2_H_5_SH selectively yields CH_2_CH_2_ and H_2_S.^30–32^ Computational studies indicate that this decomposition can proceed either through unimolecular elimination of H_2_S or via cleavage of the weaker C–S bond, generating gaseous C_2_H_5_ and SH radicals, followed by rapid β-scission of C_2_H_5_ to form CH_2_CH_2_.^33^

The present study combines a detailed investigation of the temperature- and pressure-dependent structure of FeS_2_ surfaces with consideration of the various mechanistic possibilities discussed above. The S_2_–OEDH reaction mechanism is investigated on both S-dimer-covered and pristine FeS_2_ surfaces. On the S-dimer-covered (001)-S and (210)-2S′ facets, ethylene formation at approximately 1000 K proceeds via two competitive pathways: a mercaptan-mediated mechanism and a conventional surface-catalyzed route (Figure 1). For comparison, pristine (001)-S, (210)-2S′, and (111)-3S surfaces (without additional sulfur adsorption) are also examined. On pristine (001)-S at 1000 K, desorption of the C_2_H_5_ radical competes with both surface catalyzed and mercaptan-mediated pathways, while nonselective C–H bond dissociation leading to CH_3_CH is kinetically unfavorable. By comparison, on pristine (210)-2S′ under similar conditions, C_2_H_5_ radical desorption and mercaptan-mediated pathways are both energetically unfavorable. Selective surface-catalyzed CH_2_CH_2_ formation competes with nonselective C–H bond dissociation that generates CH_3_CH. In contrast, the (111)-3S surface favors direct surface-catalyzed ethylene production. These findings reveal a nuanced interplay between surface structures and reaction pathway preference, with the mercaptan-mediated mechanism emerging as a contributor to ethylene formation on sulfur-rich surfaces. The analysis further shows that the activation barrier for the rate limiting step of S_2_–OEDH, the initial C_2_H_6_ activation, is linearly correlated with the average valence p-orbital energy and the vacancy formation energy of surface sulfur sites on FeS_2_. This correlation suggests that these quantities can serve as effective activity descriptors for S_2_–OEDH on sulfided catalysts.

## 2 Methods

### 2.1 DFT Relaxations

Spin-polarized, periodic, planewave density functional theory (DFT) calculations are performed with the Vienna Ab-initio Simulation Package (VASP).^34^ The exchange-correlation functional developed by Perdew, Burke, and Ernzerhof (PBE)^35^ is employed with the Projector Augmented Wave (PAW) core electron treatment.^36^ The energy cutoff for the planewave basis set for all calculations is 400 eV. Long range electronic interactions are captured by implementing D3 dispersion correction devised by Grimme et al.^37,38^ The energy and force convergence conditions for all relaxation calculations are set at 10^–4^ eV and 0.02 eV/Å, respectively.

### 2.2 Bulk Structure

The bulk FeS_2_ (pyrite) cubic structure, with space group *Pa*3̅, is obtained from the Materials Project database (Figure S1).^39^ The Brillouin zone for the bulk structure is sampled by a Monkhorst–Pack *k*-point mesh of 8 × 8 × 8.^40^ The DFT-relaxed bulk structure has a lattice constant of 5.38 Å, in good agreement with the experimental value of 5.42 Å.^41^

The phase diagram for the iron–sulfur system indicates that, at approximately 900 K and an S_2_ fugacity of 0.01 atm , FeS_2_ decomposes into pyrrhotite (FeS_*x*_, 1 < *x* < 2) and sulfur gas.^42^ Although this is somewhat lower than the reaction temperatures considered in the present study, it is important to note that phase diagrams typically represent equilibrium conditions in bulk materials. In contrast, our study focuses on surface phenomena under dynamic reaction conditions, and experimental characterizations have, indeed, demonstrated that FeS_2_ can persist as nanocrystallites in the surface phase, exhibiting enhanced thermal stability compared to its bulk counterpart.^19^ This nanoscale morphology, coupled with the unique properties of surface phases and the presence of reactive gas environments, can significantly enhance the stability landscape of FeS_2_.

### 2.3 Surface Structures

A graph theory-based code, CatKit,^43^ is used to cleave the DFT-relaxed bulk structure in various orientations, corresponding to (100), (110), (111), and (210) Miller indices. Each surface Miller index can exhibit both stoichiometric and nonstoichiometric terminations, as described in a previous study.^28^ A grand canonical surface free energy formalism,^28,44,45^ described in more detail in the Supporting Information, is generated by assuming that the surface is in equilibrium with the underlying bulk sulfide and that the gas phase sulfur is in chemical equilibrium with the surface sulfur species. The resulting grand canonical surface free energy diagram (Supporting Information) is consistent with, and builds upon, previously reported analysis by Alfonso.^28^ The gas phase chemical potential of S_2_ is a function of temperature and pressure. In this work, an S_2_ partial pressure of 0.01 atm is employed to analyze the surface phase stability and reaction energy landscape, consistent with previously reported experimental reaction conditions.^20^ The surface free energy diagram (Figure S4a) at a gas phase S_2_ partial pressure of 0.01 atm indicates that, among the bulk-terminated surface structures, the (001)-S facet (Figure 2a,c) is the most thermodynamically stable at the operating temperature range for S_2_–OEDH.^28^ The (210)-2S′ facet, in turn, exhibits the second lowest surface energy (Figure 2b,d). The entropy change of the surface atoms during cleavage from the bulk is neglected, as the relative difference in vibrational entropy among different surface terminations is marginal and does not significantly affect the relative thermodynamic stability of the low energy surfaces.^28^

**Figure 2:**
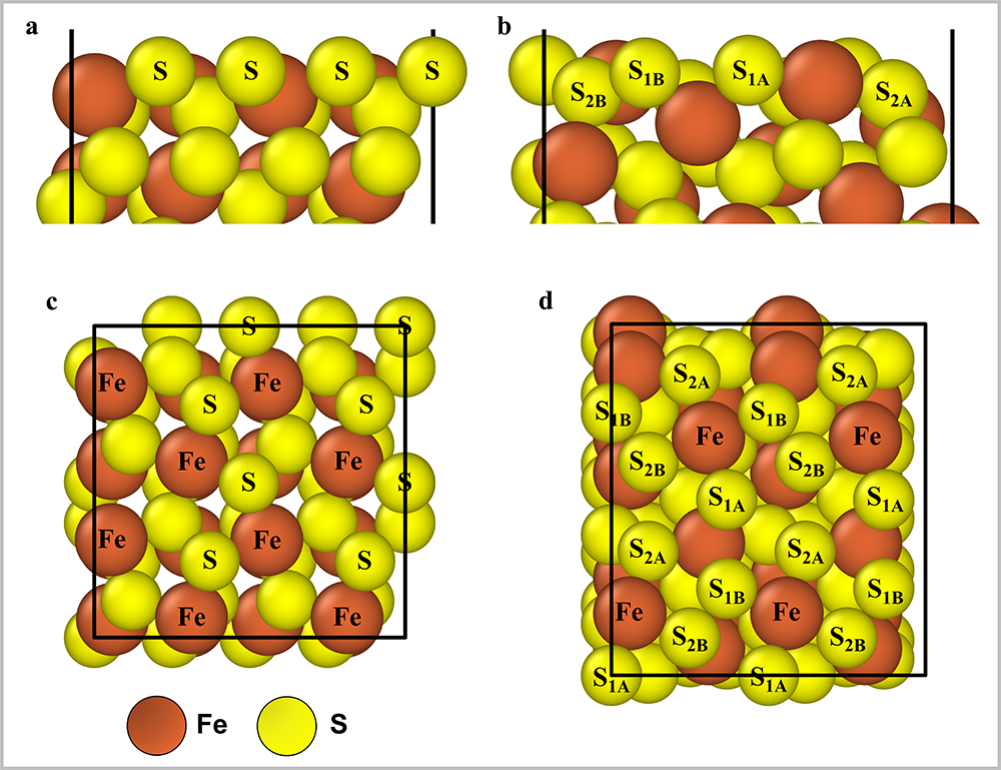
Side views of surfaces (001)-S and (210)-2S′ are shown in (a) and (b), respectively. Top views of surfaces (001)-S and (210)-2S′ are shown in (c) and (d), respectively. The unique surface sites are marked on each diagram.

To model the (001)-S facet, a 2 × 2 × 3 surface slab is employed, and the Brillouin zone is sampled using a 4 × 4 × 1 *k*-point mesh. For the (210)-2S′ surface slab, a 2 × 2 × 5 supercell is selected with a 4 × 4 × 1 *k*-point mesh. Finally, a 1 × 1 × 2 surface slab with a 6 × 6 × 1 *k*-point mesh is used for the (111)-3S facet (Figure S3), representing a model sulfur-rich surface for studying reaction energetics. The (111)-3S surface is among the lowest surface energy structures near the reaction operating temperature (Figure S4a), becoming the most stable surface at temperatures lower than 750 K. The dimensions of the supercell (x, y, and z) are defined based on the positioning of the Fe atoms.

### 2.4 Additional Surface Sulfur Structures

The pristine, bulk-terminated structures described in Section 2.2 are complemented by analysis of distinct arrangements with either excesses or deficiencies of S atoms. For the (001)-S, (210)-2S′, and (111)-3S facets, sulfur atoms are removed to create sulfur-defect surface structures. Similarly, sulfur adatoms, dimers, and other oligomeric structures, including trimers and tetramers, are also analyzed on (001)-S and (210)-2S′ facets. A detailed description of these structures is provided in Section 3.1 and in the Supporting Information, and their surface free energies are determined using the same grand canonical approach as described above.

### 2.5 Surface Sites and Binding Configurations

The (001)-S surface contains two types of sites, dubbed Fe and S (Figure 2a,c). The (210)-2S′ surface is terminated by two different layers of sulfur atoms (Figure 2b). Each sulfur layer, in turn, contains two different types of sulfur atoms. Thus, the (210)-2S′ facet exhibits five unique surface sites (Figure 2d), including the Fe site, S_1A_/S_1B_ sites (from the top S-layer termination), and S_2A_/S_2B_ sites (from the second S-layer termination). On the 111-(3S) surface, Fe sites are not accessible to the adsorbates. The surface therefore has three unique sites (Figure S3), denoted S_1_, S_2_, and S_3_, depending upon the terminating sulfur layer to which they belong.

For all sulfur adstructures, all possible geometries permitted by the unit cell symmetries are explicitly analyzed. Similarly, for monodentate or bidentate C_2_H_*x*_ species, adsorption at all unique surface sites is evaluated, and for coadsorbate configurations corresponding to the final geometries of S–S and C–H bond dissociation, all pairs of neighboring sites are examined.

### 2.6 Transition State Search

The Climbing Image Nudged Elastic Band (CINEB) method^46^ is implemented to find the transition states, as well as the associated activation barriers, between local minima involving elementary bond breaking pathways or diffusion steps. Thereafter, the dimer method^47^ is used to refine the transition state energies. Finally, a vibrational frequency calculation is performed on the transition state structures to verify that a single imaginary frequency exists.

### 2.7 Vibrational Frequency Calculations and Entropy Estimation

To reflect the enhanced accuracy needed to compute second derivatives of energies, an energy convergence criterion of 10^–6^ eV is employed for vibrational frequency calculations. Mode decomposition analysis is then performed using the obtained vibrational frequencies. As noted by Campbell and Sellers,^48^ the harmonic approximation of all vibrational modes significantly underestimates the entropy of surface species at high temperatures. Hence, only vibrational modes with frequencies greater than approximately 150 cm^–1^ are treated as harmonic oscillators. Modes below this cutoff are treated as hindered translators, hindered rotors,^49^ one or two-dimensional particles in a box, or one or two-dimensional ideal gases. For the transition states of C–H bond dissociation steps, the entropy contribution from the imaginary frequency is excluded. Translational and rotational entropies of the transition states are approximated using the entropies of either the initial or final states, depending on which is geometrically closer to the transition state. The barriers associated with the hindered translations and rotations are, in turn, determined using the CINEB method. For hindered translation, the effective diffusion barrier is determined as the maximum energy required for a species to move between two identical sites.

When constructing free energy diagrams, entropic corrections are applied to account for the change in entropy contributions of the surface S-dimer units (or S-adatoms, in cases where the S–S bond is dissociated) in the presence of adsorbates. This approach ensures that the thermodynamic profiles accurately reflect the dynamic nature of the surface under reaction conditions, capturing the influence of adsorbate-induced changes in surface entropy. For S–S bond dissociation and H-diffusion steps, the transition state entropy is estimated as the average of the corresponding initial and final states. The transition state for surface species desorption is located on a two-dimensional plane parallel to the surface, slightly elevated above it.^48^ Therefore, the translational entropy of the desorption process is modeled using a two-dimensional particle-in-a-box approach. Finally, configurational entropy is also included in the estimation of surface free energy for various sulfur-decorated structures. The partition functions for the different modes of vibration are detailed in the Supporting Information.

### 2.8 Average Valence p-State Energy Calculation

To evaluate electronic densities of states, dense k-point meshes are employed: 13 × 13 × 1 for (001)-S, 13 × 11 × 1 for (210)-2S′, and 15 × 15 × 1 for (111)-3S. Using the generated electronic structures, projected density of states (PDOS) calculations are performed. The average p-state energy of surface atoms in the slab is then extracted from the PDOS results using the VASPKIT software package.^50^

## 3 Results

### 3.1 Grand Canonical Free Energies of Sulfur-Decorated Surface Structures

The surface free energy of (001), (110), (111), and (210) surface terminations is plotted against temperature in Figure S4a at a sulfur (S_2_) partial pressure of 0.01 atm. Each line in the plot represents a unique bulk-terminated surface of FeS_2_; these surfaces are cleaved directly from the bulk but are not necessarily stoichiometric. Above 750 K, the (001)-S surface exhibits the lowest surface energy, while below 750 K, the (111)-3S surface is preferred. Near 750 K, the (001)-S, (210)-2S′, and (111)-3S surfaces have similar surface energies. As (001)-S is a stoichiometric surface termination, its surface energy remains independent of the temperature. However, the surface energies of sulfur-rich, nonstoichiometric (210)-2S′ and (111)-3S surface terminations decrease linearly at lower temperatures.

It is well established that the coordination environment of surface sites significantly influences catalyst reactivity,^51,52^ and in the context of transition metal sulfide catalysts, engineering of undercoordinated sulfur sites has been shown to enhance catalytic performance for electrocatalytic and photocatalytic applications.^53–55^ Previous DFT studies have, further, indicated that sulfur dimers and adatoms can influence the reaction energetics for oxidative coupling of methane on FeS_2_ surfaces.^19^ Thus, beginning with the bulk-terminated surfaces, (001)-S and (210)-2S′, described above, we additionally construct, and analyze the thermodynamic stability of, sulfur defects, adatoms, dimers, and other polysulfide structures.

The analysis of additional sulfur surface structures begins with the formation of sulfur vacancies. Table S1 summarizes the sulfur vacancy formation energies for all unique surface sulfur atoms across the three facets. The S-vacancy formation energy on each facet inversely correlates with the degree of surface sulfidation. Across all facets, the formation of the first sulfur vacancy is endothermic. Therefore, further investigation into vacancy formation energies at higher sulfur vacancy coverages is not pursued, as such configurations are expected to be even less thermodynamically favorable.

In the S_2_–OEDH reaction, gas phase S_2_ can directly adsorb from the reactant stream onto catalyst surfaces, which can further undergo dissociation to form S-adatoms. Moreover, FeS_2_ surfaces can promote the formation of larger sulfur clusters, such as S-trimers and S-tetramers, through the coalescence of multiple S-adatoms, S-dimers, or combinations thereof. Following the analysis of sulfur vacancies, the energetics of surface S-adatoms, S-dimers, S-trimers, and S-tetramers are investigated on the (001)-S and (210)-2S′ surfaces. Binding configurations of S-adatoms at 1/8 monolayer (ML) coverage and S-dimers, S-trimers, and S-tetramers at 1/4 ML coverage on the (001)-S facet are shown in Figures S5–S8, respectively. Binding geometries of S-adatoms, S-dimers, S-trimers, and S-tetramers at 1/4 ML coverage on the (210)-2S′ facets are shown in Figures S9–S12, respectively. We further examine the formation energy of such S-decorations at a higher coverage of 1 ML. The most stable S-adatom, S-dimer, S-trimer, and S-tetramer structures at 1 ML coverage are reported in Figure 3b–e for the (001)-S surface and in Figure 4b–e for the (210)-2S′ surface, respectively. The most stable binding sites for S-dimer, S-trimer, and S-tetramer structures are Fe–Fe and Fe–S_1A_, respectively, for the (001)-S, and (210)-2S′ facets at both low and high coverages.

**Figure 3:**
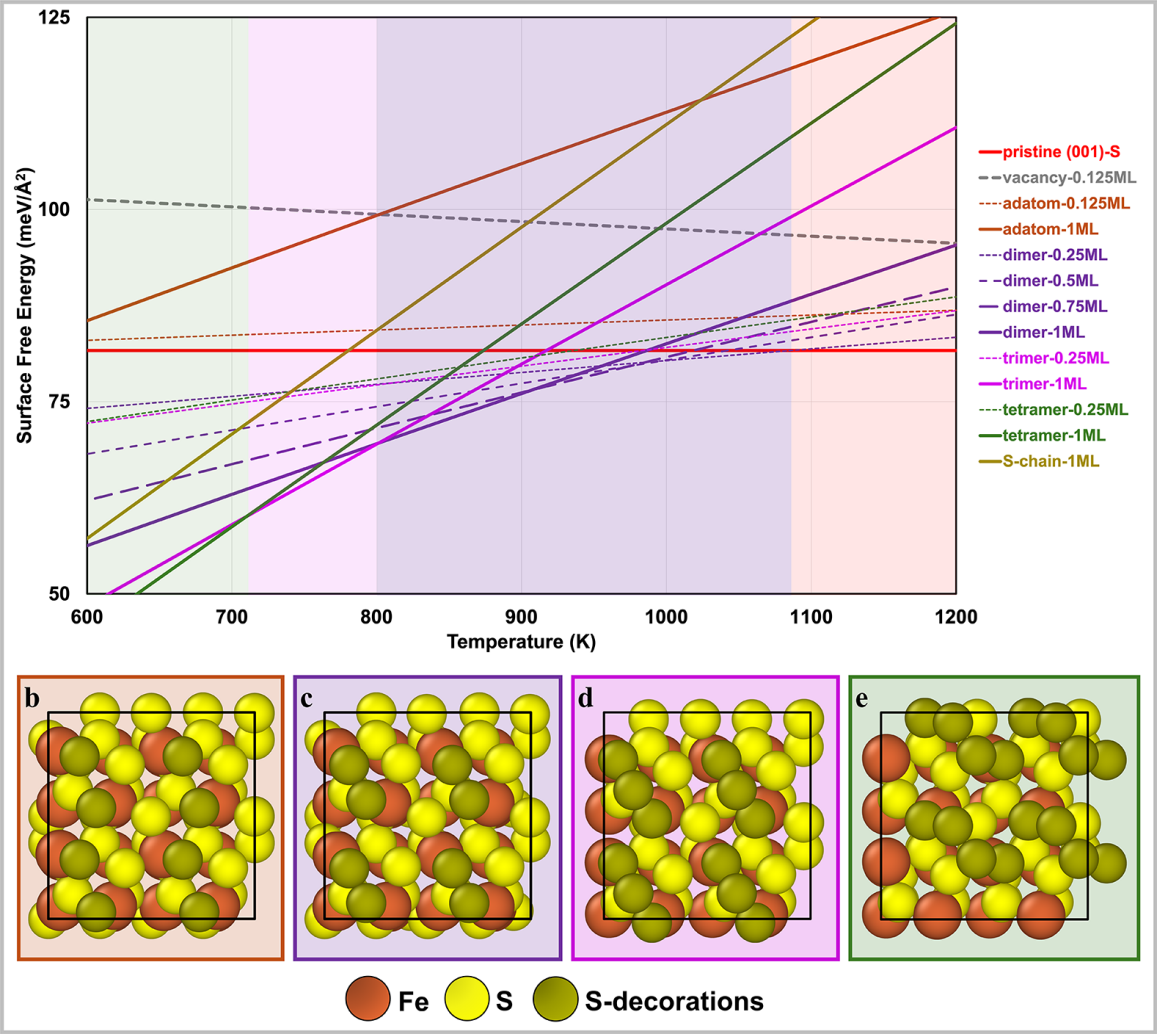
(a) Grand canonical surface free energy diagram of sulfur structures on the FeS_2_ (001)-S surface at a gas phase S_2_ partial pressure of 0.01 atm. Solid lines represent 1 ML coverage, while dashed lines indicate lower coverages. The pristine (001)-S surface is shown in red. Other structures are represented as follows: S-vacancy (gray), S-adatoms (orange), S-dimers (purple), S-trimers (magenta), S-tetramers (green), and S-chain (gold). The structures for (b) S-adatom, (c) S-dimer, (d) S-trimer, and (e) S-tetramer at 1 ML coverage are shown. The diagram illustrates the relative thermodynamic stability of these sulfur configurations across a range of temperatures, with color-matched shaded areas highlighting stability regions.

**Figure 4:**
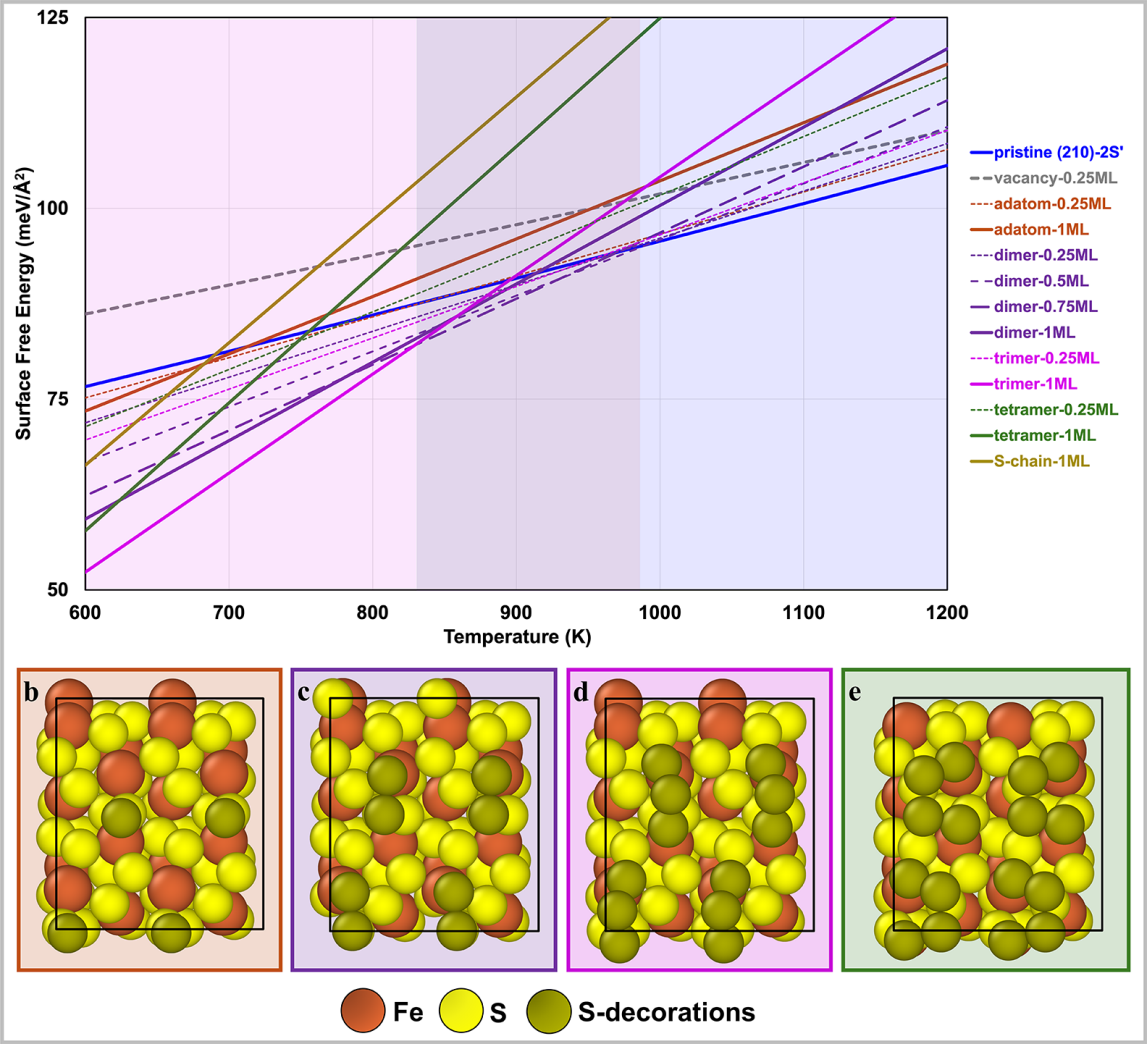
(a) Grand canonical surface free energy diagram of sulfur structures on the FeS_2_ (210)-2S′ surface at a gas phase S_2_ partial pressure of 0.01 atm. Solid lines represent 1 ML coverage, while dashed lines indicate lower coverages. The pristine (210)-2S′ surface is shown in blue. Other structures are represented as follows: S-vacancy (gray), S-adatoms (orange), S-dimers (purple), S-trimers (magenta), S-tetramers (green), and S-chain (gold). The structures for (b) S-adatom, (c) S-dimer, (d) S-trimer, and (e) S-tetramer at 1 ML coverage are shown. The diagram illustrates the relative thermodynamic stability of these sulfur configurations across a range of temperatures, with color-matched shaded areas highlighting stability regions.

The formation energies of the S-adatom, S-dimer, S-trimer, and S-tetramer structures at low and high coverages are listed in Tables S2–S5, respectively. We note that some of the S-decorated structures show adsorbate–adsorbate interactions depending on their spatial separations. At higher coverages, some S-tetramers on both (001)-S and (210)-2S′ surfaces coalesce to form continuous S-chains that extend across the periodic surface (Figure S13). These chains are therefore slightly less stable than the isolated tetramers described above (Table S5).

Based on the most stable configurations of S-vacancy, S-adatom, and clustered sulfur dimer, trimer, and tetramer structures, the surface free energies of the structures described above are calculated using the ab initio thermodynamics approach (see Supporting Information for details). The surface free energies at both low and full coverage of the respective (001)-S and (210)-2S′ surface slabs are considered.

#### 3.1.1 (001)-S Surface

Figure 3a illustrates the surface free energies of several low and high coverage sulfur structures as a function of temperature, with the 900–1000 K range being of particular relevance to S_2_–OEDH operating conditions at a gas phase S_2_ partial pressure of 0.01 atm. The pristine (001)-S surface (solid red line) exhibits a surface energy of 82 meV/Å^2^ which, due to the particular stoichiometry of the surface, does not change with temperature. Although the surface free energy of the 0.125 ML S-vacancy (solid gray line) decreases with increasing temperature, it remains higher than that of the pristine (001)-S surface throughout the temperature range of 600–1200 K due to the unfavorable formation energy of S-vacancies on this surface. Consequently, S-vacancies are unlikely to be prevalent on the (001)-S surface under typical S_2_–OEDH reaction conditions.

For 0.125 ML coverage of S-adatoms, the surface free energy (dashed orange line) increases with temperature, remaining marginally higher than that of the pristine (001)-S surface across the temperature range. At a full 1 ML coverage, the surface free energy (solid orange line) increases more rapidly with temperature and exceeds that of the 0.125 ML coverage throughout temperature range.

For the S-dimer structures, we investigate the surface free energy at coverages of 0.25, 0.5, 0.75, and 1 ML. As mentioned previously, S-dimers bind strongly to the Fe–Fe sites on the (001)-S surface, resulting in a lower surface energy than that of the pristine (001)-S surface. Consequently, they represent the thermodynamically most stable and relevant surface structures under S_2_–OEDH operating reaction conditions. The surface free energies for 0.25, 0.5, and 0.75 ML coverages are represented by dashed purple lines, while the surface free energy for the 1 ML coverage of S-dimers is represented by a solid purple line. Between 800 and 925 K, a full monolayer (1 ML) of S-dimers exhibits the lowest surface energy. However, above 925 K, the equilibrium S-dimer coverage gradually decreases with increasing temperature, eventually yielding a pristine (001)-S surface near 1100 K. Given that S-dimer structures are prevalent on the (001)-S surface above 800 K, we selected the 1 ML S-dimer-covered (001)-S surface, with the bulk-terminated surface included for comparison, for subsequent investigations of the reaction mechanism and energetics of S_2_–OEDH (Section 3.2 below).

The surface free energies of S-trimer and S-tetramer structures at a coverage of 0.25 ML are represented by dashed magenta and green lines, respectively. At 1 ML coverage, these S-structures exhibit enhanced thermodynamic stability compared to their low coverage counterparts at the lower end of the temperature range. The 1 ML coverage of S-trimers (solid magenta line) is the most thermodynamically stable between 700 and 800 K, whereas the 1 ML S-tetramer structures (solid green line) are dominant on the surface below 700 K.

Finally, to illustrate the effect of pressure on the surface thermodynamics, we construct a surface free energy diagram (Figure S14a) at a gas phase S_2_ partial pressure of 1 atm. The higher S_2_ gas phase pressure increases the chemical potential, promoting adsorption of S_2_ molecules onto the surface. Consequently, even at elevated temperatures (∼1200 K), the surface is likely to remain populated with S-dimer structures.

#### 3.1.2 (210)-2S′ Surface

Figure 4a illustrates the thermodynamic stability of various sulfur structures on the (210)-2S′ surface as a function of temperature at a gas phase S_2_ partial pressure of 0.01 atm. The pristine surface serves as a baseline reference for evaluating the stability of other sulfur-covered configurations. Unlike the (001)-S facet, the pristine (210)-2S′ surface is nonstoichiometric and exhibits increasing surface energy with increasing temperature (solid blue line). This behavior is attributed to the reduced stability of surface sulfur at elevated temperatures.

S-vacancy structures at low coverages (0.125 ML - dashed gray line) show a gradual increase in surface free energy with temperature, although the rate of increase is lower compared to the pristine surface. Despite this slower increase, the surface free energies of S-vacancy structures remain consistently higher than those of the pristine surface across the studied temperature range. This thermodynamic instability suggests that S-vacancies are unlikely to form or persist on the (210)-2S′ surface under typical S_2_–OEDH reaction conditions.

For S-adatoms, at low coverage (0.125 ML - dashed orange line), the surface free energy increases with temperature at a higher rate than that of the pristine surface, due to the presence of excess sulfur on the surface. At lower temperatures, the surface free energy of S-adatoms is marginally lower than that of the pristine surface, but it becomes slightly higher at elevated temperatures. At a coverage of 1 ML (solid orange line), the increase in surface free energy is even more pronounced, indicating that entropic destabilization outweighs enthalpic stabilization as coverage and temperature rise, as is observed on the (001)-S facet. These findings underscore the limited thermodynamic relevance of S-adatoms on the (210)-2S′ surface under typical reaction conditions.

S-dimer structures exhibit strong binding to Fe–S_1A_ sites on the (210)-2S′ facet, making them considerably more stable than both pristine and S-adatom configurations below 1000 K. Surface free energies for these dimers are calculated for coverages of 0.25, 0.5, 0.75, and 1 ML. Above 825 K, S-dimers dominate as the thermodynamically most favorable structures. However, as temperature increases beyond this threshold, the stability of S-dimer-covered surfaces diminishes, with coverage gradually decreasing from 1 ML to pristine (210)-2S′ at approximately 1000 K. These results indicate that S-dimer-covered surfaces are likely to play a role in catalytic performance under typical S_2_–OEDH operating conditions, particularly at temperatures between 825 and 1000 K. As with the (001)-S surface, we chose the 1 ML S-dimer-covered (210)-2S′ surface, as well as the pristine (210)-2S′ surface, for subsequent investigations of the reaction mechanism and energetics of S_2_–OEDH (Section 3.2 below).

In addition to S-dimers, higher-order sulfur structures, such as S-trimers and S-tetramers, are investigated. At a coverage of 0.25 ML, their surface free energies are represented by dashed magenta and green lines, respectively. At higher coverage (1 ML), both structures demonstrate enhanced thermodynamic stability at lower temperatures (<825 K), with S-trimers (solid magenta line) being the most stable species between 600 and 825 K. Linear S-chain structures are also evaluated at high coverages (solid gold line). However, unlike the sulfur dimer configurations, these larger sulfur structures are thermodynamically stable only at temperatures well below the operating conditions for S_2_–OEDH, making them irrelevant on the (210)-2S′ facet under typical reaction conditions. Figure S14b shows that, under an S_2_ partial pressure of 1 atm, S-trimers are the predominant surface species across most of the temperature range from 600 to 1200 K, as the higher chemical potential of S_2_ shifts the equilibrium toward these species.

### 3.2 Reaction Mechanism and Energetics

Prior experimental S_2_–OEDH studies were conducted over temperatures ranging from approximately 850 to 1200 K.^20^ Those results indicate that, near 1000 K, S_2_–OEDH demonstrates intermediate ethane conversion and ethylene selectivity. We therefore perform reaction free energy analyses at 1000 K and a gas phase S_2_ partial pressure of 0.01 atm. Motivated by our phase diagram results, the free energies are determined on the 1 ML S-dimer-covered (001)-S and (210)-2S′ surfaces, which are among the thermodynamically most stable surface terminations. For comparison, reaction energetics are later assessed on pristine (001)-S and (210)-2S′ surfaces. Finally, reaction energetics re examined on the (111)-3S surface to provide comparative insights into reaction behavior on an alternative sulfur-rich, bulk-terminated facet.

Basic reaction steps are illustrated in Figure 1, but for S-dimer-covered surfaces, the mercaptan-mediated pathway introduces additional steps, including S–S bond dissociation and atomic H diffusion, which are required for the formation of surface-bound C_2_H_5_SH (Figure 5). This expanded mechanism explores both S–S bond dissociation in the presence of a C_2_H_5_ adsorbate on the S-dimer site, with H on a neighboring S-dimer, as well as the coadsorption of C_2_H_5_ and H on the same S-dimer unit. Similar mechanisms are analyzed on the pristine (001)-S, (210)-2S′, and (111)-3S surfaces, but detailed results for those surfaces are reserved for the Supporting Information.

**Figure 5:**
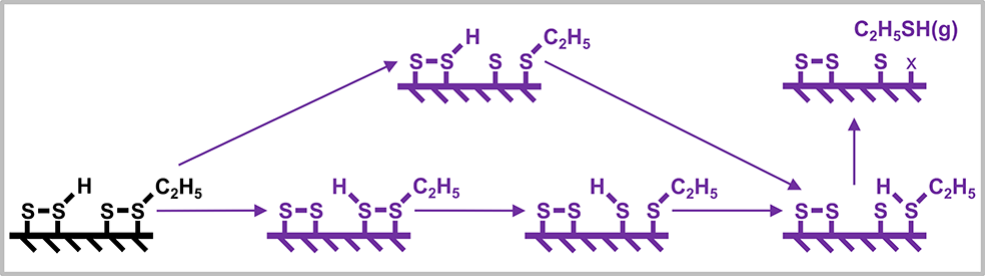
Reaction mechanism for ethyl mercaptan (C_2_H_5_SH) formation on S-dimer-covered (001)-S and (210)-2S′ surfaces. This mechanism requires S–S bond breaking of a surface S-dimer and atomic H diffusion.

For each surface, we begin by describing the binding sites and binding energies of reaction intermediates. The energies of adsorbed species, referenced to gas phase ethane and stoichiometrically appropriate amounts of gas phase hydrogen, are defined as

$$\Delta E_{C_{2}{H_{6}}^{*}}=\left[E_{\mathrm{slab}+C_{2}{H_{6}}^{*}}-E_{\mathrm{slab}}-E_{C_{2}H_{6}\left(g\right)}\right]$$ 1

$$\Delta E_{H^{*}}=[E_{\mathrm{slab}+H^{*}}-E_{\mathrm{slab}}-0.5E_{H_{2}(g)}]$$ 2

$$\Delta E_{CH_{3}C{H_{2}}^{*}}=\left[E_{\mathrm{slab}+CH_{3}C{H_{2}}^{*}}+0.5E_{H_{2}\left(g\right)}-E_{\mathrm{slab}}-E_{C_{2}H_{6}\left(g\right)}\right]$$ 3

$$\Delta E_{CH_{2}C{H_{2}}^{*}}=\left[E_{\mathrm{slab}+CH_{2}C{H_{2}}^{*}}+E_{H_{2}\left(g\right)}-E_{\mathrm{slab}}-E_{C_{2}H_{6}\left(g\right)}\right]$$ 4

$$\Delta E_{CH_{3}{\mathrm{CH}}^{*}}=\left[E_{\mathrm{slab}+CH_{3}{\mathrm{CH}}^{*}}+E_{H_{2}\left(g\right)}-E_{\mathrm{slab}}-E_{C_{2}H_{6}\left(g\right)}\right]$$ 5

$$\Delta E_{CH_{2}{\mathrm{CH}}^{*}}=\left[E_{\mathrm{slab}+CH_{2}{\mathrm{CH}}^{*}}+1.5E_{H_{2}\left(g\right)}-E_{\mathrm{slab}}-E_{C_{2}H_{6}\left(g\right)}\right]$$ 6

$$\Delta E_{C_{2}H_{5}{\mathrm{SH}}^{*}}=\left[E_{\mathrm{slab}+C_{2}H_{5}{\mathrm{SH}}^{*}}-E_{\mathrm{slab}}-E_{C_{2}H_{6}\left(g\right)}\right]$$ 7

$$\Delta E_{{CH}_{3}{{CH}_{2}}^{*}+H^{*}}=\left[E_{\mathrm{slab}+{CH}_{3}{{CH}_{2}}^{*}}+E_{\mathrm{slab}+H^{*}}-2E_{\mathrm{slab}}-E_{C_{2}H_{6}\left(g\right)}\right]$$ 8

$$\Delta E_{CH_{2}C{H_{2}}^{*}+2H^{*}}=\left[E_{\mathrm{slab}+CH_{2}C{H_{2}}^{*}}+2E_{\mathrm{slab}+H^{*}}-3E_{\mathrm{slab}}-E_{C_{2}H_{6}\left(g\right)}\right]$$ 9

$$\Delta E_{CH_{3}{\mathrm{CH}}^{*}+2H^{*}}=\left[E_{\mathrm{slab}+CH_{3}{\mathrm{CH}}^{*}}+2E_{\mathrm{slab}+H^{*}}-3E_{\mathrm{slab}}-E_{C_{2}H_{6}\left(g\right)}\right]$$ 10

$$\Delta E_{CH_{2}{\mathrm{CH}}^{*}+3H^{*}}=\left[E_{\mathrm{slab}+CH_{2}{\mathrm{CH}}^{*}}+3E_{\mathrm{slab}+H^{*}}-4E_{\mathrm{slab}}-E_{C_{2}H_{6}\left(g\right)}\right]$$ 11

Subsequently, we describe the activation barriers for the minimum energy pathways of the corresponding elementary steps. Adsorbate binding energies are reported for the most stable binding sites, unless otherwise indicated. However, it is important to note that the minimum energy pathway for certain elementary steps may involve reactants or products bound in metastable configurations. In those cases, effective activation barriers, referenced to the energy of the most stable reactant configuration, are reported.

#### 3.2.1 S-Dimer-Covered (001)-S Surface

On the 1 ML S-dimer-covered (001)-S surface, four unique adsorption sites are present: each sulfur atom within the dimer unit (“dim_1_” and “dim_2_”), and two distinct underlying surface sites (“S_1_” and “S_2_”, Figure 6a). H binds most strongly on the dim_1_ site (Figure 7a), with a binding energy (ΔE) of −0.24 eV. C_2_H_6_ is weakly physiosorbed on the surface, exhibiting a ΔE of −0.30 eV (Figure 7b). For monodentate C_2_H_5_, the most stable adsorption occurs at the dimer sites, with ΔE values of −0.02 eV at dim_1_ and 0.06 eV at dim_2_ (Figure 7c,d). When the S–S bond of the S-dimer is dissociated, C_2_H_5_ binds most strongly at the dim_2_ site, with a ΔE of 0.70 eV (Figure 7e). Bidentate CH_2_CH_2_ adsorbs most favorably at the dim_2_-S_2_ site, among 12 possible binding modes, with a ΔE of 0.99 eV (Figure 7f) and a corresponding $\Delta G_{CH_{2}C{H_{2}}^{*}+2H^{*}}$ of 2.98 eV at 1000 K and a gas phase S_2_ pressure of 0.01 atm. Physiosorbed CH_2_CH_2_ has a ΔE of 1.45 eV and a $\Delta G_{CH_{2}C{H_{2}}^{*}+2H^{*}}$ of 2.12 eV under the same conditions. The entropic stabilization of the physiosorbed CH_2_CH_2_ configuration causes the reaction free energy diagram in Figure 9 to feature the $\Delta G_{CH_{2}C{H_{2}}^{*}+2H^{*}}$ of the physiosorbed CH_2_CH_2_ state. Out of 16 configurations, CH_3_CH binds most strongly at the dim_1_-S_1_ and dim_2_-S_2_ bridge sites, with binding energies of 0.92 and 0.91 eV, respectively (Figure 7g,h). Among a total of twenty-eight distinct binding modes, bidentate CH_2_CH has its most preferred binding location (Figure 7i) with the CH_2_- group on the dim_2_ site and the CH- group on the bridge between dim_1_ and S_1_ sites. Both C_2_H_5_ and H can coadsorb on either the dim_1_ or dim_2_ site to form surface-bound C_2_H_5_SH. Interestingly, C_2_H_5_SH is more stable at the dim_2_ site when the S–S bond of the S-dimer is dissociated (ΔE = −0.05 eV, Figure 7k) than when the bond remains intact (ΔE = 0.34 eV). The lowest ΔE for C_2_H_5_-(SS)-H is −0.31 eV, when C_2_H_5_ is bound to the dim_2_ site, and H is adsorbed on the dim_1_ site (Figure 7j). The change in ΔE is negligible when the binding sites of C_2_H_5_ and H are interchanged. Additional information for all binding sites and adsorption energies are provided in Table S6.

**Figure 6:**
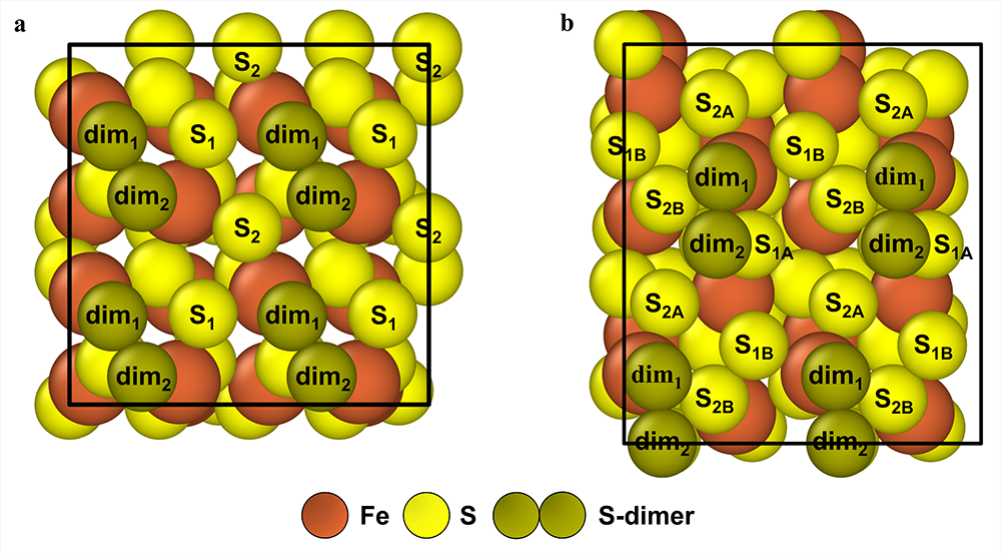
Top views of 1 ML S-dimers adsorbed on (a) Fe–Fe sites on the (001)-S facet, and on (b) Fe–S_1A_ sites on the (210)-2S′ facet, with the identity of the surface sites mentioned.

**Figure 7:**
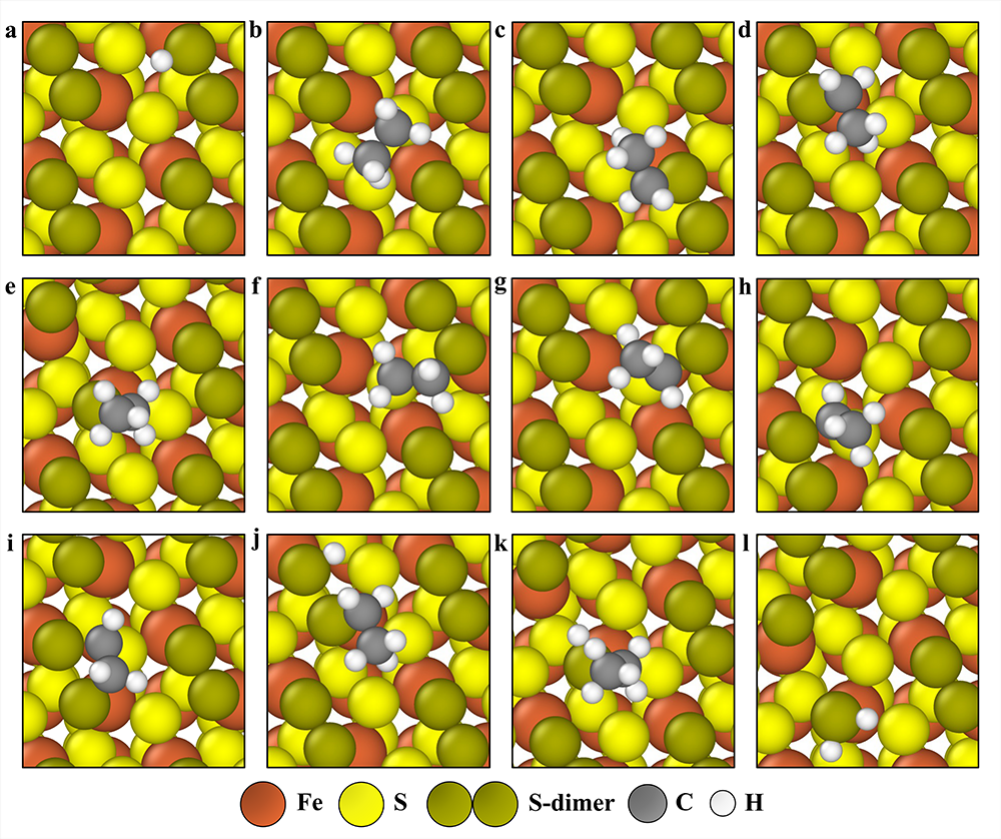
Binding configurations of adsorbates on the 1 ML S-dimer-covered (001)-S surface (all top views), (a) H on the dim_1_ top site, (b) CH_3_CH_3_ physiosorbed, (c) CH_3_CH_2_ on the dim_1_ top site, (d) CH_3_CH_2_ on the dim_2_ top site, (e) CH_3_CH_2_ on the dim_2_ top site, with the S–S bond of S-dimer dissociated, (f) CH_2_CH_2_ on the dim_2_-S_2_ site, (g) CH_3_CH on the dim_2_-S_2_ bridge site, (h) CH_3_CH on the dim_1_-S_1_ bridge site, (i) CH_2_CH on the dim_2_-dim_1_-S_1_ site, (j) CH_3_CH_2_-(SS)-H, CH_3_CH_2_ (dim_2_ site) and H (dim_1_ site) coadsorbed on the S-dimer unit, (k) C_2_H_5_SH on the dim_2_ site (S–S bond of S-dimer dissociated), and (l) surface H_2_S formed from a S-dimer unit (dim_2_ site) with the S–S bond cleaved.

The reaction free energy diagram for S_2_–ODHE on the 1 ML S-dimer covered (001)-S surface at 1000 K and a gas phase S_2_ partial pressure of 0.01 atm is shown in Figure 9. Corresponding diagrams at 800 and 1200 K are provided in the Supporting Information (Figure S15). The ΔG for C_2_H_6_ physisorption is 0.84 eV (ΔE = −0.30 eV), reflecting a significant entropy loss and resulting in an endergonic adsorption process. The minimum energy pathway (MEP) for the first elementary reaction, C_2_H_6_* + * → C_2_H_5_* + H*, features an effective activation barrier (Δ*E*_act,eff_) of 1.84 eV. In this process, C_2_H_5_ adsorbs on the dim_2_ top site, while H binds to a dim_1_ top site of a neighboring S-dimer unit, as shown by the initial, transition, and final state geometries in Figure 8a–c. In the transition state geometry, C_2_H_5_ is positioned slightly farther away from the surface, indicating a less constrained geometry. The corresponding effective free energy barrier (ΔG_act,eff_) for C_2_H_6_ activation is 1.79 eV relative to physiosorbed C_2_H_6_, with a ΔG for this step of 0.97 eV (ΔE = 0.04 eV); this step is therefore likely to be rate limiting. At higher temperatures, ΔG_act,eff_ relative to the gas phase C_2_H_6_ becomes even larger due to the entropy loss associated with the transition from the gas phase to the surface (see Figure S15, Supporting Information).

**Figure 8:**
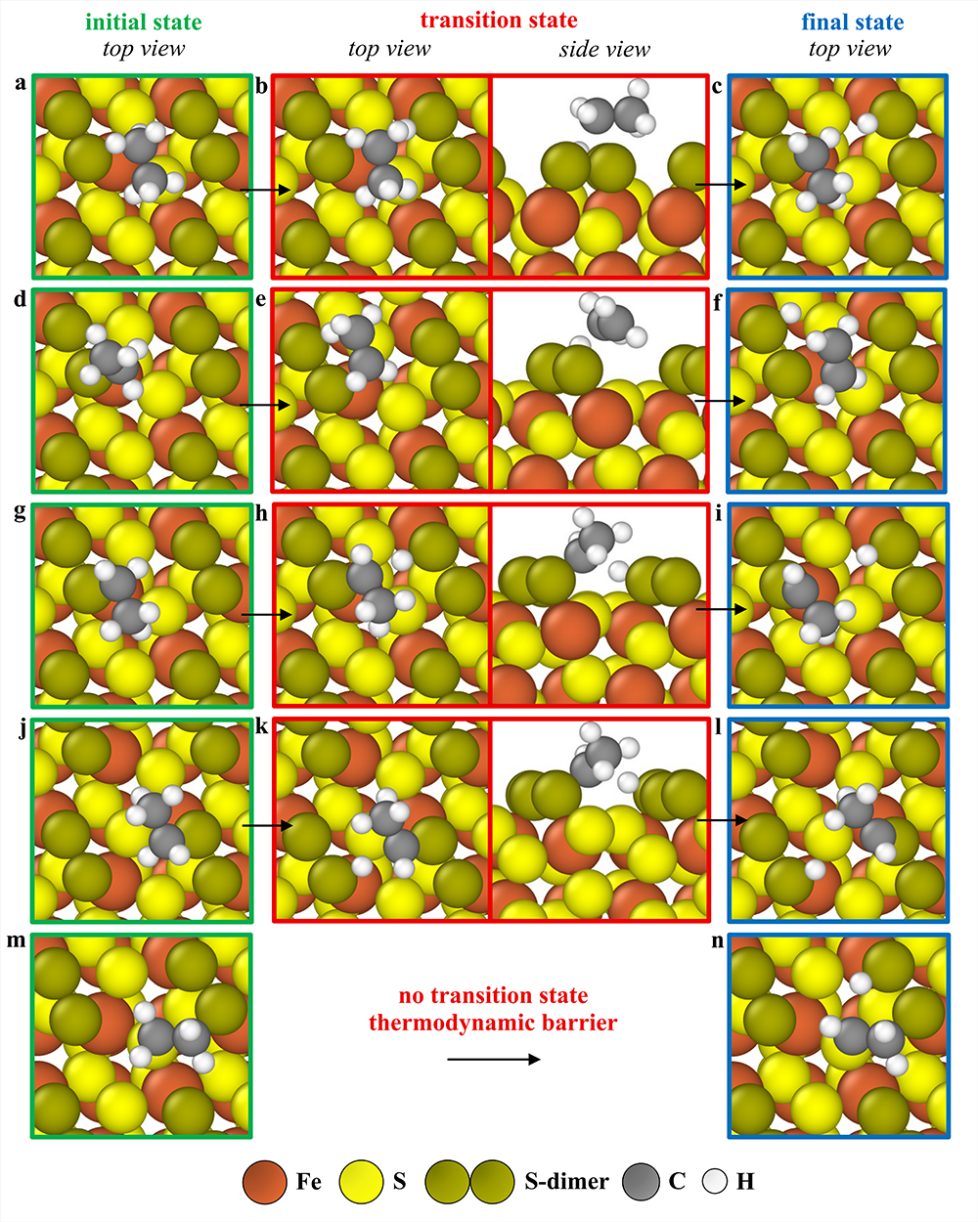
Minimum energy pathways for elementary reactions on the 1 ML S-dimer-covered (001)-S surface. For the reaction CH_3_CH_3_* +*→ CH_3_CH_2_* + H*, (a) initial (top view), (b) transition state (top and side views), and (c) final state (top view) geometries are shown. For the reaction CH_3_CH_2_* +*→ CH_2_CH_2_* + H*, (d) initial (top view), (e) transition state (top and side views), and (f) final state (top view) geometries are provided. For the reaction CH_3_CH_2_* +*→ CH_3_CH* + H*, there are two minimum energy pathways with similar transition state energies. For the first minimum energy pathway, (g) initial (top view), (h) transition state (top and side views), and (i) final state (top view) geometries are given, and for the second minimum energy pathway, (j) initial (top view), (k) transition state (top and side views), and (l) final state (top view) geometries are depicted. For the reaction CH_2_CH_2_* +*→ CH_2_CH* + H*, (m) initial (top view) and (n) final state (top view) geometries are shown.

The subsequent selective C–H bond dissociation step, converting adsorbed C_2_H_5_ to CH_2_CH_2_ and H, has Δ*E*_act,eff_ of 1.59 eV and a ΔG_act,eff_ of 1.01 eV. Here, C_2_H_5_ adsorbed on a dim_2_ top site dissociates to form physiosorbed CH_2_CH_2_ and H adsorbed on the dim_1_ top site of the same S-dimer unit (ΔG = 0.31 eV, Figure 8d–f). As with the previous step, the transition state is not directly constrained on the surface but is geometrically proximal to the physiosorbed CH_2_CH_2_ in the final state. The transition state for the C_2_H_5_* + * → CH_2_CH_2_* + H* reaction is therefore stabilized by an increase in entropy, arising from its floating geometry over the surface and the associated freedom of movement. The free energy of CH_2_CH_2_, formed in the surface-catalyzed pathway, is minimized in its physiosorbed state and has a negligible entropic barrier of desorption (ΔG_desorption_ = −0.90 eV). Deep dehydrogenation of CH_2_CH_2_ to CH_2_CH and H would require physiosorbed CH_2_CH_2_ to adsorb on the surface and undergo further C–H bond activation. This step has a Δ*E*_act,eff_ of approximately 0.73 eV and a ΔG_act,eff_ of 2.11 eV. This elementary step proceeds with CH_2_CH_2_ binding on the dim_2_-S_2_ site (Figure 8m), and upon C–H bond breaking, H moves to the adjacent dim_1_ site (Figure 8n). This energy barrier is thermodynamic and does not have an overbarrier. However, further diffusion of CH_2_CH and H to their most stable sites would make the free energy of the system lower (ΔG = 0.96 eV, with respect to physiosorbed CH_2_CH_2_), as shown in Figure 9. Therefore, deep dehydrogenation of CH_2_CH_2_ remains uncompetitive compared to its desorption.

**Figure 9:**
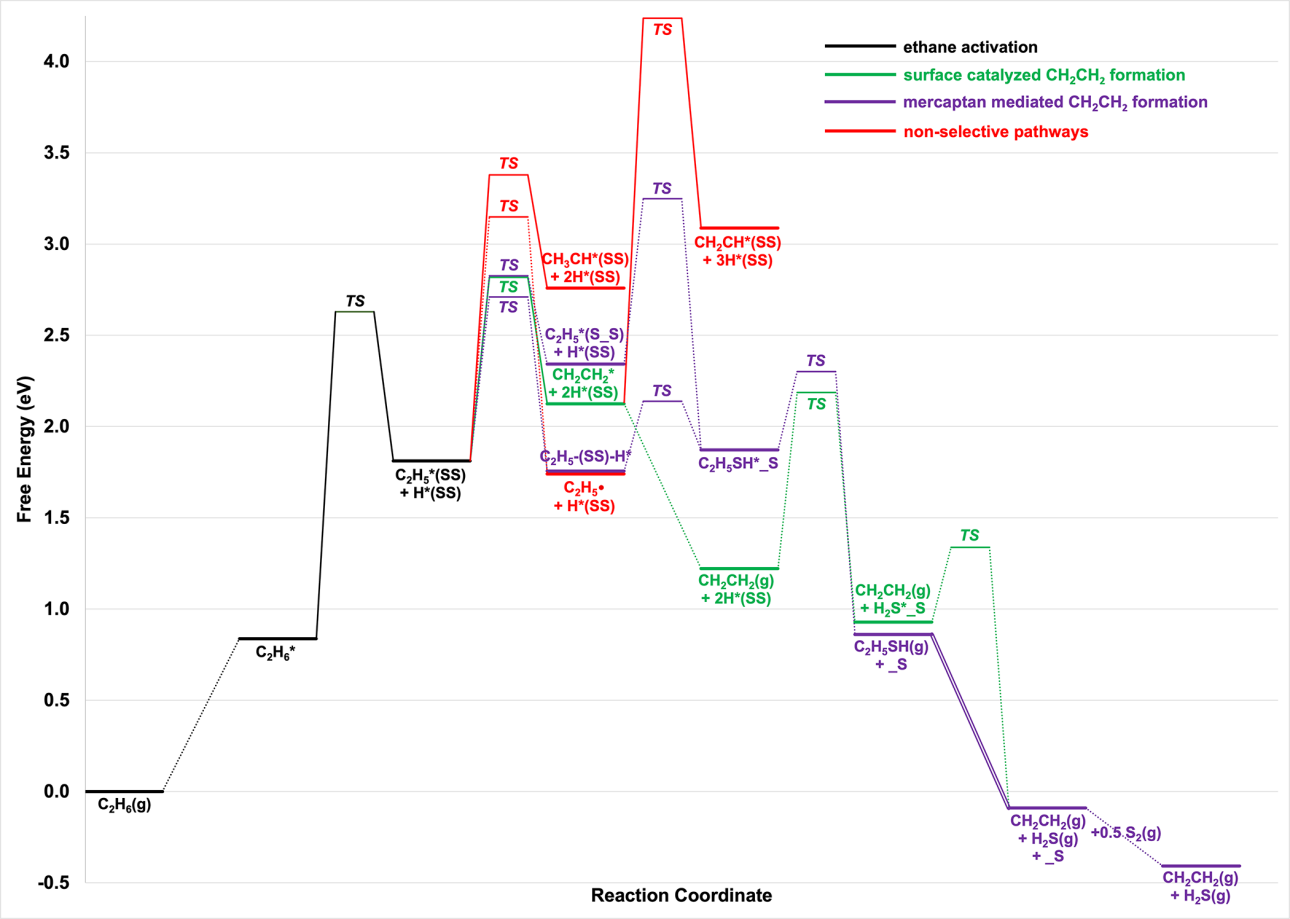
Reaction free energy diagram of S_2_–ODHE on a 1 ML S-dimer-covered (001)-S facet at 1000 K and a partial pressure of 0.01 atm for gas phase S_2_. Under these conditions, the selective pathways for CH_2_CH_2_ formation are favorable. Both the mercaptan-mediated and surface-catalyzed pathways for CH_2_CH_2_ formation are competitive at these conditions.

After desorption of CH_2_CH_2_, two hydrogen atoms located at their most stable sites require a ΔG_act,eff_ of 0.96 eV to diffuse and form a surface H_2_S species (Figure 7l), a process that also effectively involves S–S bond dissociation (ΔG of −0.29 eV). Surface H_2_S has a desorption barrier of 0.41 eV with a ΔG_desorption_ of −1.02 eV. Subsequent replenishment of the surface S-vacancy by gaseous S_2_ is thermodynamically favorable, with a ΔG of −0.32 eV.

The nonselective C–H bond dissociation step, converting C_2_H_5_ to CH_3_CH and H, has a Δ*E*_act,eff_ of approximately 1.51 eV and a ΔG_act,eff_ of 1.57 eV. The transition state is close to the surface. This step can proceed via two antipodal pathways. In one such pathway, C_2_H_5_ is initially adsorbed on a dim_2_ top site; upon dissociation, CH_3_CH remains on the same dim_2_ site while H migrates to an adjacent dim_1_ site of a neighboring S-dimer unit (Figure 8g–i). In the alternative pathway, both the initial C_2_H_5_ and final CH_3_CH are located on a dim_1_ site, with the dissociated H moving to a dim_2_ site on an adjacent S-dimer unit (ΔG = 0.88 eV) (Figure 8j–l). Although the selective and nonselective C–H bond breaking steps exhibit comparable Δ*E*_act,eff_ values, the transition state entropy, dictated by the respective geometries, renders the selective C–H bond cleavage pathway significantly more favorable than the nonselective route.

After investigating the primary C–H dissociation steps of C_2_H_5_, we examined the mercaptan-mediated pathway in detail, as illustrated in Figure 5. S–S bond dissociation of a S-dimer unit without any adsorbate has a Δ*E*_act,eff_ of 1.43 eV. Binding of C_2_H_5_ at the dimer sites weakens the S–S bond within the S-dimer unit, and consequently, S–S bond dissociation in the presence of C_2_H_5_ (Figure 7d,e) has a Δ*E*_act,eff_ of 1.11 eV and a ΔG_act,eff_ of 1.01 eV, with a ΔG of 0.53 eV (ΔE = 0.72 eV). S–S bond dissociation enables the surface species to achieve a modest increase in entropy. H-diffusion from a nearby dim_1_ site to form the coadsorbed C_2_H_5_-(SS)-H configuration (Figure 7j) from a C_2_H_5_-(SS) configuration (Figure 7d) has both a Δ*E*_act,eff_ and a ΔG_act,eff_ of 0.90 eV, with a ΔG_diffusion_ of −0.06 eV. Both steps exhibit similar free energy barriers compared to the competing selective pathway from C_2_H_5_ to CH_2_CH_2_. Once the S–S bond is dissociated, with C_2_H_5_ bound (Figure 7e), H diffusion from an adjacent dim_1_ site is required to form surface-bound C_2_H_5_SH_S species (Figure 7k), with both a Δ*E*_act,eff_ and a ΔG_act,eff_ of 0.91 eV. S–S bond dissociation in the C_2_H_5_-(SS)-H configuration, followed by a short H hop to form C_2_H_5_SH, results in an even lower Δ*E*_act,eff_ of 0.47 eV and a ΔG_act,eff_ of 0.38 eV. These results indicate that coadsorption of both C_2_H_5_ and H on the same S-dimer unit further weakens the S–S bond, facilitating the formation of surface-bound ethyl mercaptan. The desorption barrier for C_2_H_5_SH from the surface is 0.43 eV, with a ΔG_desorption_ of −1.01 eV. Entropic gain due to high temperature and low partial pressure makes C_2_H_5_SH desorption to the gas phase thermodynamically favorable. A solid double line is used to indicate the conversion corresponding to the gas phase decomposition of C_2_H_5_SH, which has a ΔG of −0.95 eV. Furthermore, the nonselective desorption barrier for the C_2_H_5_ radical is 1.34 eV, with a ΔG_desorption_ of −0.07 eV.

The reaction free energy diagram indicates that, starting from a C_2_H_5_ adsorbate, the pathway to surface bound C_2_H_5_SH is competitive with the selective surface-catalyzed conversion of C_2_H_5_ to CH_2_CH_2_ at 1000 K and an S_2_ partial pressure of 0.01 atm. Under these conditions, nonselective C–H bond dissociation to CH_3_CH and C_2_H_5_ radical desorption are less energetically favorable. At even higher temperatures, however, the enhanced entropy associated with C_2_H_5_ radical desorption reduces the free energy gap between the radical desorption barrier and the barriers for mercaptan-mediated and surface-catalyzed CH_2_CH_2_ formation pathways (Figure S15b). Deeper dehydrogenation, and any associated surface C–C bond cleavage, is unlikely to be competitive, given the favorable energetics of CH_2_CH_2_ desorption. Though the majority gas phase decomposition products for C_2_H_5_SH are CH_2_CH_2_ and H_2_S, some nonselective side products are also formed.^32^ Table S11 summarizes the Δ*E*_act,eff_ values for all surface mediated elementary reaction steps.

#### 3.2.2 S-Dimer-Covered (210)-2S′ Surface

As mentioned above, on the 1 ML S-dimer-covered (210)-2S′ surface, there are two S-dimer sites (dim_1_ and dim_2_), as well as other underlying sites from the pristine (210)-2S′ surface (Figure 6b). Atomic H binds most strongly at the dim_1_ site (Figure 10a), with a ΔE of −0.17 eV, while physiosorbed C_2_H_6_ has a ΔE of −0.25 eV (Figure 10b). The most stable adsorption for monodentate C_2_H_5_, in turn, occurs at the dim_1_ site, with a ΔE of 0.04 eV (Figure 10c). When the S–S bond of the S-dimer is dissociated, however, C_2_H_5_ binds even more strongly to the dim_1_ site (ΔE of 0.52 eV; Figure 10d). Bidentate CH_2_CH_2_ binds most favorably at the dim_1_-S_1B_ site, with a ΔE of 1.02 eV (Figure 10e), a corresponding $\Delta G_{CH_{2}C{H_{2}}^{*}+2H^{*}}$ of 3.20 eV at 1000 K, and a gas phase S_2_ pressure of 0.01 atm. Physiosorbed CH_2_CH_2_ has a ΔE of 1.45 eV and a $\Delta G_{CH_{2}C{H_{2}}^{*}+2H^{*}}$ of 2.30 eV under the same conditions. Therefore, the reaction free energy diagram in Figure 12 features the $\Delta G_{CH_{2}C{H_{2}}^{*}+2H^{*}}$ of the physiosorbed CH_2_CH_2_ state. CH_3_CH binds most strongly at the dim_2_-S_1A_ bridge site, with a ΔE of 0.50 eV (Figure 10f). Bidentate CH_2_CH has its strongest binding at a dim_1_-dim_2_ site, with a ΔE of 1.44 eV (Figure 10g). The most stable C_2_H_5_–(SS)–H configuration (Figure 10h) occurs when C_2_H_5_ is bound to the dim_1_ site and H is adsorbed on the dim_2_ site (ΔE of −0.08 eV). However, coadsorption of C_2_H_5_ and H on the S-dimer unit induces distortion of the S-dimer structure, causing the dim_2_ site to shift slightly away from the underlying S_1A_ site. Adsorbed C_2_H_5_SH is less stable when the S–S bond of the S-dimer remains intact (ΔE = 0.47 eV) than when it is dissociated (ΔE = −0.25 eV, Figure 10i). Additional details for all binding sites and adsorption energies are provided in Table S7.

**Figure 10:**
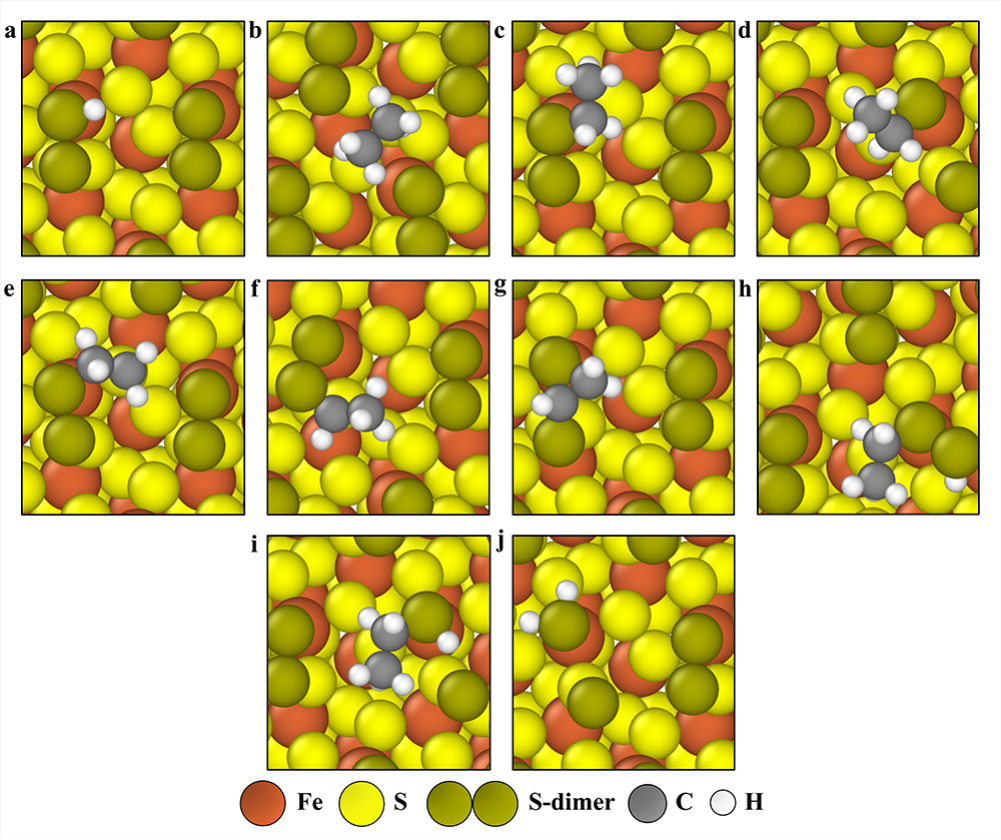
Binding configurations of adsorbates on the 1 ML S-dimer-covered (210)-2S′ surface (all top views), (a) H on the dim_1_ top site, (b) CH_3_CH_3_ physiosorbed, (c) CH_3_CH_2_ on the dim_1_ top site, (d) CH_3_CH_2_ on the dim_1_ top site, with the S–S bond of S-dimer dissociated, (e) CH_2_CH_2_ on the dim_1_-S_1B_ site, (f) CH_3_CH on the dim_2_-S_1A_ bridge site, (g) CH_2_CH on the dim_1_-dim_2_ site, (h) CH_3_CH_2_–(SS)-H, CH_3_CH_2_ (dim_1_ site) and H (dim_2_ site) coadsorbed on the S-dimer unit, (i) C_2_H_5_SH on the dim_1_ site (S–S bond of S-dimer dissociated), and (j) surface H_2_S formed from a S-dimer unit (dim_1_ site) with the S–S bond cleaved.

Figure 12 illustrates the reaction free energy diagram for S_2_–ODHE on a 1 ML S-dimer-covered (210)-2S′ surface at 1000 K and a gas phase S_2_ partial pressure of 0.01 atm (additional diagrams at 800 and 1200 K are provided in the Supporting Information Figure S16). The ΔG for C_2_H_6_ physisorption is 0.79 eV (ΔE = −0.25 eV), influenced by the significant loss of entropy during adsorption. The first elementary reaction, C_2_H_6_* +*→ C_2_H_5_* + H*, proceeds via two pathways with similar Δ*E*_act,eff_ values, 1.83 and 1.91 eV, which are comparable to the Δ*E*_act,eff_ value for the same step on the S-dimer-covered (001)-S surface. In both pathways, C_2_H_5_ is adsorbed on the dim_1_ top site. For the first pathway, the dissociated H binds on the dim_1_ top site of a neighboring S-dimer (Figure 11a–c), whereas in the second pathway, H occupies the adjacent S_1B_ site (Figure 11d–f). In either case, the transition state geometry features C_2_H_5_ slightly farther from the surface than is typical for the adsorbed reaction intermediates, suggesting that the transition states have more degrees of freedoms than the surface adsorbates. Nevertheless, the ΔG_act,eff_ for C_2_H_6_ activation is 2.22 eV relative to physiosorbed C_2_H_6_, which is slightly higher than that on the S-dimer-covered (001)-S surface due to additional entropy loss when transitioning from the physiosorbed state to the transition state geometry on (210)-2S′. The high value of ΔG_act,eff_ strongly suggests that this initial C–H bond activation is the rate controlling step for S_2_–OEDH on this facet.

**Figure 11:**
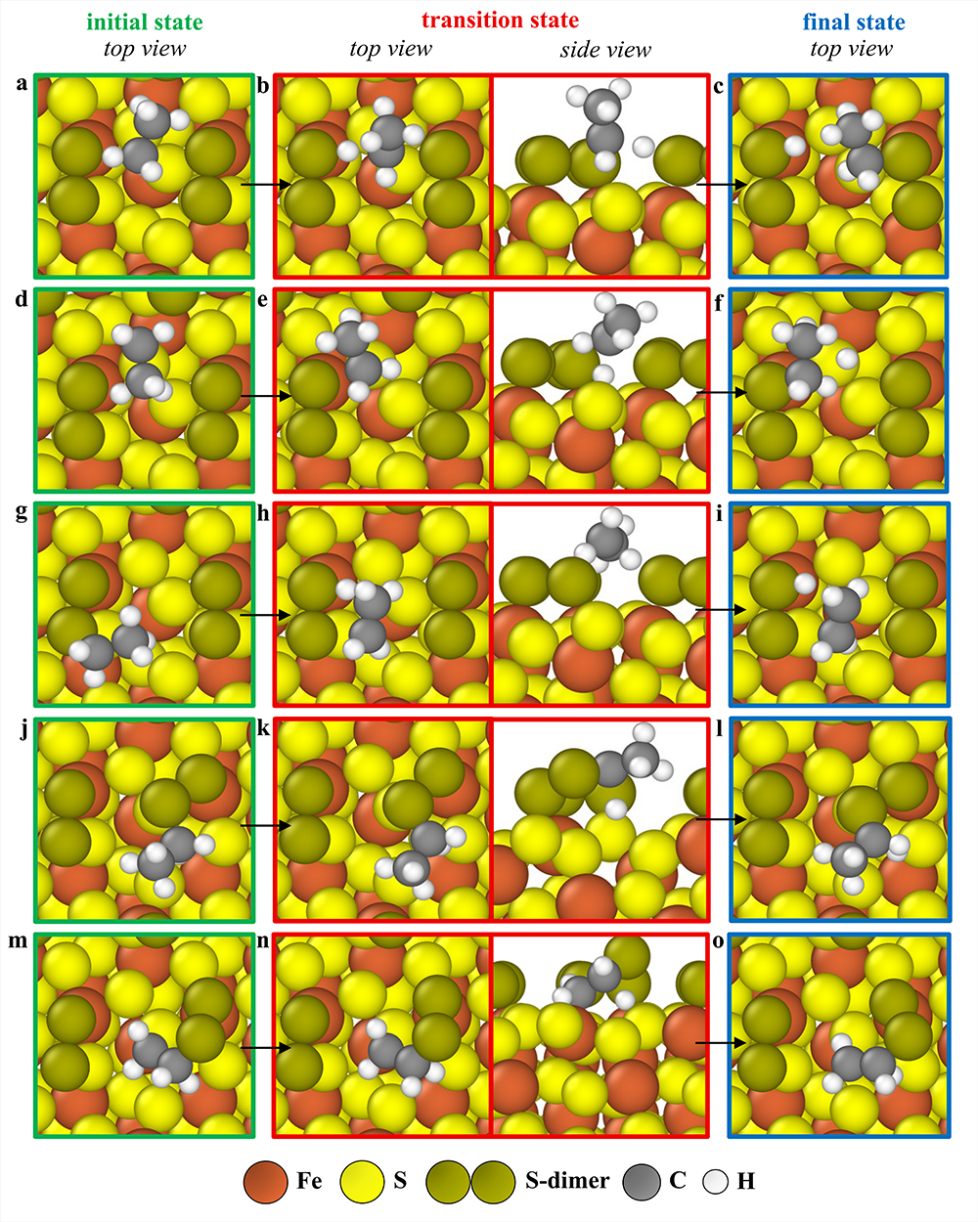
Minimum energy pathways for elementary reactions on the 1 ML S-dimer-covered (210)-2S′ surface. For the reaction CH_3_CH_3_* +*→ CH_3_CH_2_* + H*, there are two minimum energy pathways with similar transition state energies. For the first minimum energy pathway, (a) initial (top view), (b) transition state (top and side views), and (c) final state (top view) geometries are provided, and for the second minimum energy pathway, (d) initial (top view), (e) transition state (top and side views), and (f) final state (top view) geometries are shown. For the reaction CH_3_CH_2_* +*→ CH_2_CH_2_* + H*, (g) initial (top view), (h) transition state (top and side views), and (i) final state (top view) geometries are given. For the reaction CH_3_CH_2_* +*→ CH_3_CH* + H*, (j) initial (top view), (k) transition state (top and side views), and (l) final state (top view) geometries are depicted. For the reaction CH_2_CH_2_* +*→ CH_2_CH* + H*, (m) initial (top view), (n) transition state (top and side views), and (o) final state (top view) geometries are shown.

The next selective C–H bond dissociation step, leading from adsorbed C_2_H_5_ to CH_2_CH_2_ and H, proceeds via a pathway with a Δ*E*_act,eff_ of 1.44 eV and a ΔG_act,eff_ of 0.75 eV. Here, C_2_H_5_ bound at a dim_2_ top site undergoes dissociation to yield physiosorbed CH_2_CH_2_ alongside an H atom adsorbed on the dim_1_ top site of the same S-dimer (ΔG = 0.26 eV, Figure 11g–i). The corresponding geometries closely resemble those for the same elementary reaction on the S-dimer covered (001)-S surface (Figure 11d–f). As before, the late transition state geometry, closely resembling the physiosorbed CH_2_CH_2_ configuration, results in higher entropy, which lowers the free energy barrier of this step. Physiosorbed CH_2_CH_2_ has no entropic desorption barrier and has a ΔG_desorption_ of −0.87 eV, suggesting that such desorption is facile. The competitive, nonselective pathway CH_2_CH_2_* + * → CH_2_CH* + H* has a Δ*E*_act,eff_ of 0.83 eV and a ΔG_act,eff_ of 2.15 eV. For this elementary step, physiosorbed CH_2_CH_2_ is adsorbed on the dim_2_-S_2B_ site on a distorted S-dimer structure. Upon C–H bond activation, H moves to the underlying S_1A_ site (ΔG = 1.20 eV, Figure 11m–o). As with the S-dimer covered (001)-S surface, deep dehydrogenation of CH_2_CH_2_ is energetically unfavorable on the S-dimer covered (210)-2S′ surface.

After desorption of CH_2_CH_2_, two hydrogen atoms located at their most stable sites require a ΔG_act,eff_ of 1.30 eV to diffuse and form a surface H_2_S species (Figure 10j), a process that also effectively involves S–S bond dissociation (ΔG of −0.72 eV). Surface H_2_S has a desorption barrier of 0.23 eV with a ΔG_desorption_ of −1.21 eV. Subsequent replenishment of the surface S-vacancy by gaseous S_2_ has a ΔG of 0.08 eV.

The nonselective C–H bond dissociation step, in which C_2_H_5_ dissociates to CH_3_CH and H, has a Δ*E*_act,eff_ of 1.14 eV and a ΔG_act,eff_ of 1.13 eV, indicating negligible change in entropy. In this pathway, C_2_H_5_ initially adsorbs at a dim_2_ site, and upon C–H cleavage, the released H atom migrates to an underlying S_1A_ site on the surface (ΔG = 0.49 eV, Figure 11i–k). CINEB calculations reveal that, to enable H access to the S_1A_ site, the S-dimer associated with the C_2_H_5_ adsorption site must be distorted from its preferred flat geometry. In spite of this local distortion, however, the corresponding transition state has the lowest energy barrier among all pathways considered for this elementary reaction. The nonselective C–H bond breaking step has a lower Δ*E*_act,eff_ than the selective C–H bond breaking step. However, the entropic disparity associated with the transition state makes the free energy barrier of the selective pathway more favorable.

On the 1 ML S-dimer covered (210)-2S′ surface, two mercaptan-mediated pathways also have favorable energetics. In the first such pathway, ethane dissociates to yield a C_2_H_5_ intermediate on a S-dimer site, followed by dissociation of the S–S bond and bonding with surface hydrogen to yield adsorbed C_2_H_5_SH: C_2_H_5_*–(SS) → C_2_H_5_*–(S_S) → C_2_H_5_SH*_S. Alternatively, both C_2_H_5_ and H can bond to an intact S-dimer group, followed by H insertion into the S–S bond to produce C_2_H_5_SH: C_2_H_5_*–(SS) → C_2_H_5_*–(SS)–H → C_2_H_5_SH*_S. As observed in Figure 12, both mercaptan pathways have effective free energies of activation comparable to the ΔG_act,eff_ of the surface-catalyzed C_2_H_5_* + * → CH_2_CH_2_* + H* pathway, and lower than ΔG_act,eff_ for the nonselective C_2_H_5_* + * → CH_3_CH* + H* pathway. C_2_H_5_SH desorption is associated with an entropic barrier of 0.28 eV and a ΔG_desorption_ of −1.16 eV, reflecting the substantial entropy gain upon transition to the gas phase. Finally, the desorption of a C_2_H_5_ radical has a barrier of 1.20 eV, which is somewhat unfavorable compared to the other selective pathways leading from surface C_2_H_5_. At the conclusion of the reaction cycle, surface sulfur vacancy replenishment occurs with a ΔG of 0.08 eV from the gas phase.

**Figure 12:**
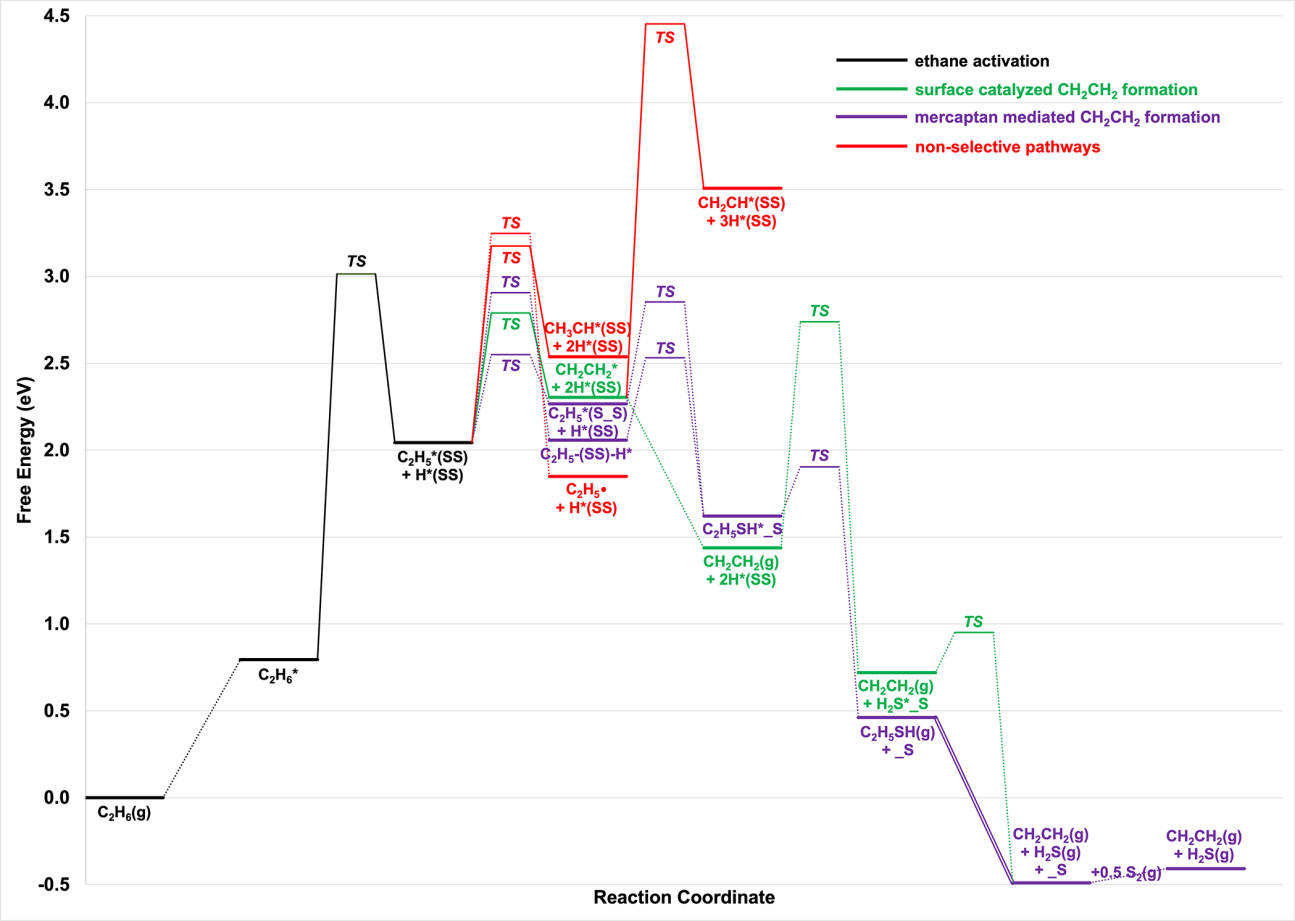
Reaction free energy diagram of S_2_–ODHE on a 1 ML S-dimer-covered (210)-2S′ facet at 1000 K and a partial pressure of 0.01 atm for gas phase S_2_. Under these conditions, the selective pathways for CH_2_CH_2_ formation are favorable. Both the mercaptan-mediated and surface-catalyzed pathways for CH_2_CH_2_ formation are competitive at these conditions.

As with the above results, reaction energy analysis of the S-dimer-covered (210)-2S′ surface shows that selective pathways for CH_2_CH_2_ formation, mercaptan mediation, and direct surface-catalyzed formation, are more energetically favorable than other nonselective chemical routes. As noted previously, observed byproducts could result from nonselective dissociation of gas phase C_2_H_5_SH.

#### 3.2.3 Pristine (001)-S, (210)-2S′, and (111)-3S Surfaces

To understand how S-dimer structures influence reaction energetics and mechanisms, we compare the results on the surfaces discussed above, where excess S_2_ adspecies are generally present on the surface, to ethane reaction on (001)-S and (210)-2S′ surfaces with no additional S_2_ adspecies. The pristine (111)-3S surface, which offers a distinct sulfur-rich environment, is also considered. These analyses, across multiple surface structures with varying degrees of sulfidation, enhance understanding of the role surface sulfur atoms play in directing selective and nonselective pathways.

Tables S8–S10 summarize the binding sites and adsorption energies of surface adsorbates on the (001)-S, (210)-2S′, and (111)-3S surfaces, respectively. The geometries of the most stable adsorbate sites are illustrated in Figures S17–S19. Detailed information on the examined surface reaction pathways and associated energy barriers can be found in Tables S13–S15 for the (001)-S, (210)-2S′, and (111)-3S surfaces, respectively. Corresponding minimum energy pathway geometries are presented in Figures S20–S22.

Figure S23b presents the reaction free energy diagram for the pristine (001)-S surface at 1000 K and an S_2_ partial pressure of 0.01 atm. At this temperature, the ΔG_act,eff_ for the initial C–H bond activation relative to gas phase C_2_H_6_ is high, measuring 2.83 eV with respect to physiosorbed C_2_H_6_*. After the formation of surface-bound C_2_H_5_, desorption is almost equally favored over conversion to surface C_2_H_5_SH or CH_2_CH_2_ via selective C–H bond dissociation, which can be attributed to the weaker binding of C_2_H_5_ on the (001)-S surface. Nonselective C–H bond cleavage to CH_3_CH is unfavorable compared to all other pathways.

Figure S24b presents the reaction free energy diagram under similar conditions for the (210)-2S′ surface. The rate-limiting C_2_H_6_ activation step has a ΔG_act,eff_ of 2.23 eV relative to physiosorbed C_2_H_6_. On this surface, H diffusion leading to the formation of surface C_2_H_5_SH is slightly more favorable than other C–H bond dissociation steps starting from surface C_2_H_5_. However, due to the strong binding of C_2_H_5_SH and the high S-vacancy formation energy associated with C_2_H_5_SH, the desorption barrier for C_2_H_5_SH is relatively high. As a result, the combined energy barrier for mercaptan formation and desorption is likely prohibitive, favoring the surface C–H bond dissociation pathways instead. The energy barriers for the competing pathways from C_2_H_5_ to CH_2_CH_2_ and CH_3_CH are nearly equivalent, creating a unique environment where both selective and nonselective routes are almost equally favored. Notably, desorption of the C_2_H_5_ radical on the (210)-2S′ surface is not energetically competitive.

Figure S25b presents the reaction free energy diagram under similar conditions for the (111)-3S surface, where the ΔG_act,eff_ for C_2_H_6_ activation is 1.66 eV relative to physiosorbed C_2_H_6_. On this surface, the selective, surface-catalyzed formation of CH_2_CH_2_ from C_2_H_5_ is the most favorable pathway. Strong hydrogen adsorption on the (111)-3S surface makes H-diffusion energetically difficult, thereby rendering the mercaptan mediated pathway less favorable compared to other surface pathways. This phenomenon is likely to increase ethylene selectivity on the (111)-3S surface.

### 3.3 Reactivity Trends and Activity Descriptor

This study systematically investigates the reaction mechanisms for oxidative dehydrogenation of ethane across FeS_2_ surface structures exhibiting varying degrees of surface sulfidation. For all such surfaces and mechanisms, the initial C–H bond activation of ethane emerges as a likely rate-determining step. Previous studies on sulfur-assisted methane activation over metal sulfide catalysts have demonstrated that C–H bond activation is strongly influenced by the metal–sulfur bond strength, with weaker metal–sulfur bonds conferring greater basicity and enabling more facile hydrogen abstraction.^17^ Similarly, sulfur oxidative propane dehydrogenation studies have shown a correlation between the experimental apparent activation energy for propane and the sulfur *p-*band center, as measured by XPS, across a range of metal sulfides.^22^ Analogous trends have been identified in metal oxide catalysis, where the average oxygen 2*p* state energy correlates with oxygen evolution reaction activity^56^ and, in alumina-supported vanadium oxide catalysts, the oxygen *p*-band center has been found to align with the dehydrogenation barrier on oxygen sites.^57^

In a similar fashion, the average valence *p*-state energy of surface sulfur atoms, which is in turn related to the sulfur vacancy formation energy, can serve as a meaningful activity descriptor for S_2_–OEDH. Correlations of the effective activation barrier with the average valence *p*-state energy, as well as with the vacancy formation energy (determined via single-point calculations, removing the relevant sulfur atom from the slab model without structural relaxation), suggest that weaker bound and more basic surface sulfur atoms facilitate alkane C–H bond activation (Figure 13). Across all FeS_2_ surfaces analyzed, including dim_1_ sites on S-dimer covered surfaces (Figure 6), S sites on (001)-S, S_1A_ sites on (210)-2S′ (Figure 2), and S_1_ sites on (111)-3S (Figure S3), the most weakly bound sulfur participated directly in the minimum energy pathway for the rate limiting step. The R^2^ values for correlations with the sulfur vacancy formation energy, and with the average *p*-state energy of the most weakly bound surface sulfur atoms, are 0.57 and 0.85, respectively. Removing a sulfur atom to generate a vacancy can break one or multiple metal–sulfur and sulfur–sulfur bonds, depending sensitively on the local coordination environment of that surface site. As a result, the S-vacancy formation energy is a complex metric that does not fully capture the intrinsic catalytic character of the active sites. In contrast, the average valence *p*-state energy of surface sulfur atoms directly encodes the electronic coupling between the surface sites, the adsorbates, and the relevant reaction transition states, and the higher R^2^ value indicates that the average *p*-state energy of the most weakly bound surface sulfur atoms is a robust activity descriptor for S_2_–OEDH. We note, in passing, that these concepts are relatively broad and could potentially be extended to develop a more general activity descriptor for S_2_–OEDH on transition metal sulfide surfaces.

**Figure 13:**
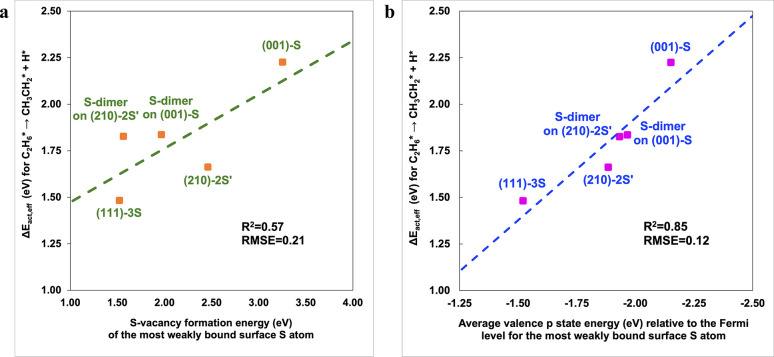
Δ*E*_act,eff_ for the C_2_H_6_* +*→ CH_3_CH_2_* + H* reaction vs (a) sulfur vacancy formation energy and (b) the average valence *p*-state energy of the weakest bound surface sulfur atom.

## 4 Discussion

In this study, DFT-based theoretical investigations have elucidated the surface structures of FeS_2_ across a spectrum of temperatures and operating pressures. The thermodynamic stability, in the form of a grand canonical surface free energy, of different sulfur-decorated motifs on low energy, bulk-terminated FeS_2_ facets has been evaluated in S_2_-rich gaseous environments. Under typical S_2_–OEDH conditions of 850 to 1050 K and S_2_ partial pressure of 0.01 atm, the (001)-S and (210)-2S′ surfaces are predominantly covered by varying coverages of S-dimer species, which can form via direct adsorption from the S_2_ gas stream. At temperatures above 1000–1050 K, however, both facets may expose pristine surfaces free of sulfur defects, while below 850 K, heavier sulfur aggregates, such as S-trimers and S-tetramers, become thermodynamically stable, although these configurations are unlikely to persist under reaction conditions and in the presence of carbonaceous species. In all cases, the periodic array of exposed Fe atoms on the (001)-S and (210)-2S′ facets strongly stabilizes S-dimer structures. Further, literature demonstrates that peroxide (O_2_^2–^) sites on metal oxide materials can profoundly affect catalytic performance, such as by influencing H_2_ and CO oxidation activity on CuO^58^ and improving selectivity for ethylene epoxidation on AgO surfaces.^59^ Analogously, S-dimers have been identified as dominant surface species on RuS_2_, where they play a central role in methane-H_2_S reforming,^60^ and S-dimer sites on FeS_2_ surfaces have been found to be catalytically important for sulfur-oxidative coupling of methane.^19^ These observations, together with the result that the S_2_ dimer population is strongly dependent upon both temperature and the FeS_2_ surface facet, suggest that careful control of the population of these dimers may be needed for optimizing S_2_–OEDH reactivity.

Analysis of reaction energetics reveals that, on the S-dimer-covered (001)-S and (210)-2S′ surfaces, both the mercaptan mediated pathway and conventional direct surface-catalyzed ethylene formation pathways remain competitive at 1000 K and an S_2_ partial pressure of 0.01 atm. The mercaptan-mediated mechanism proceeds via surface recombination through sequential interchangeable H diffusion and S–S bond dissociation steps to form C_2_H_5_SH, followed by its desorption. Literature demonstrates that the predominant decomposition products of gas phase C_2_H_5_SH are C_2_H_4_ and H_2_S,^32^ along with small amounts of C_1_ compounds such as CS_2_ and CH_4_, which are reported as primary and side products in S_2_–OEDH, respectively.^20^

Surface-catalyzed nonselective C–H bond dissociation pathways are, generally, found to be uncompetitive on both S-dimer-covered facets. The first nonselective pathway converting C_2_H_5_ to CH_3_CH is energetically less favorable than either the mercaptan-mediated pathway or surface-catalyzed CH_2_CH_2_ formation. The second nonselective pathway, in which CH_2_CH_2_ undergoes further deep dehydrogenation via a surface-catalyzed route, is also energetically unfavorable because CH_2_CH_2_ immediately enters a physisorbed state following C–H bond dissociation in C_2_H_5_, thereby avoiding additional entropic barriers for desorption. Consequently, nonselective products most likely emerge from a gas phase C_2_H_5_SH decomposition processes, and both the selective and nonselective pathways by which such gas phase chemistry occurs are a promising topic for future study. Experimental observations reporting side products such as CH_4_ and CS_2_ from C_2_H_5_SH decomposition support the hypothesis that gas phase C–C bond cleavage contributes to diminished ethylene selectivity.

Mechanistic investigations of the underlying pristine (001)-S and (210)-2S′ surfaces, which may be present at high temperatures, show that, on pristine (001)-S, nonselective C_2_H_5_ radical desorption is equally favorable to subsequent selective C–H bond dissociation or mercaptan formation steps. On pristine (210)-2S′, the mercaptan-mediated pathway is not viable, and surface-catalyzed selective and nonselective pathways are equally preferred. The radical desorption and nonselective C–H bond dissociation pathways on the pristine surfaces are likely to contribute to formation of nonselective products. On the other hand, the highly S-enriched (111)-3S surface favors the surface-catalyzed pathway for CH_2_CH_2_ formation.

Selectivity in S_2_–OEDH is broadly governed by a delicate interplay of geometric accessibility, electronic structure, and surface diffusion dynamics, with each pathway exhibiting distinct features. For the selective C–H bond dissociation from C_2_H_5_ to CH_2_CH_2_, the transition state is not in close proximity to the surface and resembles a physiosorbed CH_2_CH_2_ state. As a result, geometric proximity, and the accessibility of an adjacent site for the dissociating hydrogen atom of the adsorbed CH_3_CH_2_ configuration, are crucial to determining the barrier for this selective pathway. In contrast, the selective C–H bond dissociation from C_2_H_5_ to CH_3_CH occurs near the surface, originating from the CH_2_– group. Here, the activation barrier is strongly governed by the electronic properties of the surrounding surface sulfur atoms. For the mercaptan-mediated pathway, atomic hydrogen diffusion emerges as a critical step. This process is controlled by both the binding energy of hydrogen and its migration route from the most stable binding site to form surface C_2_H_5_SH. During this migration, hydrogen atoms encounter a variety of S-rich diffusion environments, each shaping the overall diffusion barrier. Finally, nonselective desorption of C_2_H_5_ is dictated primarily by its surface binding energy, rather than by more complex geometric or electronic interactions.

We note that our previous experimental S_2_–OEDH study reports a first-order dependence on ethane and a half-order dependence on S_2_.^20^ The reaction energy diagrams suggest that the initial C–H bond activation of ethane is the rate-controlling step, consistent with an overall first-order dependence on ethane. The same experimental work attributes the half-order dependence on S_2_ to the relatively weak dissociative adsorption of S_2_ required to replenish surface sulfur sites. On S-dimer covered surfaces, the reaction energy diagrams identify a mercaptan-mediated channel as a significant contributor to ethylene formation, involving S–S bond scission within the S-dimer and formation of surface C_2_H_5_SH. This pathway naturally links S_2_ dissociation with the formation of reactive intermediates and may, thus, provide a mechanistic rationale for the observed half-order dependence on S_2_.

In technical catalytic systems, multiple bulk-terminated facets with distinct coordination environments are likely to be exposed under reaction conditions. Under sulfur-rich atmospheres, these varied surface geometries undergo sulfidation, resulting in diverse sulfur-decorated surface structures. The activity descriptor identified in this study indicates that, generally, increased surface sulfidation weakens the binding strength of surface sulfur atoms, thereby lowering the activation barrier for the rate-determining step. However, sulfur-rich surfaces tend to be favored at lower temperatures, where catalytic turnover rates may be limited by thermal activation. Consequently, there exists an opportunity to optimize FeS_2_ catalyst performance for S_2_–OEDH by tuning reaction parameters to balance surface sulfidation and activity, potentially identifying an optimal temperature and pressure window. Furthermore, exploration of nonstoichiometric, sulfur-deficient pyrrhotite phases, which can form from FeS_2_ under elevated temperatures and reduced sulfur partial pressures, presents a promising avenue for surface engineering. These pyrrhotite compounds offer significant potential for tailoring surface sulfidation and catalytic properties in Fe–S based systems.

## 5 Conclusions

This study provides a detailed theoretical investigation into the surface structures and reaction mechanisms of FeS_2_ catalysts for the soft oxidative dehydrogenation of ethane to ethylene using S_2_ as an oxidant. The results reveal that surface sulfidation and facet-dependent sulfur moieties critically influence reaction pathways and activity, with S-dimer covered surfaces presenting a competitive mercaptan-mediated pathway involving formation and selective decomposition of gas phase ethyl mercaptan to ethylene. The vacancy formation energy and the average valence *p*-state electronic energy of the surface sulfur atoms are identified as descriptors for the S_2_–OEDH activity, while selectivity is broadly governed by a delicate interplay of geometric accessibility, electronic structure, and surface diffusion dynamics. Overall, precise control over sulfur species populations and surface terminations on FeS_2_ catalysts offers a plausible strategy to enhance ethylene selectivity and improve performance in sulfur-mediated oxidative dehydrogenation processes.

## Supplementary Material



## Notes

Molecular and surface geometries, adsorption and activation energies, mathematical derivations (PDF)

## References

[1] Ethylene: The “World’s Most Important Chemical”. https://www.afpm.org/newsroom/blog/ethylene-worlds-most-important-chemical (accessed 2024–08–25).

[2] Ethylene Market Size To Surpass Around USD 241.21 Billion By 2033. https://www.precedenceresearch.com/ethylene-market (accessed 2024–08–25).

[3] Sundaram, K. M. ; Shreehan, M. M. ; Olszewski, E. F. Ethylene. In Kirk-Othmer Encyclopedia of Chemical Technology; John Wiley & Sons, Ltd, 2010; pp 1–39. 10.1002/0471238961.0520082519211404.a01.pub3.

[4] Gao Y., Neal L., Ding D., Wu W., Baroi C., Gaffney A. M., Li F. (2019). Recent Advances in
Intensified Ethylene
Production—A Review. ACS Catal..

[5] Sattler J. J. H. B., Ruiz-Martinez J., Santillan-Jimenez E., Weckhuysen B. M. (2014). Catalytic Dehydrogenation of Light Alkanes on Metals
and Metal Oxides. Chem. Rev..

[6] Saito H., Sekine Y. (2020). Catalytic Conversion
of Ethane to Valuable Products
through Non-Oxidative Dehydrogenation and Dehydroaromatization. RSC Adv..

[7] Cavani F., Ballarini N., Cericola A. (2007). Oxidative Dehydrogenation of Ethane
and Propane: How Far from Commercial Implementation?. Catal. Today.

[8] Gärtner C. A., van Veen A. C., Lercher J. A. (2013). Oxidative
Dehydrogenation of Ethane:
Common Principles and Mechanistic Aspects. ChemCatChem..

[9] Najari S., Saeidi S., Concepcion P., Dionysiou D. D., Bhargava S. K., Lee A. F., Wilson K. (2021). Oxidative Dehydrogenation
of Ethane: Catalytic and Mechanistic Aspects and Future Trends. Chem. Soc. Rev..

[10] Martínez-Huerta M. V., Gao X., Tian H., Wachs I. E., Fierro J. L. G., Bañares M. A. (2006). Oxidative
Dehydrogenation of Ethane to Ethylene over Alumina-Supported Vanadium
Oxide Catalysts: Relationship between Molecular Structures and Chemical
Reactivity. Catal. Today.

[11] Argyle M. D., Chen K., Bell A. T., Iglesia E. (2002). Ethane Oxidative Dehydrogenation
Pathways on Vanadium Oxide Catalysts. J. Phys.
Chem. B.

[12] Dinse A., Schomäcker R., Bell A. T. (2009). The Role of Lattice Oxygen in the
Oxidative Dehydrogenation of Ethane on Alumina-Supported Vanadium
Oxide. Phys. Chem. Chem. Phys..

[13] Alamdari A., Karimzadeh R., Abbasizadeh S. (2021). Present State of the Art of and Outlook
on Oxidative Dehydrogenation of Ethane: Catalysts and Mechanisms. Reviews in Chemical Engineering.

[14] Jiang X., Sharma L., Fung V., Park S. J., Jones C. W., Sumpter B. G., Baltrusaitis J., Wu Z. (2021). Oxidative Dehydrogenation
of Propane to Propylene with Soft Oxidants via Heterogeneous Catalysis. ACS Catal..

[15] Kondratenko E. V., Cherian M., Baerns M. (2006). Oxidative
Dehydrogenation of Propane
over Differently Structured Vanadia-Based Catalysts in the Presence
of O2 and N2O. Catal. Today.

[16] Arinaga A. M., Ziegelski M. C., Marks T. J. (2021). Alternative Oxidants for the Catalytic
Oxidative Coupling of Methane. Angew. Chem.,
Int. Ed..

[17] Zhu Q., Wegener S. L., Xie C., Uche O., Neurock M., Marks T. J. (2013). Sulfur as a Selective ‘Soft’ Oxidant
for Catalytic Methane Conversion Probed by Experiment and Theory. Nature Chem..

[18] Peter M., Marks T. J. (2015). Platinum Metal-Free Catalysts for Selective Soft Oxidative
Methane → Ethylene Coupling. Scope and Mechanistic Observations. J. Am. Chem. Soc..

[19] Liu S., Udyavara S., Zhang C., Peter M., Lohr T. L., Dravid V. P., Neurock M., Marks T. J. (2021). “ Soft”
Oxidative Coupling of Methane to Ethylene: Mechanistic Insights from
Combined Experiment and Theory. Proc. Natl.
Acad. Sci. U.S.A..

[20] Liu S., Arinaga A. M., Lohr T. L., Marks T. J. (2020). High Ethylene-Yield
Oxidative Dehydrogenation of Ethane Using Sulfur Vapor as a “Soft”
Oxidant. ChemCatChem..

[21] Arinaga A. M., Liu S., Marks T. J. (2020). Oxidative
Dehydrogenation of Propane over Transition
Metal Sulfides Using Sulfur as an Alternative Oxidant. Catal. Sci. Technol..

[22] Arinaga A. M., Alayoglu S., Zheng D., Marks T. J. (2021). Supported Vanadium
Catalysts for Selective Sulfur-Oxidative Dehydrogenation of Propane. ChemCatChem..

[23] Arinaga A. M., Biswas A., Zhang X., Greeley J., Marks T. J. (2023). Origin
of Rate and Selectivity Trends and Exceptional Yield in Sulfur-Oxidative
Propane Dehydrogenation Over Supported Vanadium Catalysts. ACS Catal..

[24] Sulphide Catalysts, Their Properties and Applications; Elsevier, 1973. 10.1016/C2013-0-02565-0.

[25] Chianelli, R. R. ; Daage, M. ; Ledoux, M. J. Fundamental Studies of Transition-Metal Sulfide Catalytic Materials. In Adv. Catal.; Eley, D. D. , Pines, H. , Haag, W. O. , Eds.; Academic Press, 1994; Vol. 40, pp 177–232. 10.1016/S0360-0564(08)60658-6.

[26] Hung A., Muscat J., Yarovsky I., Russo S. P. (2002). Density-Functional
Theory Studies of Pyrite FeS2(1 0 0) and (1 1 0) Surfaces. Surf. Sci..

[27] Hung A., Muscat J., Yarovsky I., Russo S. P. (2002). Density-Functional
Theory Studies of Pyrite FeS2­() and () Surfaces. Surf. Sci..

[28] Alfonso D. R. (2010). Computational
Investigation of FeS2 Surfaces and Prediction of Effects of Sulfur
Environment on Stabilities. J. Phys. Chem. C.

[29] Sabbe M. K., Van Geem K. M., Reyniers M.-F., Marin G. B. (2011). First Principle-Based
Simulation of Ethane Steam Cracking. AIChE J..

[30] Trenner N.
R., Taylor H. A. (1933). The Thermal
Decomposition of Ethyl Mercaptan and Ethyl
Sulphide. J. Chem. Phys..

[31] Sehon A. H., deB. Darwent B. (1954). The Thermal
Decomposition of Mercaptans. J. Am. Chem. Soc..

[32] Boivin J. L., MacDonald R. (1955). PYROLYSIS
OF ETHYL MERCAPTAN. Can. J. Chem..

[33] Baldridge K. K., Gordon M. S., Johnson D. E. (1987). Thermal Decomposition of Methanethiol
and Ethanethiol. J. Phys. Chem..

[34] Kresse G., Furthmüller J. (1996). Efficient Iterative Schemes for Ab Initio Total-Energy
Calculations Using a Plane-Wave Basis Set. Phys.
Rev. B.

[35] Perdew J. P., Burke K., Ernzerhof M. (1996). Generalized
Gradient Approximation
Made Simple. Phys. Rev. Lett..

[36] Kresse G., Joubert D. (1999). From Ultrasoft Pseudopotentials to the Projector Augmented-Wave
Method. Phys. Rev. B.

[37] Grimme S. (2011). Density Functional
Theory with London Dispersion Corrections. WIREs
Computational Molecular Science.

[38] Grimme S., Antony J., Ehrlich S., Krieg H. (2010). A Consistent and Accurate
Ab Initio Parametrization of Density Functional Dispersion Correction
(DFT-D) for the 94 Elements H-Pu. J. Chem. Phys..

[39] Jain A., Ong S. P., Hautier G., Chen W., Richards W. D., Dacek S., Cholia S., Gunter D., Skinner D., Ceder G., Persson K. A. (2013). Commentary:
The Materials Project:
A Materials Genome Approach to Accelerating Materials Innovation. APL Materials.

[40] Monkhorst H. J., Pack J. D. (1976). Special Points for Brillouin-Zone Integrations. Phys. Rev. B.

[41] Brostigen G., Kjekshus A., Astrup E. E., Nordal V., Lindberg A. A., Craig J. C. (1969). Redetermined Crystal
Structure of FeS2 (Pyrite). Acta Chem. Scand..

[42] Toulmin P., Barton P. B. (1964). A Thermodynamic
Study of Pyrite and Pyrrhotite. Geochim. Cosmochim.
Acta.

[43] Boes J. R., Mamun O., Winther K., Bligaard T. (2019). Graph Theory
Approach
to High-Throughput Surface Adsorption Structure Generation. J. Phys. Chem. A.

[44] Reuter K., Scheffler M. (2001). Composition,
Structure, and Stability of RuO 2 (110)
as a Function of Oxygen Pressure. Phys. Rev.
B.

[45] Bollinger M. V., Jacobsen K. W., Nørskov J. K. (2003). Atomic
and Electronic Structure of
${\mathrm­{MoS}}_{2}$ Nanoparticles. Phys. Rev.
B.

[46] Henkelman G., Uberuaga B. P., Jónsson H. (2000). A Climbing
Image Nudged Elastic Band
Method for Finding Saddle Points and Minimum Energy Paths. J. Chem. Phys..

[47] Henkelman G., Jónsson H. (1999). A Dimer Method
for Finding Saddle Points on High Dimensional
Potential Surfaces Using Only First Derivatives. J. Chem. Phys..

[48] Campbell C. T., Sellers J. R. V. (2012). The Entropies
of Adsorbed Molecules. J. Am. Chem. Soc..

[49] Sprowl L.
H., Campbell C. T., Árnadóttir L. (2016). Hindered Translator
and Hindered Rotor Models for Adsorbates: Partition Functions and
Entropies. J. Phys. Chem. C.

[50] Wang V., Xu N., Liu J.-C., Tang G., Geng W.-T. (2021). VASPKIT: A User-Friendly
Interface Facilitating High-Throughput Computing and Analysis Using
VASP Code. Comput. Phys. Commun..

[51] Cao S., Tao F. (., Tang Y., Li Y., Yu J. (2016). Size- and
Shape-Dependent Catalytic Performances of Oxidation and Reduction
Reactions on Nanocatalysts. Chem. Soc. Rev..

[52] Wang C., Wang Z., Mao S., Chen Z., Wang Y. (2022). Coordination
Environment of Active Sites and Their Effect on Catalytic Performance
of Heterogeneous Catalysts. Chinese Journal
of Catalysis.

[53] Liu Y., Wang H., Yuan X., Wu Y., Wang H., Tan Y. Z., Chew J. W. (2021). Roles of Sulfur-Edge Sites, Metal-Edge
Sites, Terrace Sites, and Defects in Metal Sulfides for Photocatalysis. Chem. Catalysis.

[54] Wang J., Li G., Zhang X., Zong K., Yang Y., Zhang X., Wang X., Chen Z. (2024). Undercoordination Chemistry of Sulfur
Electrocatalyst in Lithium–Sulfur Batteries. Adv. Mater..

[55] Chu X., Liao Y., Wang L., Li J., Xu H. (2023). Engineering
Sulfur Vacancies for Boosting Electrocatalytic Reactions. Chin. Chem. Lett..

[56] Dickens C. F., Montoya J. H., Kulkarni A. R., Bajdich M., Nørskov J. K. (2019). An Electronic
Structure Descriptor for Oxygen Reactivity at Metal and Metal-Oxide
Surfaces. Surf. Sci..

[57] Xiong C., Chen S., Yang P., Zha S., Zhao Z.-J., Gong J. (2019). Structure–Performance Relationships
for Propane Dehydrogenation
over Aluminum Supported Vanadium Oxide. ACS
Catal..

[58] Zhu Y., Wang J., Patel S. B., Li C., Head A. R., Boscoboinik J. A., Zhou G. (2023). Tuning the Surface Reactivity of
Oxides by Peroxide Species. Proc. Natl. Acad.
Sci. U. S. A..

[59] Chen C.-T., Sviripa A., Verma S., Paolucci C., Flaherty D. W. (2025). Reactions
of Surface Peroxides Contribute to Rates and Selectivities for C2H4
Epoxidation on Silver. ACS Catal..

[60] Wang Y., Zhao W., Chen X., Ji Y., Zhu X., Chen X., Mei D., Shi H., Lercher J. A. (2024). Methane–H2S
Reforming Catalyzed by Carbon and Metal Sulfide Stabilized Sulfur
Dimers. J. Am. Chem. Soc..

