# Supplementary material for: Mercaptan-Mediated Ethylene Formation in Sulfur Oxidative Ethane Dehydrogenation on Iron Sulfide (FeS2) Catalysts
Source: ACS Catal. 2026 Mar 20;16(7):6278–94. doi: 10.1021/acscatal.5c07402 (PMC13054782; doi:10.1021/acscatal.5c07402)
Supplement: Supplementary file 1 [file cs5c07402_si_001.pdf]

Supporting Information for

Mercaptan-Mediated Ethylene Formation in Sulfur Oxidative Ethane Dehydrogenation on Iron Sulfide (FeS_2) Catalysts

Anik Biswas[1], *Tobin J. Marks*[2], *Jeffrey Greeley*[1,3]*

1 Davidson School of Chemical Engineering, Purdue University, West Lafayette, Indiana, 47907, USA

2 Department of Chemistry, Northwestern University, Evanston, Illinois, 60208, USA

3 Department of Chemical and Biological Engineering, University of Wisconsin-Madison, Madison, Wisconsin, 53706, USA

*jpgreeley@wisc.edu

Contents

Figure S1 Bulk pyrite (FeS_2) structure S3

Figure S2 FeS_2 bulk stability analysis S3

Grand canonical surface free energy for the additional surface sulfur structures S4

Figure S3 Side and top views of (111)-3S surface S4

Entropy and free energy estimation S5

Figure S4 FeS_2 bulk-terminated surface phase diagram S6

Table S1 S-vacancy formation energy S7

Table S2 S-adatom formation energy S7

Table S3 S-dimer formation energy S7

Table S4 S-trimer formation energy S8

Table S5 S-tetramer formation energy S8

Figure S5 S-adatom binding sites on (001)-S S9

Figure S6 S-dimer binding sites on (001)-S S9

Figure S7 S-trimer binding sites on (001)-S S9

Figure S8 S- tetramer binding sites on (001)-S S10

Figure S9 S-adatom binding sites on (210)-2S′ S10

Figure S10 S-dimer binding sites on (210)-2S′ S11

Figure S11 S-trimer binding sites on (210)-2S′ S12

Figure S12 S-tetramer binding sites on (210)-2S′ S13

Figure S13 1ML S-chain configurations on (001)-S and (210)-2S′ surfaces S14

Figure S14 Grand canonical surface energy diagram at 1 atm S_2 pressure S15

Table S6 Binding sites and adsorption energy on 1ML S-dimer covered (001)-S S16

Table S7 Binding sites and adsorption energy on 1ML S-dimer covered (210)-2S′ S18

Table S8 Binding sites and adsorption energy on (001)-S S20

Table S9 Binding sites and adsorption energy on (210)-2S′ S21

Table S10 Binding sites and adsorption energy on (111)-3S S23

Table S11 Effective activation barriers on 1ML S-dimer covered (001)-S S24

Table S12 Effective activation barriers on 1ML S-dimer covered (210)-2S′ S27

Table S13 Effective activation barriers on (001)-S S30

Table S14 Effective activation barriers on (210)-2S′ S31

Table S15 Effective activation barriers on (111)-3S S33

Figure S15 Reaction energy diagram on 1ML S-dimer covered (001)-S at 800 and 1200 K.... S34

Figure S16 Reaction energy diagram on 1ML S-dimer covered (210)-2S′ at 800 and 1200 K S35

Figure S17 Binding configurations of adsorbates on pristine (001)-S..................................... S36

Figure S18 Binding configurations of adsorbates on pristine (210)-2S′................................... S37

Figure S19 Binding configurations of adsorbates on pristine (111)-3S................................... S38

Figure S20 Initial, transition and final state geometries of MEPs on pristine (001)-S S39

Figure S21 Initial, transition and final state geometries of MEPs on pristine (210)-2S′ S40

Figure S22 Initial, transition and final state geometries of MEPs on pristine (111)-3S S41

Figure S23 Reaction energy diagram on pristine (001)-S at 800 and 1200 K S42

Figure S24 Reaction energy diagram on pristine (210)-2S′ at 800 and 1200 K S43

Figure S25 Reaction energy diagram on pristine (111)-3S at 800 and 1200 K S44

Figure S26 Correlation of H and C_2H_5 binding energy with average sulfur p-state energy S45

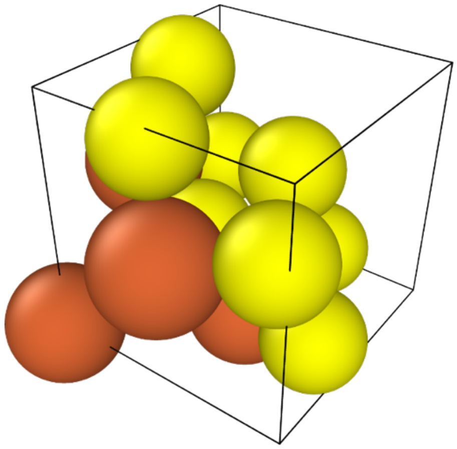

Figure S1: Bulk pyrite (FeS_2) structure. The structure is cubic with a spacegroup of Pa$\bar{3}$. The DFT-relaxed structure has a lattice constant of 5.38 Å.

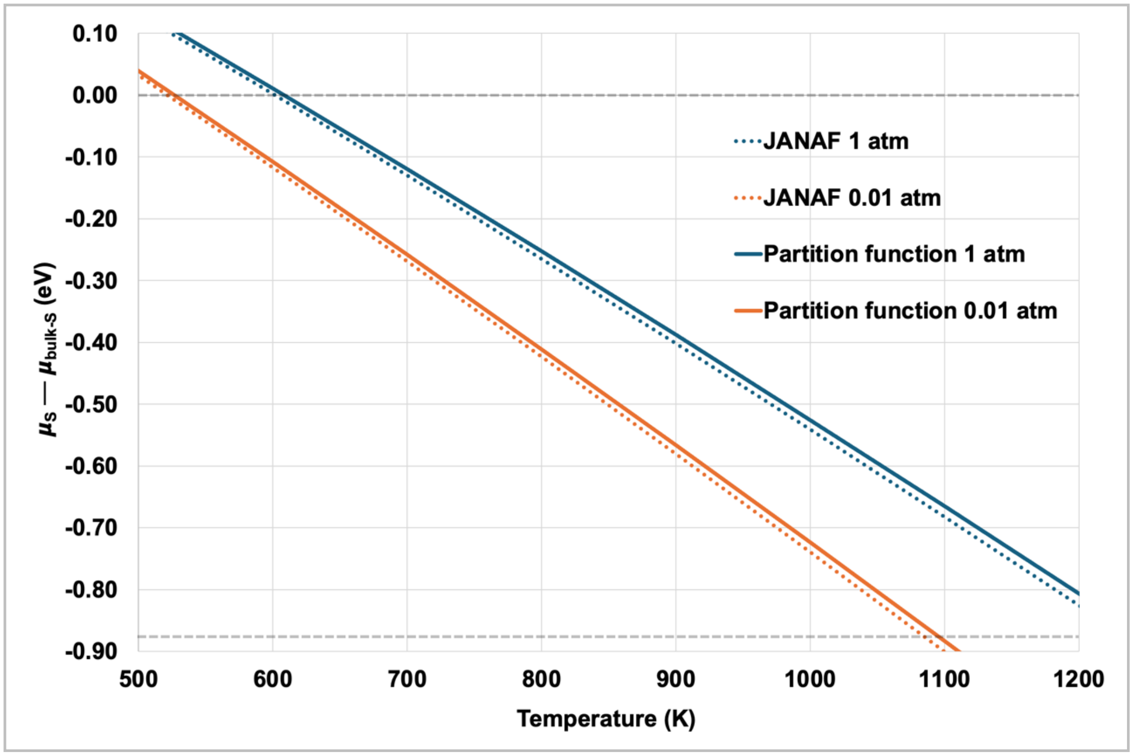

Figure S2: Grand canonical chemical potential difference ($\mu_S - \mu_{bulk\text{-}S}$) as a function of temperature for sulfur gas (S_2) at partial pressures of 1 atm (blue line) and 0.01 atm (orange line). The dashed gray horizontal lines indicate the bulk stability limits of FeS_2, defined by the condition $\Delta G_{FeS2} < \mu_S - \mu_{bulk\text{-}S} < 0$, where ΔG_{FeS2} (–0.87 eV) is the free energy of formation of FeS_2. Above the upper limit, sulfur forms solid bulk sulfur, while below the lower limit, FeS_2 decomposes into elemental Fe and S. Under the grand canonical assumption, $\mu_S = 0.5\ \mu_{S2(g)}$, which depends on temperature and pressure. Both the partition function approach and the JANAF thermochemical tables were employed to estimate μ_S.[1]

Grand canonical surface free energy for the additional surface sulfur structures:

Surface energy of a bulk-terminated pristine FeS_2 surface[2–4]:

$$\gamma(T,p) = \frac{1}{2A}\left[G^{pristine} - N_{Fe}\mu_{Fe} - N_S\mu_S\right] \qquad \text{Equation 1}$$

γ: surface free energy

G: free energy

A: area of surface slab

N: number of atoms in surface slab

Equilibrium conditions:

$$\mu_{Fe} + 2\mu_S = G^{bulk}_{FeS_2} \qquad \text{Equation 2}$$

$$2\mu_S = \mu^{gas}_{S_2} \qquad \text{Equation 3}$$

μ: chemical potential

Substituting equation 2 and 3 into equation 1:

$$\gamma(T,p) = \frac{1}{2A}\left[G^{pristine} - N_{Fe}G^{bulk}_{FeS_2} + (2N_{Fe} - N_S)\frac{\mu^{gas}_{S_2}}{2}\right] \qquad \text{Equation 4}$$

If there is any adsorption or vacancy of surface sulfur atoms, the change in surface free energy:

$$\Delta\gamma(T,p) = \frac{1}{2A}\left[G^{pristine+nS} - G^{pristine} - n\mu_S\right] \qquad \text{Equation 5}$$

n: number of adsorbed surface sulfur atoms or surface sulfur defects

$n \geq 1$ for adsorption and $n \leq -1$ for vacancies

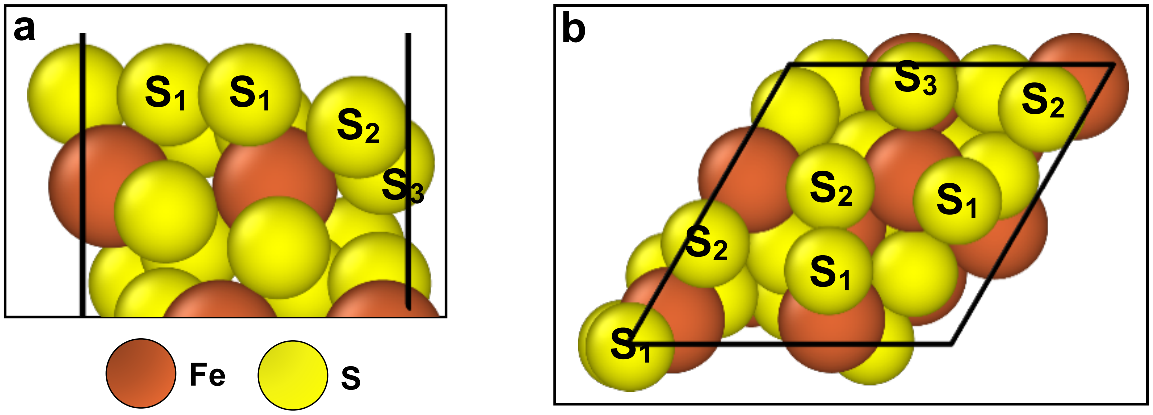

Figure S3. a) Side view and b) top view of the (111)-3S surface. Unique surface sites are labeled on each diagram.

Entropy and Free Energy Estimation

$$\mathrm{U = E_{DFT} + E_{ZPE} + k_B T^2 ln(\frac{\partial lnQ}{\partial T})_{N,V}}$$

$$\mathrm{H = U + (k_B T)_{only\ for\ 3D\ and\ 2D\ ideal\ gas}}$$

$$\mathrm{S = k_B lnQ + k_B T ln(\frac{\partial lnQ}{\partial T})_{N,V}}$$

$$\mathrm{G = H - TS}$$

$\mathrm{k_B} = Boltzmann\ Constant$

$\mathrm{Q} = Partition\ Function$

Harmonic Oscillator

$$\mathrm{Q_{harmonic}} = \frac{e^{-\frac{1}{2}\beta\hbar\omega}}{1 - e^{-\beta\hbar\omega}}$$

$\beta = \frac{1}{k_B T}$ $\hbar$ = Reduced Planck Constant ω = vibrational frequency

3D Ideal Gas

$$\mathrm{Q_{3D\ IG}} = \frac{[\left(\frac{2\pi m k_B T}{h^2}\right)^{\frac{3}{2}} V]^N}{N!}$$

m = mass V = volume of the box

2D Ideal Gas

$$\mathrm{Q_{2D\ IG}} = \frac{[\left(\frac{2\pi m k_B T}{h^2}\right) A]^N}{N!}$$

A = area of the box

2D Particle in a Box

$$\mathrm{Q_{2D\ PIB}} = \left(\frac{2\pi m k_B T}{h^2}\right) A$$

1D Ideal Gas

$$\mathrm{Q_{1D\ IG}} = \frac{[\left(\frac{2\pi m k_B T}{h^2}\right)^{\frac{1}{2}} L]^N}{N!}$$

L = length of the box

1D Particle in a Box

$$\mathrm{Q_{1D\ PIB}} = \left(\frac{2\pi m k_B T}{h^2}\right)^{\frac{1}{2}} L$$

Gas Free Rotor

$$Q_{rotor} = \frac{\sqrt{\pi}}{\sigma}\left(\frac{8\pi^2 I k_B T}{h^2}\right)^{\frac{3}{2}}$$

I = moment of intertia

Hindered Translation[5]

$$q_{trans} = \frac{M(\frac{\pi r_x}{T_x})\exp[-\frac{r_x}{T_x}]\exp[-\frac{1}{T_x}]I_0^2[\frac{r_x}{2T_x}]}{(1-\exp[-\frac{1}{T_x}])^2}\exp[\frac{2}{(2+16r_x)T_x}]$$

Hindered Rotation[5]

$$q_{rot} = \frac{(\frac{\pi r_r}{T_r})^{\frac{1}{2}}\exp[-\frac{r_r}{2T_r}]\exp[-\frac{1}{2T_r}]I_0[\frac{r_r}{2T_r}]}{(1-\exp[-\frac{1}{T_r}])}\exp[\frac{1}{(2+16r_r)T_r}]$$

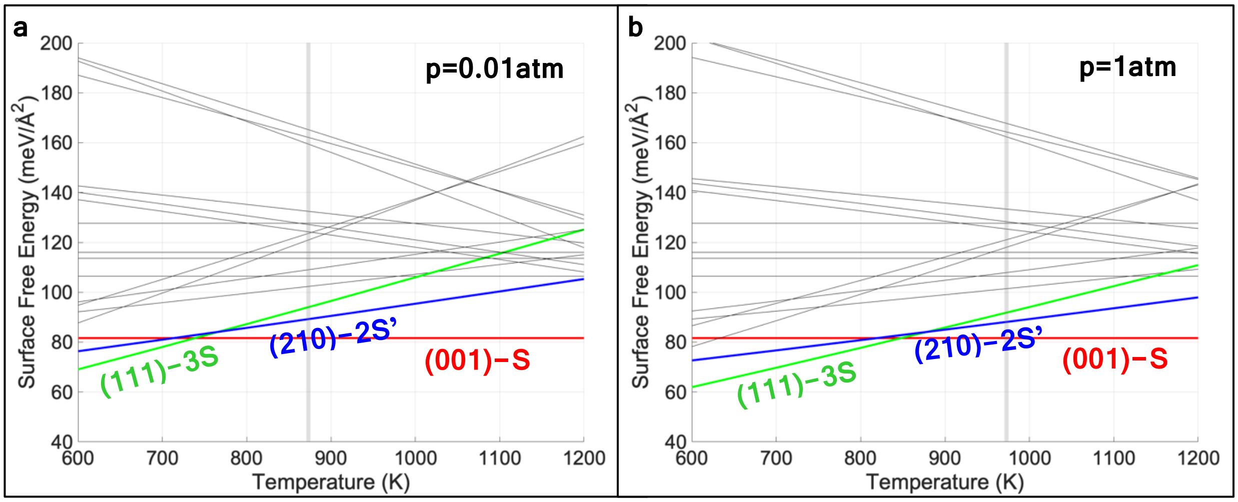

Figure S4: Grand canonical surface free energy diagram of pyrite (FeS_2), reproduced from Alfonso's work[4]. The surface free energy of several low-Miller index facets of FeS_2 is plotted as a function of temperature at gas phase sulfur partial pressures of (a) 0.01 atm and (b) 1 atm. The (001)-S, (210)-2S′, and (111)-3S surface facets are represented by red, blue, and green lines, respectively. Other higher energy surface facets are shown in black. The vertical line indicates the equilibrium boundary between bulk phases of pyrite and pyrrhotite, a non-stoichiometric iron sulfide compound with a lower sulfur content than pyrite.

Table S1. S-vacancy formation energy of surface sulfur atoms on the (001)-S, (210)-2S′, and (111)-3S surfaces

001-S *(coverage 0.125ML)*		***210-2S'*** *(coverage 0.25ML)*		***111-3S*** *(coverage 0.33ML)*	
**Site**	**ΔE_{vac} (eV)**	**Site**	**ΔE_{vac} (eV)**	**Site**	**ΔE_{vac} (eV)**
S	2.98	S_{1A}	1.92	S_1	0.94
		S_{1B}	2.47	S_2	2.19
		S_{2A}	2.31	S_3	2.64
		S_{2B}	2.32		

$$\Delta E_{vac} = E_{slab\ with\ surface\ S\ removed} - E_{pristine\ slab} + \frac{1}{2}E_{S_2(g)} \quad (1)$$

Table S2. S-adatom ("mono") formation energies on the (001)-S and (210)-2S′ surface sites

001-S *(coverage: 0.125ML)*		***001-S*** *(coverage: 1ML)*		***210-2S'*** *(coverage: 1ML)*	
**Site**	**ΔE_{mono} (eV)**	**Site**	**ΔE_{mono} (eV)**	**Site**	**ΔE_{mono} (eV)**
S top	-0.36	S top	-0.28	S_{1A} top	-0.68
Fe-S bridge	-0.13				
Fe top	-0.36	Fe top	-0.58		
210-2S' *(coverage: 0.25ML)*					
**Site**	**ΔE_{mono} (eV)**	**Site**	**ΔE_{mono} (eV)**	**Site**	**ΔE_{mono} (eV)**
S_{1A} top	-0.68	S_{1A}-Fe-bridge	-0.65	S_{2A}-Fe bridge	-0.10
S_{1B} top	-0.14	S_{1A}-Fe-bridge	-0.21	S_{1A}-S_{2A} bridge	-0.08
S_{2A} top	-0.05	S_{1B}-Fe bridge	-0.14	S_{1A}-S_{2B} bridge	-0.52

$$\Delta E_{mono} = \frac{1}{n}\left[E_{slab\ with\ n\ S\ adatoms} - E_{pristine\ slab} - \frac{n}{2}E_{S_2(g)}\right] \quad (2)$$

Table S3. S-dimer formation energies on the (001)-S and (210)-2S′ surface sites

(001)-S *(coverage: 0.25ML)*				***(001)-S*** *(coverage: 1ML)*	
**Site**	**ΔE_{dimer} (eV)**	**Site**	**ΔE_{dimer} (eV)**	**Site**	**ΔE_{dimer} (eV)**
Fe-Fe	-2.05	Fe-S	-1.58	Fe-Fe	-1.97
Fe-S	-1.21	Fe-S	-1.23		
Fe-S	-1.14				
(210)-2S' *(coverage: 0.25ML)*				***(210)-2S'*** *(coverage: 1ML)*	
**Site**	**ΔE_{dimer} (eV)**	**Site**	**ΔE_{dimer} (eV)**	**Site**	**ΔE_{dimer} (eV)**
Fe-S_{1A}	-1.72	S_{1A}-S_{2B}	-0.83	Fe-S_{1A}	-1.77
Fe-S_{1B}	-0.93	S_{1A}	-1.05		
Fe-S_{2A}	-0.99	S_{1A}	-0.89		
S_{1A}-S_{1B}	-1.01	S_{1B}-S_{2A}	-0.67		
S_{1A}-S_{2A}	-1.03				

$$\Delta E_{dimer} = \frac{1}{n}\left[E_{slab\ with\ n\ S\ dimers} - E_{pristine\ slab} - nE_{S_2(g)}\right] \quad (3)$$

Table S4. S-trimer formation energies on the (001)-S and (210)-2S′ surface sites

(001)-S *(coverage: 0.25ML)*				***(001)-S*** *(coverage: 1ML)*	
**Site**	**ΔE_{trimer} (eV)**	**Site**	**ΔE_{trimer} (eV)**	**Site**	**ΔE_{trimer} (eV)**
Fe-Fe	-2.92	Fe-S	-1.99	Fe-Fe	-2.91
Fe-Fe	-2.13	Fe-S	-2.09		
(210)-2S' *(coverage: 0.25ML)*				***(210)-2S'*** *(coverage: 1ML)*	
**Site**	**ΔE_{trimer} (eV)**	**Site**	**ΔE_{trimer} (eV)**	**Site**	**ΔE_{trimer} (eV)**
S_{1A}-S_{1B}	-1.57	S_{1A}-Fe	-2.23	S_{1A}-Fe	-2.60
S_{1A}-S_{1B}	-1.47	S_{1B}-Fe	-1.90		
S_{1A}-Fe	-2.52				

$$E_{trimer} = \frac{1}{n}[E_{slab\ with\ \ n\ S\ trimers} - E_{pristine\ slab} - \frac{3n}{2}E_{S_2(g)}] \quad (4)$$

Table S5. S-tetramer formation energies on the (001)-S and (210)-2S′ surface sites

(001)-S *(coverage: 0.25ML)*				***(001)-S*** *(coverage: 1ML)*	
**Site**	**$\Delta E_{tetramer}$ (eV)**	**Site**	**$\Delta E_{tetramer}$ (eV)**	**Site**	**$\Delta E_{tetramer}$ (eV)**
Fe-Fe	-2.91	Fe-S	-2.34	Fe-Fe	-3.19
Fe-Fe	-3.23	Fe-S	-2.62	Fe-Fe	-3.46
S-chain (0.5ML)	-3.21			S-chain	-3.16
(210)-2S' *(coverage: 0.25ML)*					
**Site**	**$\Delta E_{tetramer}$ (eV)**	**Site**	**$\Delta E_{tetramer}$ (eV)**	**Site**	**$\Delta E_{tetramer}$ (eV)**
S_{1A}-Fe	-2.91	S_{1A}-Fe	-2.80	S_{1A}-S_{1B}	-2.63
S_{1A}-Fe	-2.86	S_{1A}-Fe	-2.95	S_{1A}-S_{2B}	-2.09
S_{1A}-Fe	-2.78	S_{1A}-Fe	-2.54	Fe-Fe	-2.71
S_{1A}-Fe	-2.70	S_{1B}-Fe	-2.57	S-chain (0.5 ML)	-2.55
S_{1A}-Fe	-2.72	S_{1A}-S_{1B}	-2.32		
(210)-2S' *(coverage: 1ML)*					
**Site**	**$\Delta E_{tetramer}$ (eV)**	**Site**	**$\Delta E_{tetramer}$ (eV)**	**Site**	**$\Delta E_{tetramer}$ (eV)**
S_{1A}-Fe	-3.06	S_{1A}-Fe	-2.82	S-chain	-2.62
S_{1A}-Fe	-2.95	S_{1A}-Fe	-2.96		
S_{1A}-Fe	-2.95	S_{1B}-Fe	-2.66		
S_{1A}-Fe	-2.86	S_{1A}-S_{1B}	-2.20		
S_{1A}-Fe	-2.98	S_{1A}-Fe-S_{1B}	-2.61		

$$\Delta E_{tetramer} = \frac{1}{n}[E_{slab\ with\ \ n\ S\ trimers} - E_{pristine\ slab} - 2nE_{S_2(g)}] \quad (5)$$

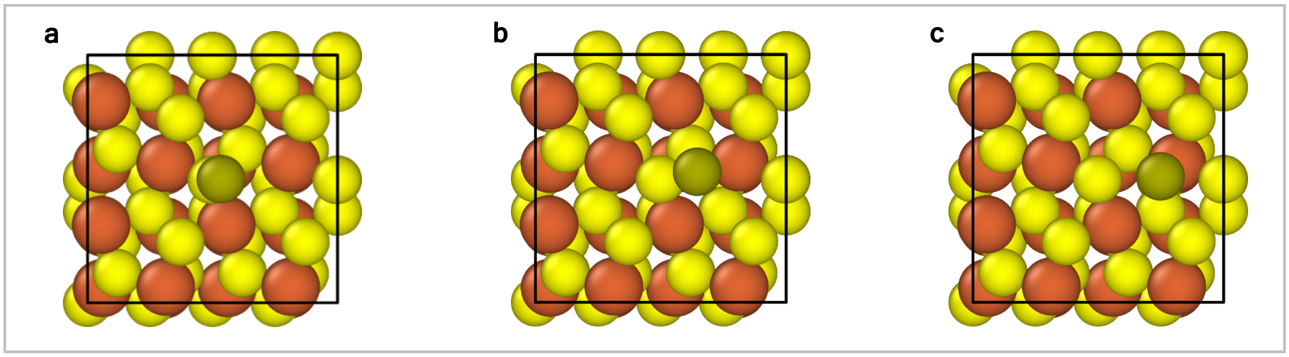

Figure S5: Top views of the S-adatom adsorption sites on the pristine (001)-S surface at a 0.125 ML coverage on a) S top, b) Fe-S bridge, and c) Fe top sites

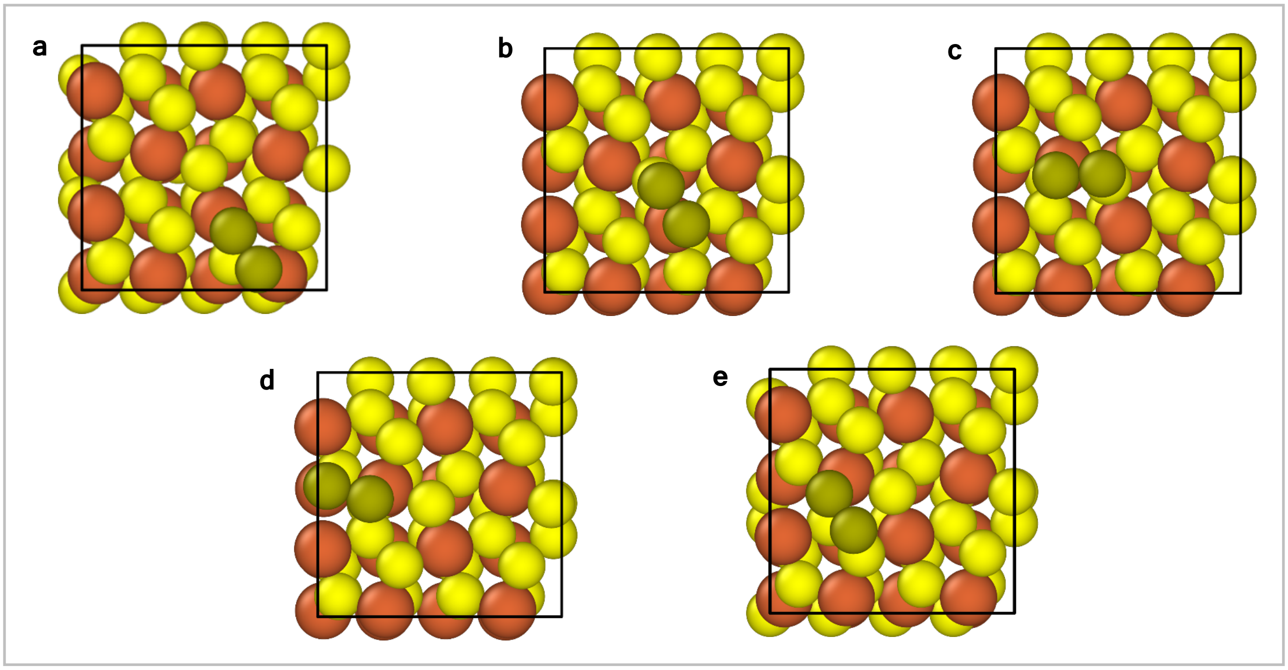

Figure S6: Top views of the S-dimer adsorption sites on the pristine (001)-S surface at a 0.25 ML coverage on a) Fe-Fe site, and b-e) on Fe-S sites

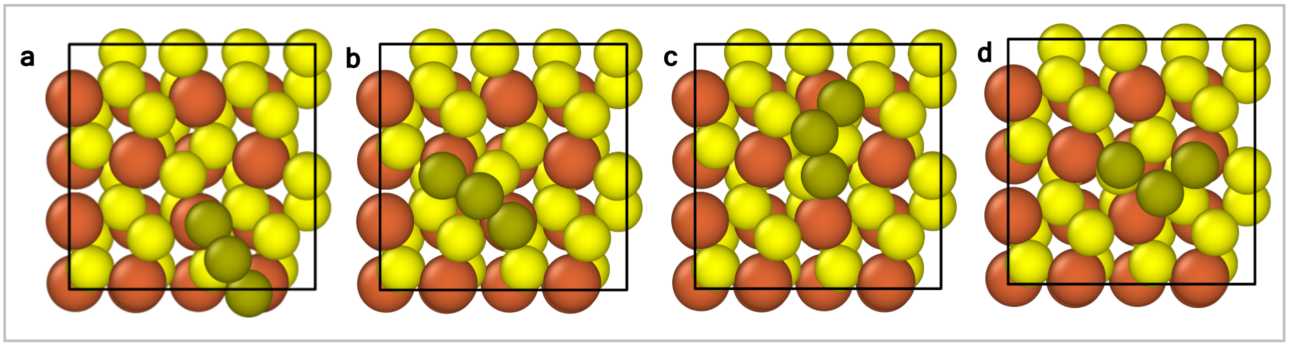

Figure S7: Top views of the S-trimer adsorption sites on the pristine (001)-S surface at a 0.25 ML coverage on a-b) Fe-Fe, and c-d) Fe-S sites

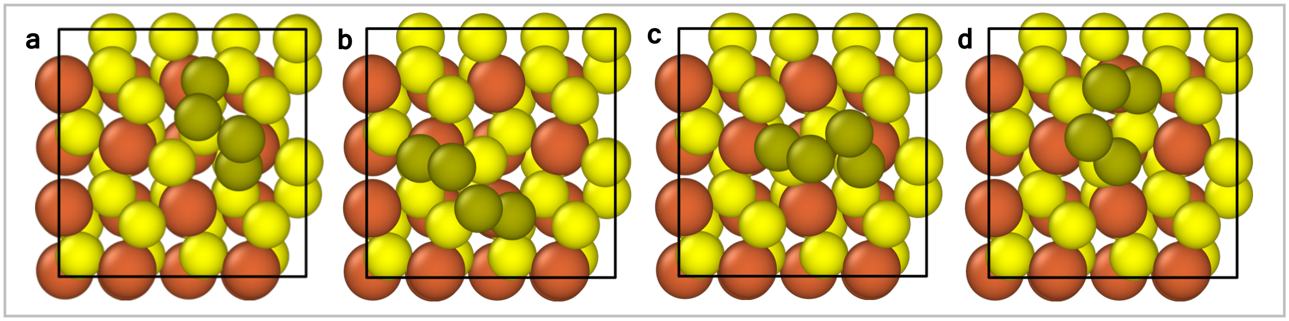

Figure S8: Top views of the S-tetramer adsorption sites on the pristine (001)-S surface at a 0.25 ML coverage on a-b) Fe-Fe, and c-d) Fe-S sites

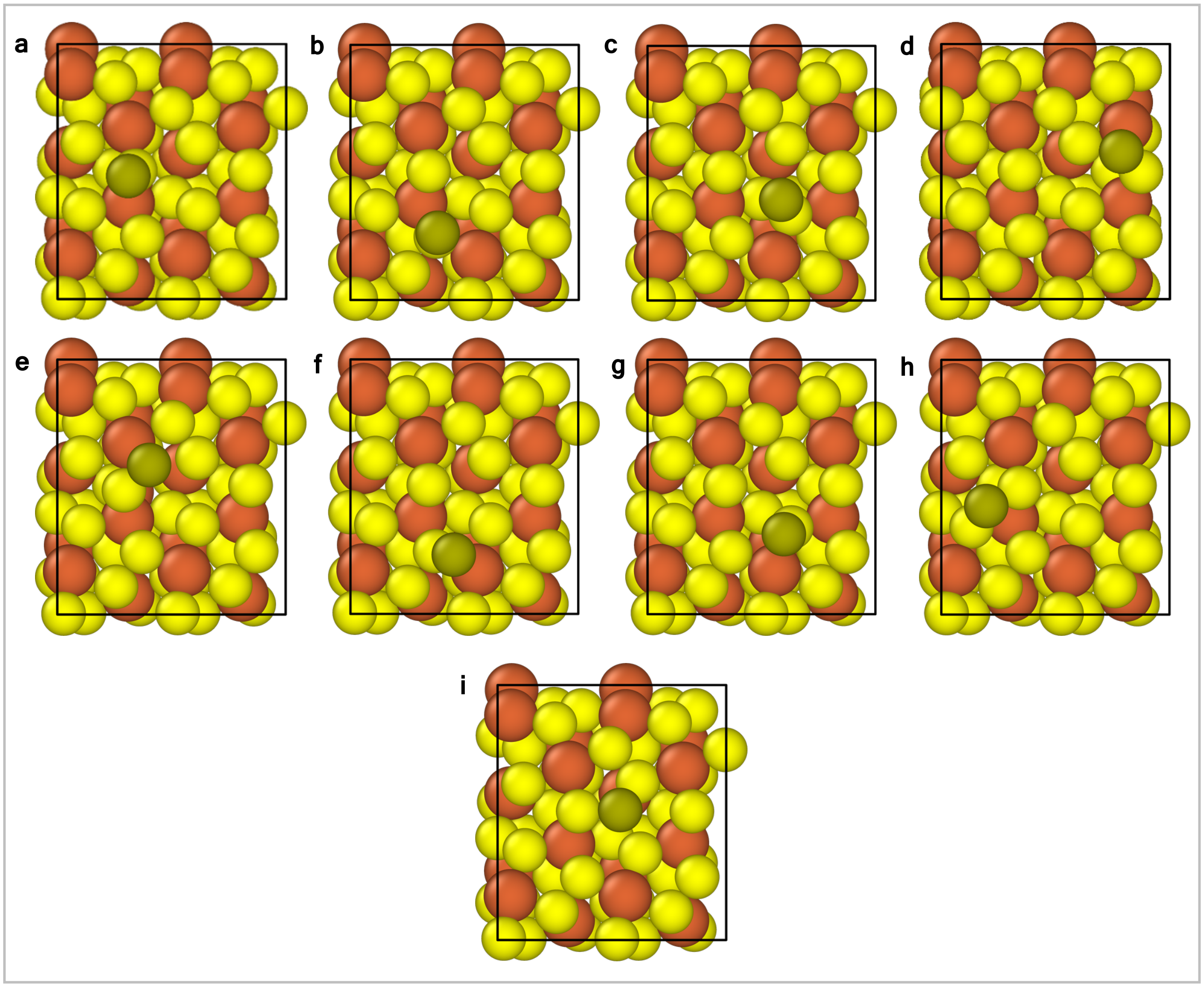

Figure S9: Top views of the S-adatom adsorption sites on the pristine (210)-2S′ surface at a 0.25 ML coverage on a) S_{1A} top, b) S_{1B} top, c) S_{2A} top, d-e) S_{1A}-Fe-bridge, f) S_{1B}-Fe bridge, g) S_{2A}-Fe bridge, h) $S_{1A\text{-}2A}$ bridge, and i) $S_{1A\text{-}2B}$ bridge sites

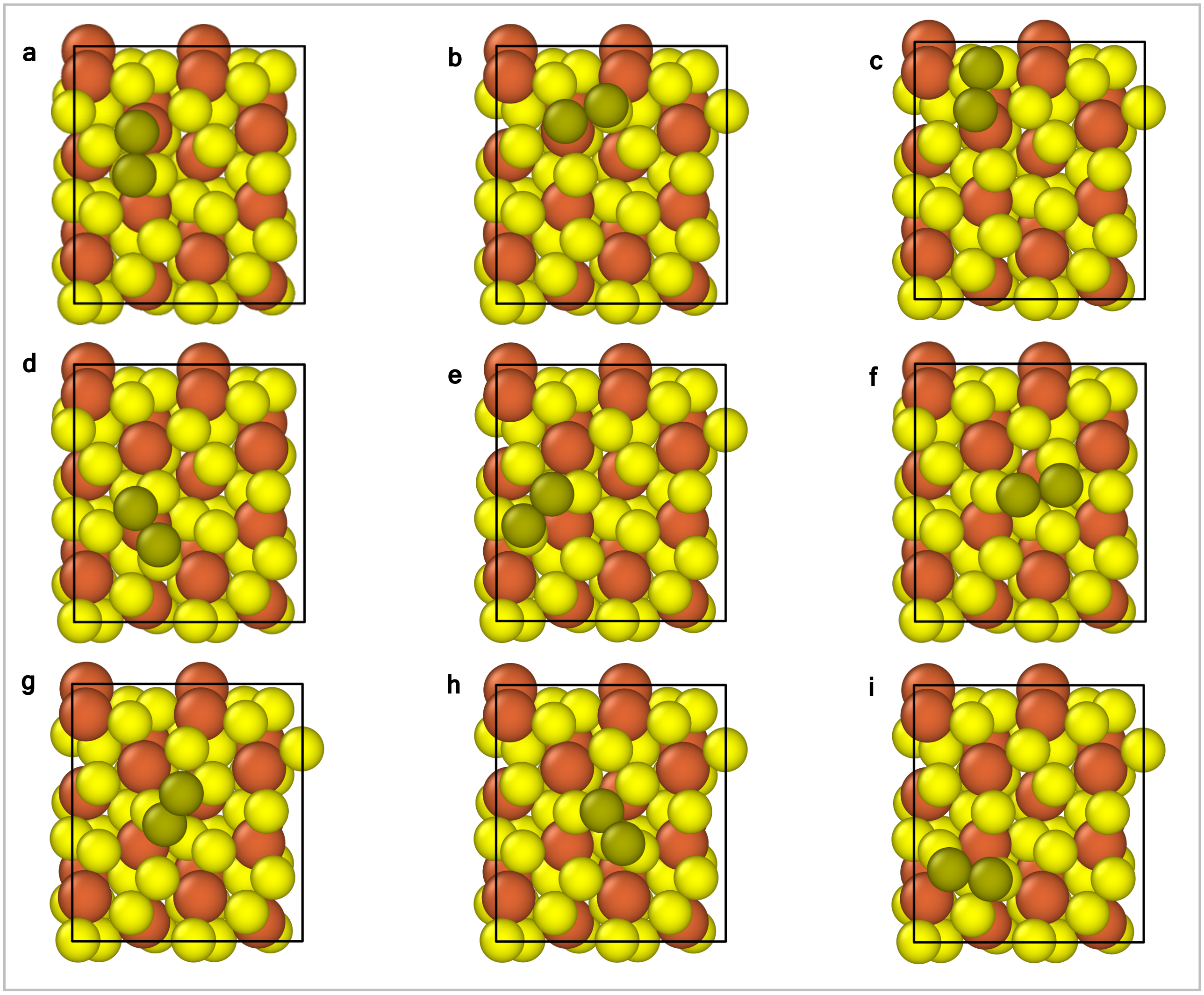

Figure S10: Top views of the S-dimer adsorption sites on the pristine (210)-2S′ surface at a 0.25 ML coverage on a) Fe-S_{1A}, b) Fe-S_{1B}, c) Fe-S_{2A}, d) S_{1A}-S_{1B}, e) S_{1A}-S_{2A}, f) S_{1A}-S_{2B}, g-h) S_{1A}, and i) S_{1B}-S_{2A} sites

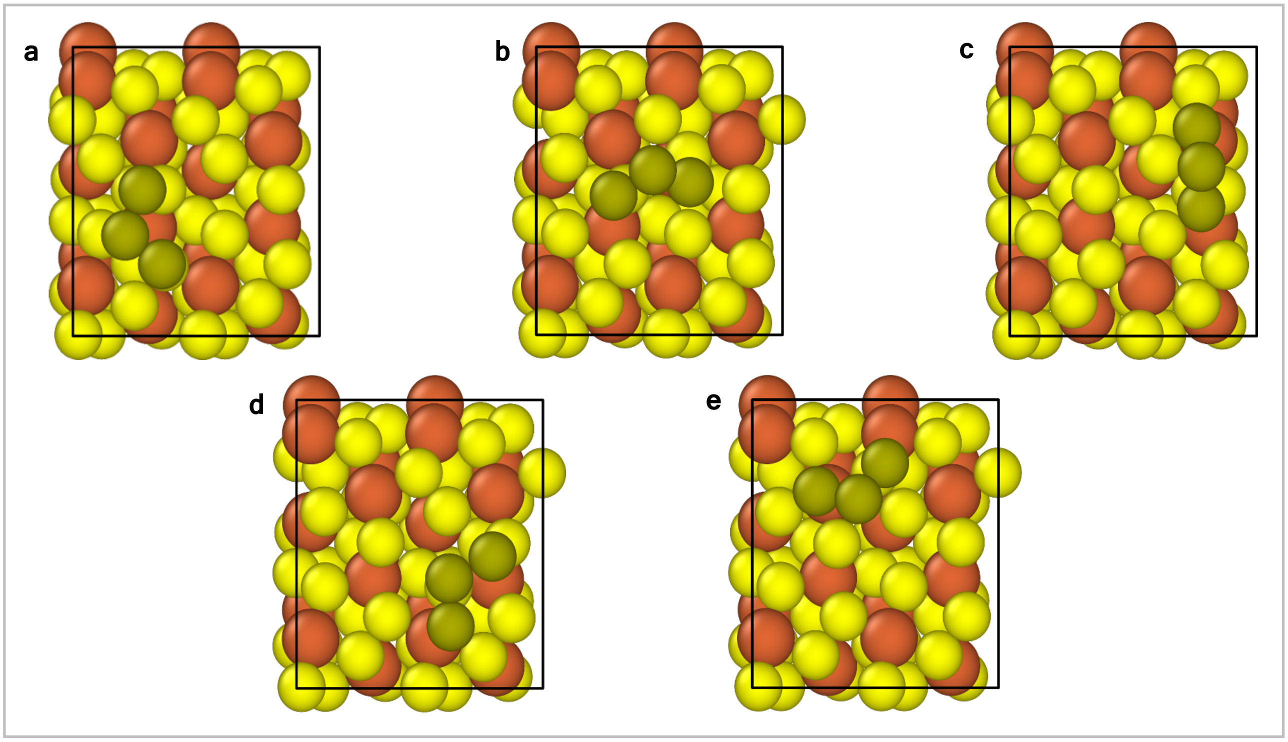

Figure S11: Top views of the S-trimer adsorption sites on the pristine (210)-2S′ surface at a 0.25 ML coverage on a) S_{1A}-S_{1B}, b) S_{1A}-S_{2B}, c-d) S_{1A}-Fe, and e) S_{1B}-Fe sites

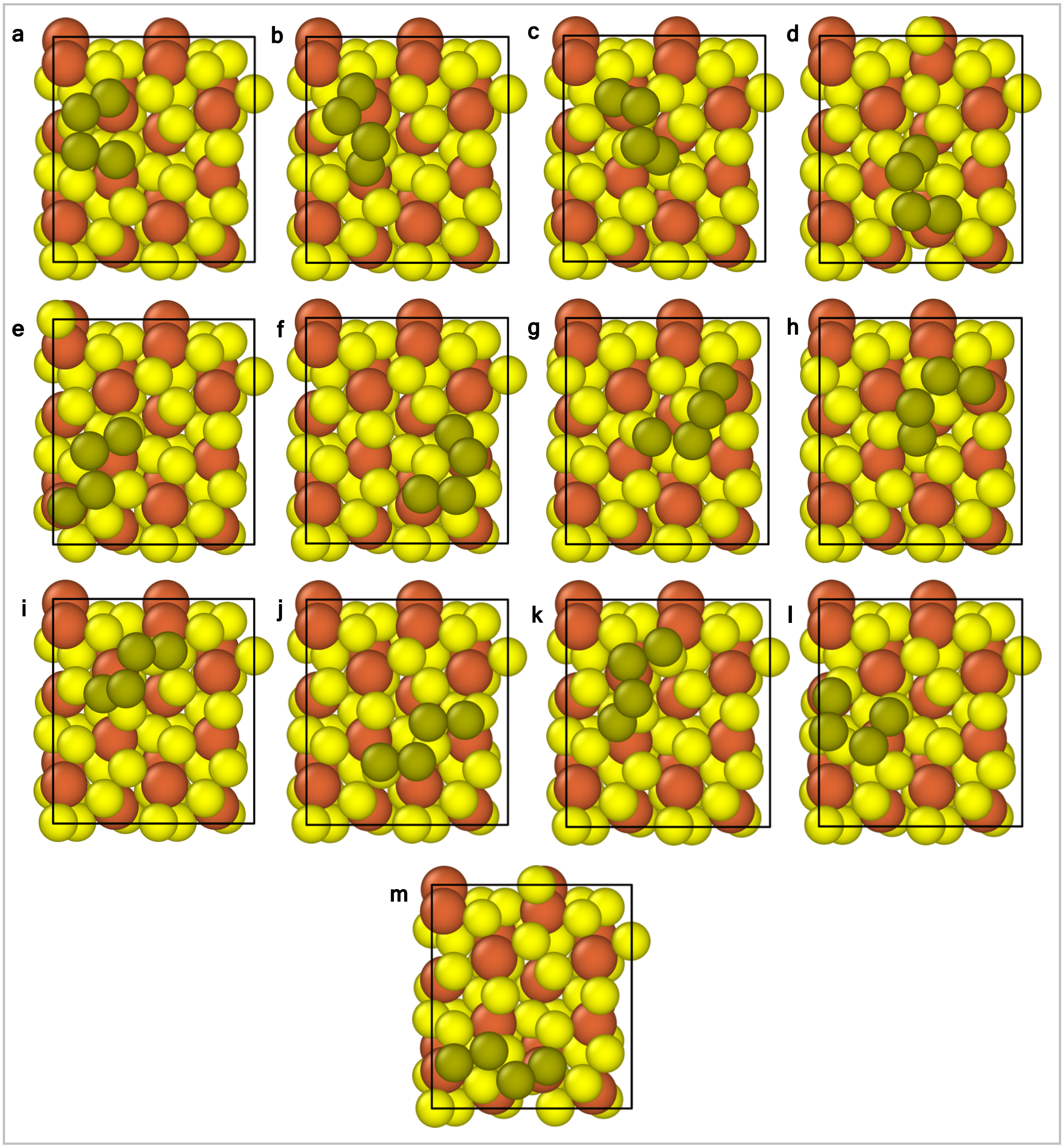

Figure S12: Top views of the S-tetramer adsorption sites on the pristine (210)-2S′ surface at a 0.25 ML coverage on a-h) S_{1A}-Fe sites, i) S_{1B}-Fe, j-k) S_{1A}-S_{1B}, l) S_{1A}-S_{2B}, and m) Fe-Fe sites

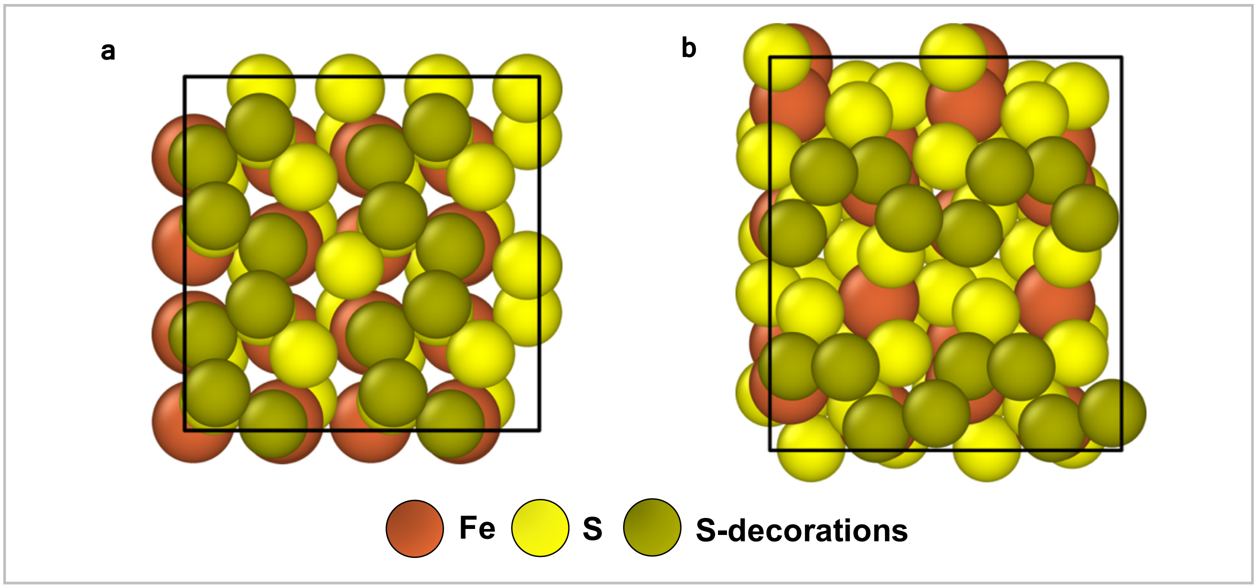

Figure S13. Top views of 1ML S-chain at Fe-Fe sites on the pristine a) (001)-S facet and b) (210)-2S′ facet.

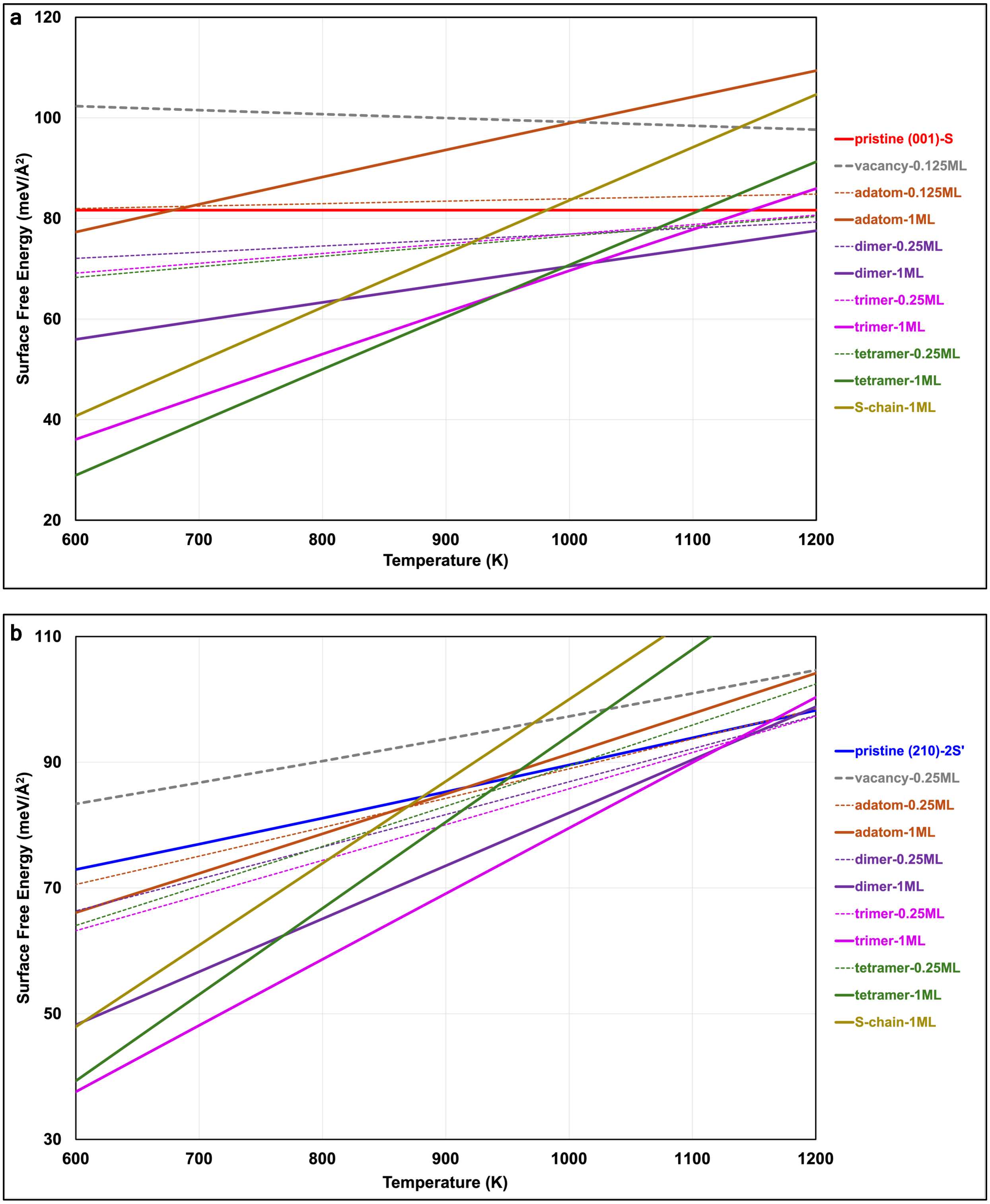

Figure S14: Surface free energy diagram of S-decorated structures at a gas phase S_2 partial pressure of 1 atm on the (001)-S and (210)-2S′ surfaces

Table S6. Binding Sites and Energy of Adsorbates on 1 ML S-dimer covered (001)-S

Adsorbate	Binding Site	Binding Energy (eV)	
H	dim_1 top	**-0.24**	
	dim_1 top	**-0.23**	
	dim_2 top	-0.14	
	dim_2 top	-0.14	
	S_1 top	-0.02	
	S_2 top	0.02	
CH_3CH_2		***S-S bond intact***	***S-S bond cleaved***
	dim_1 top	**-0.02**	0.91
	dim_1 top	0.30	1.01
	dim_2 top	0.06	**0.70**
	dim_2 top	0.39	0.78
	S_1 top	1.03	-
	S_1 top	1.27	-
	S_2 top	0.89	-
	S_2 top	0.89	-
	S_2 top	1.06	-
CH_2CH_2	dim_1-dim_2	1.05	
	dim_1-dim_2 (same dimer)	1.19	
	dim_1-dim_2 (same dimer)	1.06	
	dim_1-S_1	1.18	
	dim_1-S_1	1.15	
	dim_2-S_2	1.16	
	dim_2-S_2	**0.99**	
	S_1-S_2	1.91	
	S_1-S_2	2.61	
	dim_1 button	1.94	
	dim_2 button	1.92	
	physiosorbed	1.45	
CH_3CH	dim_1 top	1.58	
	dim_1 top	1.60	
	dim_2 top	1.47	
	dim_2 top	1.55	
	dim_1-S_1 bridge	**0.91**	
	dim_1-S_1 bridge	1.26	
	dim_1-S_1 bridge	1.45	
	dim_1-S_1 bridge	1.29	
	dim_2-S_2 bridge	**0.92**	
	dim_2-S_2 bridge	1.12	
	dim_2-S_2 bridge	1.99	
	dim_2-S_2 bridge	1.98	
	dim_1-S_2 bridge	1.16	
	dim_2-S_1 bridge	1.09	

	S_1-S_2 bridge	2.74	
	S_1-S_2 bridge	2.29	
CH_2CH	dim_1 top	1.72	
	dim_1 top	1.95	
	dim_2 top	1.82	
	dim_2 top	2.05	
	dim_1-dim_1.S_1	1.42	
	dim_1-dim_2.S_1	1.98	
	dim_1-dim_2.S_2	1.42	
	dim_2-dim_1.S_2	1.94	
	dim_2-dim_1.S_1	**1.28**	
	dim_2-dim_2.S_2	1.71	
	S_1 top	2.58	
	S_1 top	2.73	
	S_2 top	2.46	
	S_2 top	2.45	
	S_1-S_2.dim_2	2.70	
	S_2-S_1.dim_2	2.56	
	dim_1-S_1	2.76	
	dim_1-S_1	1.61	
	dim_1-S_2	2.43	
	dim_1-S_2	2.14	
	dim_2-S_1	1.77	
	dim_2-S_2	2.40	
	dim_2-S_2	2.44	
	S_1-dim_1	1.97	
	S_2-dim_2	2.46	
	S_2.S_1-dim_1	2.42	
	S_2.S_1-dim_1	2.37	
	S_1-S_2	2.81	
		S-S bond intact	
CH_3CH_2SH	dim_1	0.42	***S-S bond cleaved***
	dim_1	0.56	0.12
	dim_2	**0.34**	0.30
	dim_2	0.50	**-0.05**
		S-S bond intact	0.04
CH_3CH_2+H	dim_1 + dim_2	**-0.30**	
	dim_1 + dim_2	0.02	
	dim_2 + dim_1	**-0.31**	
	dim_2 + dim_2	0.10	

The equations used to calculate binding energies are mentioned in the main paper in section 3.2.

Note: A single site can accommodate multiple binding configurations of the same adsorbate, depending on the orientation of the adsorbate.

Gray shades represent similar binding site geometry groupings.

Table S7. Binding Sites and Energy of Adsorbates on 1 ML S-dimer covered (210)-2S′

Adsorbate	Binding Site	Binding Energy (eV)	
H	dim$_1$ top	**-0.17**	
	dim$_1$ top	**-0.14**	
	dim$_2$ top	0.03	
	dim$_2$ top	-0.01	
	S$_{1A}$ top	0.09	
	S$_{1B}$ top	0.07	
	S$_{2A}$ top	0.01	
	S$_{2B}$ top	-0.09	
CH_3CH_2		***S-S bond intact***	***S-S bond cleaved***
	dim$_1$ top	**0.04**	0.58
	dim$_1$ top	0.36	**0.52**
	dim$_2$ top	0.24	0.89
	dim$_2$ top	0.43	0.94
	S$_{1B}$ top	0.76	-
	S$_{1B}$ top	0.83	-
	S$_{2A}$ top	1.79	-
	S$_{2A}$ top	1.60	-
CH_2CH_2	dim$_1$-S$_{1B}$	**1.02**	
	dim$_1$-S$_{1B}$	1.41	
	dim$_1$-S$_{2B}$	1.45	
	dim$_2$-S$_{1B}$	1.76	
	dim$_2$-S$_{2A}$	2.06	
	dim$_2$-S$_{2B}$	2.08	
	physiosorbed	1.52	
	S$_{1B}$-S$_{2A}$	3.05	
	S$_{1B}$-S$_{2A}$	2.52	
CH_3CH	dim$_1$ top	1.95	
	dim$_2$ top	1.59	
	dim$_1$-S$_{1B}$ bridge	1.65	
	dim$_1$-S$_{1B}$ bridge	1.69	
	dim$_1$-S$_{1B}$ bridge	1.47	
	dim$_1$-S$_{1B}$ bridge	1.55	
	dim$_1$-S$_{2B}$ bridge	2.04	
	dim$_1$-S$_{2B}$ bridge	1.86	
	dim$_2$-S$_{1A}$ bridge	0.87	
	dim$_2$-S$_{1A}$ bridge	**0.50**	
	dim$_2$-S$_{2A}$ bridge	2.27	
	dim$_2$-S$_{2A}$ bridge	2.09	
	S$_{2A}$-Fe bridge	2.61	
	S$_{2A}$-Fe bridge	2.73	
	S$_{1A}$-S$_{2A}$ bridge	2.39	
	S$_{1A}$-S$_{2A}$ bridge	2.52	

	S_{1A}-S_{2B} bridge	2.43	
	S_{1A}-S_{2B} bridge	2.29	
CH_2CH	dim_1 top	1.95	
	dim_1 top	1.81	
	dim_2 top	2.03	
	dim_2 top	2.00	
	dim_1-dim_2	**1.44**	
	dim_2-dim_1	1.52	
	dim_1-dim_1.S_{1B}	1.97	
	dim_2-dim_1.S_{1B}	2.64	
	dim_1-S_{2B}.S_{1A}	2.67	
	dim_1-S_{2B}.S_{2A}	2.91	
	S_{1B}-S_{2A}	4.10	
	S_{1B}-S_{2A}	3.16	
	S_{2A}-S_{1B}	3.96	
	S_{2A}-S_{1B}	3.65	
	S_{1B} top	2.47	
	S_{1B} top	2.43	
CH_3CH_2SH		***S-S bond intact***	***S-S bond cleaved***
	dim_1	0.84	-0.21
	dim_1	1.05	**-0.25**
	dim_2	0.51	0.77
	dim_2	**0.47**	0.79
CH_3CH_2+H	***S-S bond intact***		
	dim_1 + dim_2	-0.04	
	dim_1 + dim_2	**-0.08**	
	dim_2 + dim_1	0.18	
	dim_2 + dim_2	0.04	

The equations used to calculate binding energies are mentioned in the main paper in section 3.2.

Note: A single site can accommodate multiple binding configurations of the same adsorbate, depending on the orientation of the adsorbate.

Gray shades represent similar binding site geometry groupings.

Table S8. Binding Sites and Energy of Adsorbates on the Pristine (001)-S Surface

Adsorbate	Binding Site	Binding Energy (eV)
H	Fe top	**0.36**
	S top	0.61
CH_3CH_2	Fe top	0.90
	Fe top	0.99
	Fe top	0.98
	S top	**0.71**
	S top	0.77
	S top	0.80
CH_2CH_2	Fe-S close	1.81
	Fe-S close	1.75
	Fe-S far	1.81
	Fe-S far	1.52
	Fe (button)	**1.19**
	S (button)	2.65
	Fe-Fe bidentate	2.33
	S-S bidentate	2.05
	physiosorbed	1.32
CH_3CH	Fe top	**1.66**
	Fe top	1.79
	S top	1.85
	S top	2.01
	S-Fe bridge	2.24
	S-Fe bridge	2.21
	S-S bridge	2.14
	S-S bridge	2.11
CH_3CH_2SH	S site	**1.38**
		1.43

The equations used to calculate binding energies are mentioned in the main paper in section 3.2.

Note: A single site can accommodate multiple binding configurations of the same adsorbate, depending on the orientation of the adsorbate.

Gray shades represent similar binding site geometry groupings.

Table S9. Binding Sites and Energy of Adsorbates on the Pristine (210)-2S′ Surface

Adsorbate	Binding Site	Binding Energy (eV)
H	Fe top	0.44
	S_{1A} top	-0.44
	S_{1A} top	**-0.52**
	S_{1B} top	0.24
	S_{2A} top	0.04
	S_{2B} top	0.07
CH_3CH_2	Fe top	0.92
	Fe top	0.96
	Fe top	0.97
	S_{1A} top	**-0.06**
	S_{1A} top	0.00
	S_{1A} top	0.08
	S_{1B} top	0.33
	S_{1B} top	0.34
	S_{1B} top	0.34
	S_{2A} top	0.62
	S_{2A} top	0.89
	S_{2A} top	0.95
	S_{2B} top	1.13
	S_{2B} top	0.77
	S_{2B} top	1.14
CH_2CH_2	Fe button	1.34
	Fe-S_{1A}	1.40
	Fe-S_{1B}	1.98
	Fe-S_{2A}	1.97
	Fe-S_{2B}	2.26
	S_{1A}-S_{1B}	**1.11**
	S_{1A}-S_{1B}	**1.16**
	S_{2A}-S_{2B}	1.89
	S_{1A}-S_{2A}	2.49
	S_{1A}-S_{2A}	**1.14**
	S_{1A}-S_{2B}	1.36
	S_{1A}-S_{2B}	**1.15**
	S_{1B}-S_{2A}	1.82
	S_{1B}-S_{2A}	2.06
	S_{1B}-S_{2B}	2.06

Adsorbate	Binding Site	Binding Energy (eV)
CH_3CH	Fe top	2.00
	Fe top	1.98
	Fe top	2.10
	S_{1A} top	1.42
	S_{1A} top	1.26
	S_{1A} top	1.42
	S_{1A} top	**1.05**
	S_{1B} top	2.07
	S_{1B} top	2.16
	S_{1B} top	2.17
	S_{2A} top	2.13
	S_{2A} top	2.19
	S_{2A} top	2.43
	S_{2B} top	2.61
	S_{2B} top	2.81
	S_{2B} top	2.62
	S_{1A}-S_{1B} bridge	1.81
	S_{1A}-S_{1B} bridge	1.79
	S_{2A}-S_{2B} bridge	1.92
	S_{2A}-S_{2B} bridge	2.37
	S_{1A}-S_{2A} bridge	1.70
	S_{1A}-S_{2A} bridge	1.70
	S_{1A}-S_{2A} bridge	1.58
	S_{1A}-S_{2A} bridge	1.51
	S_{1A}-S_{2B} bridge	1.14
	S_{1A}-S_{2B} bridge	1.47
	S_{1A}-S_{2B} bridge	1.38
	S_{1A}-S_{2B} bridge	1.85
	S_{1B}-S_{2A} bridge	1.74
	S_{1B}-S_{2A} bridge	1.83
	S_{1B}-S_{2A} bridge	2.02
	S_{1B}-S_{2A} bridge	2.50
	S_{1B}-S_{2B} bridge	2.08
	S_{1B}-S_{2B} bridge	2.30
CH_3CH_2SH	S_{1A} site	**-0.37**
		-0.29
		-0.21

The equations used to calculate binding energies are mentioned in the main paper in section 3.2.

Note: A single site can accommodate multiple binding configurations of the same adsorbate, depending on the orientation of the adsorbate.

Gray shades represent similar binding site geometry groupings.

Table S10. Binding Sites and Energy of Adsorbates on the Pristine (111)-3S Surface

Adsorbate	Binding Site	Binding Energy (eV)
H	Fe top	0.54
	S_1 top	-0.52
	S_1 top	-0.44
	S_2 top	-0.17
	S_3 top	-0.61
CH_3CH_2	S_1 top	0.25
	S_1 top	0.26
	S_1 top	0.11
	S_1 top	0.11
	S_1 top	0.34
	S_2 top	0.36
	S_2 top	0.55
	S_2 top	0.52
	S_3 top	0.46
CH_2CH_2	S_1 button	1.76
	S_2 button	3.28
	S_1-S_1	1.15
	S_1-S_2	1.74
	S_1-S_2	1.37
	S_1-S_2	0.79
	S_1-S_3	0.81
	S_2-S_2	1.98
	S_2-S_3	1.20
	physiosorbed	1.58
CH_3CH	S_1 top	1.44
	S_1 top	1.26
	S_1-S_1 bridge	0.74
	S_1-S_1 bridge	1.08
	S_1-S_2 bridge	0.28
	S_1-S_2 bridge	0.37
	S_1-S_3 bridge	1.13
	S_2-S_2 bridge	2.22
	S_2-S_2 bridge	2.26
	S_2-S_3 bridge	2.81
CH_3CH_2SH	S_1 site	0.38
		0.38
		0.16
		0.21
		0.44

The equations used to calculate binding energies are mentioned in the main paper in section 3.2.

Note: A single site can accommodate multiple binding configurations of the same adsorbate, depending on the orientation of the adsorbate.

Gray shades represent similar binding site geometry groupings.

Table S11. Effective Activation Barriers for C-H Dissociation Pathways on 1 ML S-dimer covered (001)-S surface

$\Delta E_{activation,eff} = E_{transition\ state} - E_{most\ stable\ initial\ state}$

$C_2H_6 \rightarrow CH_3CH_2 + H$			
**Initial C_2H_6 Site**	**Final C_2H_5 site**	**Final H site**	**$\Delta E_{activation,eff}$ (eV)**
physiosorbed	dim_1 top	dim_2 top (same dimer)	2.29
physiosorbed	dim_1 top	dim_2 top (same dimer)	2.04
physiosorbed	dim_1 top	dim_2 top (another dimer)	1.94
physiosorbed	dim_1 top	dim_2 top (another dimer)	2.10
physiosorbed	dim_1 top	S_1 top	1.94
physiosorbed	dim_1 top	S_1 top	2.20
physiosorbed	dim_2 top	dim_1 top (same dimer)	2.12
physiosorbed	dim_2 top	dim_1 top (same dimer)	2.29
**physiosorbed**	**dim_2 top**	**dim_1 top (another dimer)**	**1.84**
physiosorbed	dim_2 top	dim_1 top (another dimer)	2.43
physiosorbed	dim_2 top	S_2 top	2.16
physiosorbed	dim_2 top	S_2 top	2.18
**$CH_3CH_2 \rightarrow CH_2CH_2 + H$**			
**Initial C_2H_5 Site**	**Final CH_2CH_2 site**	**Final H site**	**$\Delta E_{activation,eff}$ (eV)**
dim_1 top	physiosorbed	dim_2 top (same dimer)	1.78
dim_1 top	physiosorbed	dim_2 top (same dimer)	1.67
dim_1 top	physiosorbed	dim_1 top (same dimer)	2.31
dim_1 top	physiosorbed	dim_2 top (another dimer)	1.89
dim_1 top	physiosorbed	dim_2 top (another dimer)	1.83
dim_1 top	physiosorbed	dim_2 top (another dimer)	2.20
dim_1 top	physiosorbed	S_1 top	2.00
**dim_2 top**	**physiosorbed**	**dim_1 top (same dimer)**	**1.59**
dim_2 top	physiosorbed	dim_1 top (same dimer)	1.70
dim_2 top	physiosorbed	dim_2 top (same dimer)	2.30
dim_2 top	physiosorbed	dim_1 top (another dimer)	2.82
dim_2 top	physiosorbed	dim_1 top (another dimer)	1.72
dim_2 top	physiosorbed	dim_2 top (another dimer)	2.26
dim_2 top	physiosorbed	dim_2 top (another dimer)	2.68
dim_2 top	physiosorbed	S_1 top	2.07
dim_2 top	physiosorbed	S_2 top	1.80
S_1 top	dim_1-S_1 bridge	dim_2 top	2.39
S_2 top	dim_2-S_2 bridge	dim_1 top	2.43
S_2 top	physiosorbed	dim_2 top	1.83
S_2 top	physiosorbed	S_1 top	1.92
**$CH_3CH_2 \rightarrow CH_3CH + H$**			
**Initial C_2H_5 Site**	**Final CH_3CH site**	**Final H site**	**$\Delta E_{activation,eff}$ (eV)**
**dim_1 top**	**dim_1 top**	**dim_2 top (another dimer)**	**1.50**
dim_1 top	dim_1 top	dim_2 top (another dimer)	1.76

dim_1 top	dim_1 top	dim_2 top (another dimer)	1.84
dim_1 top	dim_1 top	S_1 top	1.77
dim_1 top	dim_1 top	S_1 top	1.80
dim_1 top	dim_1 top	S_2 top	1.83
**dim_2 top**	**dim_2 top**	**dim_1 top (another dimer)**	**1.51**
dim_2 top	dim_2 top	dim_1 top (another dimer)	1.73
dim_2 top	dim_2 top	S_1 top	1.76
dim_2 top	dim_2 top	S_2 top	1.94
S_1 top	dim_1-S_1 bridge	dim_1 top	2.23
S_1 top	dim_1-S_1 bridge	dim_1 top	2.36
S_1 top	dim_1-S_1 bridge	dim_2 top	2.59
S_1 top	dim_1-S_1 bridge	dim_2 top	2.32
S_2 top	S_2-Fe bridge	dim_1 top	2.80
S_2 top	S_2 top	dim_1 top	2.18
S_2 top	S_2-Fe bridge	dim_2 top	2.24
S_2 top	S_2 top	dim_2 top	2.75
S_2 top	S_2-Fe bridge	S_1 top	3.07
S_2 top	dim_2-S_2 bridge	S_1 top	2.41
**$CH_2CH_2 \rightarrow CH_2CH + H$**			
**Initial CH_2CH_2 Site**	**Final CH_2CH site**	**Final H site**	**$\Delta E_{activation,eff}$ (eV)**
dim_1-dim_2	dim_1 top	S_1 top	1.61
dim_1-dim_2	dim_2 top	S_2 top	1.44
physiosorbed	dim_1 top	dim_2 top	1.93
-dim_2	dim_2 top	dim_1 top	2.07
dim_1-S_1	dim_1-dim_2.S_1	dim_1 top	1.61
dim_1-S_1	dim_1- S_1	dim_1 top	1.56
dim_1-S_1	dim_1-dim_2.S_1	dim_2 top	2.10
dim_1-S_1	S_1-dim_1	dim_2 top	2.38
dim_1-S_1	-dim_1	dim_2 top	1.66
dim_1-S_1	S_1-dim_1.dim_2	dim_2 top	1.72
dim_1-S_1	dim_1- S_1	dim_2 top	1.47
dim_1-S_1	dim_1- S_1	S_2 top	2.06
dim_2-S_2	-dim_2	dim_1 top	1.60
**dim_2-S_2**	**dim_2-S_2**	**dim_1 top**	**1.20**
dim_2-S_2	dim_2-S_2	dim_1 top	1.43
dim_2-S_2	dim_2-S_2	dim_2 top	1.35
dim_2-S_2	-dim_2	dim_2 top	1.49
dim_2-S_2	dim_2-S_2	S_1 top	1.62
dim_2-S_2	dim_2-S_2	S_1 top	2.05
dim_2-S_2	dim_2-S_2	S_1 top	1.90
S_1-S_2	S_2-S_1.dim_1	dim_1 top	2.00
S_1-S_2	S_2-S_1.dim_1	dim_1 top	2.15
S_1-S_2	S_2-S_1.dim_1	dim_1 top	3.37
S_1-S_2	S_2-S_1.dim_2	dim_1 top	2.36
S_1-S_2	S_1-S_2	dim_2 top	2.72

S_1-S_2	S_1-S_2	dim$_2$ top	2.64
S_1-S_2	S_1-S_2	dim$_2$ top	2.85
S_1-S_2	S_1-S_2.dim$_2$	S_1 top	3.30
dim$_1$-dim$_2$ (same)	dim$_1$-dim$_2$.S_1	dim$_1$ top	1.82
dim$_1$-dim$_2$ (same)	dim$_2$-dim$_1$.S_2	dim$_2$ top	1.92
dim$_1$-dim$_2$ (same)	dim$_2$-dim$_1$.S_1	dim$_2$ top	2.32
dim$_1$-dim$_2$ (same)	dim$_1$-dim$_2$.S_2	S_2 top	2.38
dim$_1$ button	dim$_1$ button	dim$_2$ top	3.18
dim$_1$ button	dim$_1$ button	dim$_2$ top	3.03
dim$_1$ button	dim$_1$ button	S_1 top	2.92
dim$_2$ button	dim$_2$ button	dim$_1$ top	3.12
dim$_2$ button	dim$_2$ button	dim$_1$ top	3.15
dim$_2$ button	dim$_2$ button	S_1 top	2.87
dim$_2$ button	dim$_2$ button	S_2 top	1.46
**S-S bond dissociation**			
**Initial Configuration of S-dimer unit**			**$\Delta E_{activation,eff}$ (eV)**
No adsorbate			1.43
C_2H_5 on dim$_1$ site			0.99
C_2H_5 on dim$_1$ site			1.11
C_2H_5 on dim$_2$ site			1.11
C_2H_5 on dim$_2$ site			1.20
C_2H_5 on dim$_1$ site + H on dim$_2$ site			0.46
C_2H_5 on dim$_1$ site + H on dim$_2$ site			1.06
C_2H_5 on dim$_2$ site + H on dim$_1$ site			0.47
C_2H_5 on dim$_2$ site + H on dim$_1$ site			1.05

Note: $\Delta E_{activation,eff}$ is estimated with respect to the most stable initial configuration

Gray shades represent the groupings of similar binding site geometries of the initial state and H binding site of the final state.

Table S12. Effective Activation Barriers for C-H Dissociation Pathways on 1 ML S-dimer covered (210)-2S′ surface

$\Delta E_{activation,eff} = E_{transition\ state} - E_{most\ stable\ initial\ state}$

$C_2H_6 \rightarrow CH_3CH_2 + H$			
**Initial C_2H_6 Site**	**Final CH_3CH_2 site**	**Final H site**	**$\Delta E_{activation,eff}$ (eV)**
physiosorbed	dim_1 top	dim_1 top (same dimer)	2.80
**physiosorbed**	**dim_1 top**	**dim_1 top (another dimer)**	**1.83**
physiosorbed	dim_2 top	dim_2 top (same dimer)	2.14
physiosorbed	dim_2 top	dim_2 top (same dimer)	2.22
**physiosorbed**	**dim_1 top**	**S_{1B} top**	**1.91**
physiosorbed	dim_1 top	S_{1B} top	2.06
physiosorbed	dim_1 top	S_{2B} top	2.09
physiosorbed	dim_2 top	S_{1A} top	2.03
physiosorbed	dim_2 top	S_{2B} top	2.26
physiosorbed	S_{1B} top	dim_1 top	2.08
physiosorbed	radical	dim_1 top	2.03
physiosorbed	radical	dim_2 top	2.35
physiosorbed	radical	S_{1B} top	2.17
$CH_3CH_2 \rightarrow CH_2CH_2 + H$			
**Initial C_2H_5 Site**	**Final CH_2CH_2 site**	**Final H site**	**$\Delta E_{activation,eff}$ (eV)**
dim_1 top	physiosorbed	dim_2 top (same dimer)	1.66
dim_1 top	physiosorbed	dim_2 top (same dimer)	1.87
dim_1 top	physiosorbed	dim_2 top (another dimer)	2.04
dim_1 top	physiosorbed	dim_2 top (another dimer)	1.96
dim_1 top	physiosorbed	dim_2 top (another dimer)	1.93
dim_1 top	physiosorbed	S_{1A} top	2.53
dim_1 top	physiosorbed	S_{1B} top	2.07
**dim_2 top**	**physiosorbed**	**dim_1 top (same dimer)**	**1.44**
dim_2 top	physiosorbed	dim_1 top (same dimer)	1.58
dim_2 top	physiosorbed	dim_2 top (another dimer)	2.28
dim_2 top	physiosorbed	dim_2 top (another dimer)	2.38
dim_2 top	physiosorbed	S_{1A} top	1.93
dim_2 top	physiosorbed	S_{1B} top	2.21

$CH_3CH_2 \rightarrow CH_3CH + H$			
**Initial C_2H_5 Site**	**Final CH_3CH site**	**Final H site**	**$\Delta E_{activation,eff}$ (eV)**
dim_1 top	dim_1 top	dim_2 top (same dimer)	2.54
dim_1 top	dim_1 top	dim_2 top (same dimer)	2.64
dim_1 top	dim_1 top	dim_1 top (another dimer)	2.07
dim_1 top	dim_1 top	dim_1 top (another dimer)	2.10
dim_1 top	dim_1 top	S_{1A} top	2.63
dim_1 top	dim_1 top	S_{1B} top	1.96
dim_1 top	dim_1 top	S_{1B} top	2.77
dim_1 top	dim_1 top	S_{2A} top	2.76
dim_1 top	dim_1 top	S_{2B} top	2.23
dim_2 top	dim_2 top	dim_1 top (another dimer)	1.38
dim_2 top	dim_2 top	dim_1 top (another dimer)	1.98
dim_2 top	dim_2 top	dim_1 top (another dimer)	2.21
dim_2 top	dim_2 top	dim_2 top (same dimer)	2.43
dim_2 top	dim_2 top	dim_2 top (same dimer)	3.25
dim_2 top	dim_2 top	dim_2 top (another dimer)	2.01
**dim_2 top**	**dim_2 top**	**S_{1A} top**	**1.14**
dim_2 top	dim_2 top	S_{1A} top	1.15
dim_2 top	dim_2 top	S_{1B} top	1.77
S_{1B} top	S_{1B} top	dim_1 top	2.35
S_{1B} top	S_{1B} top	dim_2 top	2.65
S_{1B} top	S_{1B} top	dim_2 top	2.87
S_{1B} top	S_{1B} top	S_{1A} top	2.38
S_{1B} top	S_{1B} top	S_{2A} top	2.93
$CH_2CH_2 \rightarrow CH_2CH + H$			
**Initial CH_2CH_2 Site**	**Final CH_2CH site**	**Final H site**	**$\Delta E_{activation,eff}$ (eV)**
dim_1-S_{2B}	dim_1-S_{2B}.S_{2A}	dim_1 top	2.22
dim_1-S_{2B}	dim_1-S_{2B}.S_{2A}	dim_2 top	2.47
dim_1-S_{2B}	dim_1-S_{2B}.S_{2A}	dim_2 top	2.34
dim_1-S_{1B}	S_{1B} top	S_{1A} top	2.65
dim_1-S_{1B}	S_{1B} top	S_{1A} top	2.00
dim_1-S_{1B}	dim_1-S_{1B}	S_{1B} top	1.93
dim_1-S_{1B}	S_{1B} top	S_{2A} top	2.42
dim_1-S_{1B}	dim_1-S_{1B}	S_{2A} top	1.98
dim_1-S_{1B}	dim_1-S_{1B}	S_{2B} top	2.14
dim_1-S_{1B}	dim_1-S_{1B}	S_{2B} top	2.40
dim_2-S_{1B}	S_{1B} top	dim_1 top	1.77
dim_2-S_{2A}	S_{2A} top	dim_1 top	2.51
dim_2-S_{2A}	S_{2A} top	S_{1B} top	3.13
dim_2-S_{2A}	-dim_2	S_{1B} top	2.36
dim_2-S_{2B}	dim_2-S_{2B}.S_{1A}	dim_1 top	2.47
dim_2-S_{2B}	dim_2-S_{2B}.S_{1A}	dim_2 top	2.79
**dim_2-S_{2B}**	**-dim_2**	**S_{1A} top**	**1.33**
dim_2-S_{2B}	dim_2-S_{2B}.S_{2A}	S_{1B} top	3.18

S-S bond dissociation	
**Initial Configuration of S-dimer unit**	**$\Delta E_{activation,eff}$ (eV)**
No adsorbate	1.07
C_2H_5 on dim_1 site	0.64
C_2H_5 on dim_1 site	0.77
C_2H_5 on dim_2 site	0.85
C_2H_5 on dim_2 site	1.16
C_2H_5 on dim_1 site + H on dim_2 site	0.61
C_2H_5 on dim_1 site + H on dim_2 site	0.84
C_2H_5 on dim_2 site + H on dim_1 site	0.49
C_2H_5 on dim_2 site + H on dim_1 site	0.78

Note: $\Delta E_{activation,eff}$ is estimated with respect to the most stable initial configuration

Gray shades represent the groupings of similar binding site geometries of the initial state and H binding site of the final state.

Table S13. Effective Activation Barriers for C-H Dissociation Pathways on the Pristine (001)-S surface

$\Delta E_{activation,eff} = E_{transition\ state} - E_{most\ stable\ initial\ state}$

$C_2H_6 \rightarrow CH_3CH_2 + H$			
**Initial C_2H_6 Site**	**Final CH_3CH_2 site**	**Final H site**	**$\Delta E_{activation,eff}$ (eV)**
physiosorbed	Fe top	S top close	2.33
physiosorbed	Fe top	S top close	**2.23**
physiosorbed	Fe top	S top far	2.54
physiosorbed	Fe top	S top far	2.47
physiosorbed	Fe top	Fe top	2.68
physiosorbed	S top	Fe top close	3.22
physiosorbed	S top	Fe top close	3.31
physiosorbed	S top	Fe top far	3.13
physiosorbed	S top	Fe top far	3.06
physiosorbed	S top	S top	2.65
physiosorbed	S top	S top	2.86
**$CH_3CH_2 \rightarrow CH_2CH_2 + H$**			
**Initial CH_3CH_2 Site**	**Final CH_2CH_2 site**	**Final H site**	**$\Delta E_{activation,eff}$ (eV)**
Fe top	physiosorbed	S top close	1.48
Fe top	physiosorbed	S top close	1.60
Fe top	physiosorbed	S top far	1.36
Fe top	physiosorbed	S top far	1.38
Fe top	physiosorbed	Fe top	1.48
Fe top	physiosorbed	Fe top	**1.20**
S top	physiosorbed	Fe top close	1.59
S top	physiosorbed	Fe top close	1.38
S top	Fe-S close	S top close	2.47
S top	physiosorbed	S top close	2.57
S top	Fe-S close	S top far	2.34
**$CH_3CH_2 \rightarrow CH_3CH + H$**			
**Initial CH_3CH_2 Site**	**Final CH_3CH site**	**Final H site**	**$\Delta E_{activation,eff}$ (eV)**
Fe top	Fe top	S top close	2.08
Fe top	Fe top	S top close	2.23
Fe top	Fe top	S top far	1.71
Fe top	Fe top	S top far	**1.68**
Fe top	Fe top	Fe top	2.02
Fe top	Fe top	Fe top	1.97
S top	S top	Fe top close	2.37
S top	S top	Fe top close	2.41
S top	S top	Fe top far	2.12
S top	S top	Fe top far	**1.64**
S top	S top	S top	1.94
S top	S top	S top	1.98

Note: $\Delta E_{activation,eff}$ is estimated with respect to the most stable initial configuration

Table S14. Effective Activation Barriers for C-H Dissociation Pathways on the Pristine (210)-2S′ surface

$\Delta E_{activation,eff} = E_{transition\ state} - E_{most\ stable\ initial\ state}$

$C_2H_6 \rightarrow CH_3CH_2 + H$			
**Initial C_2H_6 Site**	**Final CH_3CH_2 site**	**Final H site**	**$\Delta E_{activation,eff}$ (eV)**
physiosorbed	Fe	S_{1A}	1.97
physiosorbed	Fe	S_{1B}	2.44
physiosorbed	Fe	S_{1B}	2.41
physiosorbed	Fe	S_{2A}	2.41
physiosorbed	Fe	S_{2B}	2.47
physiosorbed	S_{1A}	S_{1A}	1.88
physiosorbed	S_{1A}	S_{1B}	2.41
physiosorbed	S_{1B}	S_{1A}	2.18
physiosorbed	S_{1A}	S_{2A}	1.92
physiosorbed	S_{1A}	S_{2B}	2.01
physiosorbed	S_{1B}	S_{2A}	2.63
physiosorbed	S_{1B}	Fe	2.91
physiosorbed	S_{1B}	Fe	3.24
physiosorbed	S_{2A}	S_{1A}	1.75
**physiosorbed**	**S_{2A}**	**S_{1A}**	**1.66**
physiosorbed	S_{2A}	Fe	2.85
physiosorbed	S_{2B}	Fe	3.22
**physiosorbed**	**gas-phase radical**	**S_{1A}**	**1.69**
physiosorbed	gas-phase radical	S_{1B}	2.35
physiosorbed	gas-phase radical	S_{2B}	2.12
**$CH_3CH_2 \rightarrow CH_2CH_2 + H$**			
**Initial CH_3CH_2 Site**	**Final CH_2CH_2 site**	**Final H site**	**$\Delta E_{activation,eff}$ (eV)**
Fe	physiosorbed	S_{1A}	1.77
Fe	physiosorbed	S_{1A}	1.83
Fe	physiosorbed	S_{1B}	2.04
Fe	physiosorbed	S_{1B}	1.93
Fe	physiosorbed	S_{2A}	1.97
Fe	physiosorbed	S_{2B}	2.05
S_{1A}	physiosorbed	S_{1A}	2.26
S_{1A}	physiosorbed	S_{1B}	2.09
S_{1A}	physiosorbed	S_{1B}	2.67
S_{1B}	physiosorbed	S_{1A}	2.32
S_{1B}	physiosorbed	S_{1A}	2.07
S_{1B}	physiosorbed	S_{1A}	1.75
S_{1A}	physiosorbed	S_{2A}	2.17
S_{1A}	physiosorbed	S_{2A}	1.92
S_{1B}	physiosorbed	S_{2A}	2.02
S_{1B}	physiosorbed	S_{2A}	2.05

S_{1B}	physiosorbed	S_{2B}	1.92
S_{1A}	physiosorbed	Fe	2.56
S_{1B}	physiosorbed	Fe	2.27
S_{2A}	physiosorbed	S_{1A}	1.72
S_{2A}	physiosorbed	S_{1A}	1.74
S_{2A}	physiosorbed	S_{1B}	1.97
S_{2A}	physiosorbed	S_{1B}	2.12
S_{2B}	physiosorbed	S_{1A}	1.87
S_{2B}	physiosorbed	S_{1A}	1.77
S_{2B}	physiosorbed	S_{1B}	**1.65**
S_{2A}	physiosorbed	S_{2B}	1.78
S_{2B}	physiosorbed	S_{2A}	1.85
S_{2A}	physiosorbed	Fe	2.09
**$CH_3CH_2 \rightarrow CH_3CH + H$**			
**Initial CH_3CH_2 Site**	**Final CH_3CH site**	**Final H site**	**$\Delta E_{activation,eff}$ (eV)**
Fe	Fe	S_{1A}	2.29
Fe	Fe	S_{1A}	2.37
Fe	Fe	S_{1B}	2.81
Fe	Fe	S_{1B}	2.41
Fe	Fe	S_{2A}	2.83
S_{1A}	S_{1A}	Fe	1.65
S_{1A}	S_{1A}	S_{1B}	1.76
S_{1A}	S_{1A}	S_{2A}	**1.40**
S_{1A}	S_{1A}	S_{2A}	**1.40**
S_{1A}	S_{1A}	S_{2B}	**1.45**
S_{1A}	S_{1A}	S_{2B}	**1.42**
S_{1B}	S_{1B}	Fe	2.59
S_{1B}	S_{1B}	Fe	3.19
S_{1B}	S_{1B}	S_{1A}	2.00
S_{1B}	S_{1B}	S_{1A}	2.33
S_{1B}	S_{1B}	S_{2A}	2.41
S_{1B}	S_{1B}	S_{2A}	2.55
S_{1B}	S_{1B}	S_{2B}	2.83
S_{2A}	S_{2A}	Fe	2.73
S_{2A}	S_{2A}	S_{1A}	1.69
S_{2A}	S_{2A}-S_{1A} bridge	S_{1A}	1.94
S_{2A}	S_{2A}	S_{1B}	2.53
S_{2A}	S_{2A}	S_{1B}	2.92
S_{2A}	S_{2A}	S_{2B}	2.73
S_{2B}	S_{2A}-S_{1A} bridge	Fe	3.41
S_{2B}	S_{2A}-S_{1A} bridge	S_{1A}	2.00
S_{2B}	S_{2A}-S_{1A} bridge	S_{1A}	2.06
S_{2B}	S_{2B}-S_{2A} bridge	S_{1B}	3.38
S_{2B}	S_{2B}-S_{1A} bridge	S_{2A}	2.70

Note: $\Delta E_{activation,eff}$ is estimated with respect to the most stable initial configuration

Table S15. Effective Activation Barriers for C-H Dissociation Pathways on the Pristine (111)-3S surface

$\Delta E_{activation,eff} = E_{transition\ state} - E_{most\ stable\ initial\ state}$

$C_2H_6 \rightarrow CH_3CH_2 + H$			
**Initial C_2H_6 Site**	**Final CH_3CH_2 site**	**Final H site**	**$\Delta E_{activation,eff}$ (eV)**
physiosorbed	radical	S_1 top	1.78
physiosorbed	radical	S_2 top	2.01
physiosorbed	S_1 top	S_1 top	1.78
physiosorbed	S_1 top	S_1 top	2.41
physiosorbed	S_1 top	S_2 top	2.21
physiosorbed	S_1 top	S_2 top	1.74
physiosorbed	S_1 top	S_3 top	**1.48**
physiosorbed	S_2 top	S_1 top	1.82
physiosorbed	S_2 top	S_1 top	1.65
physiosorbed	S_3 top	S_1 top	1.67
**$CH_3CH_2 \rightarrow CH_2CH_2 + H$**			
**Initial CH_3CH_2 Site**	**Final CH_2CH_2 site**	**Final H site**	**$\Delta E_{activation,eff}$ (eV)**
S_1 top	physiosorbed	S_2 top	1.62
S_1 top	physiosorbed	S_2 top	1.46
S_1 top	physiosorbed	S_2 top	1.67
S_1 top	physiosorbed	S_3 top	**1.25**
S_2 top	physiosorbed	S_1 top	1.60
S_2 top	physiosorbed	S_1 top	1.88
S_2 top	physiosorbed	S_2 top	1.73
S_2 top	physiosorbed	S_3 top	1.36
S_3 top	physiosorbed	S_1 top	1.51
S_3 top	physiosorbed	S_2 top	1.55
**$CH_3CH_2 \rightarrow CH_3CH + H$**			
**Initial CH_3CH_2 Site**	**Final CH_3CH site**	**Final H site**	**$\Delta E_{activation,eff}$ (eV)**
S_1 top	S_1 top	S_1 top	**1.24**
S_1 top	S_1-S_1 bridge	S_1 top	1.34
S_1 top	S_1-S_2 bridge	S_1 top	2.79
S_1 top	S_1 top	S_2 top	1.70
S_1 top	S_1 top	S_2 top	**1.26**
S_1 top	S_1-S_2 bridge	S_3 top	1.32
S_2 top	S_1-S_2 bridge	S_1 top	1.93
S_2 top	S_1-S_2 bridge	S_1 top	2.02
S_2 top	S_1-S_2 bridge	S_1 top	2.35
S_2 top	S_1-S_2 bridge	S_2 top	3.16
S_2 top	S_1-S_2 bridge	S_2 top	2.45
S_2 top	S_1-S_2 bridge	S_3 top	1.65
S_3 top	S_1-S_3 bridge	S_1 top	1.84
S_3 top	S_3 top	S_2 top	1.59

Note: $\Delta E_{activation,eff}$ is estimated with respect to the most stable initial configuration

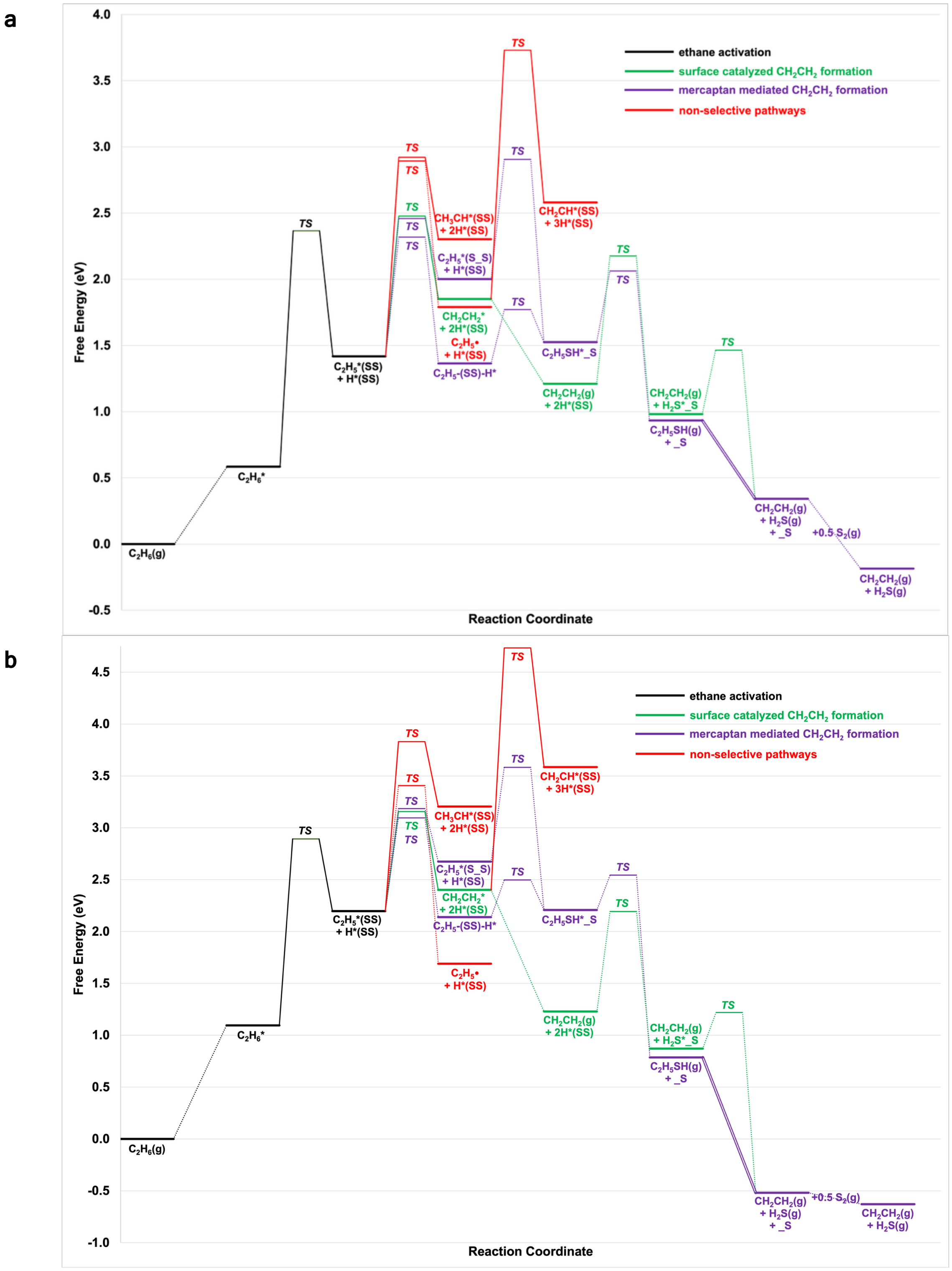

Figure S15. Reaction free energy diagrams of S_2-ODHE on the 1ML S-dimer covered (001)-S facet at temperatures of a) 800 K and b) 1200 K and a partial pressure of 0.01 atm for gas phase S_2.

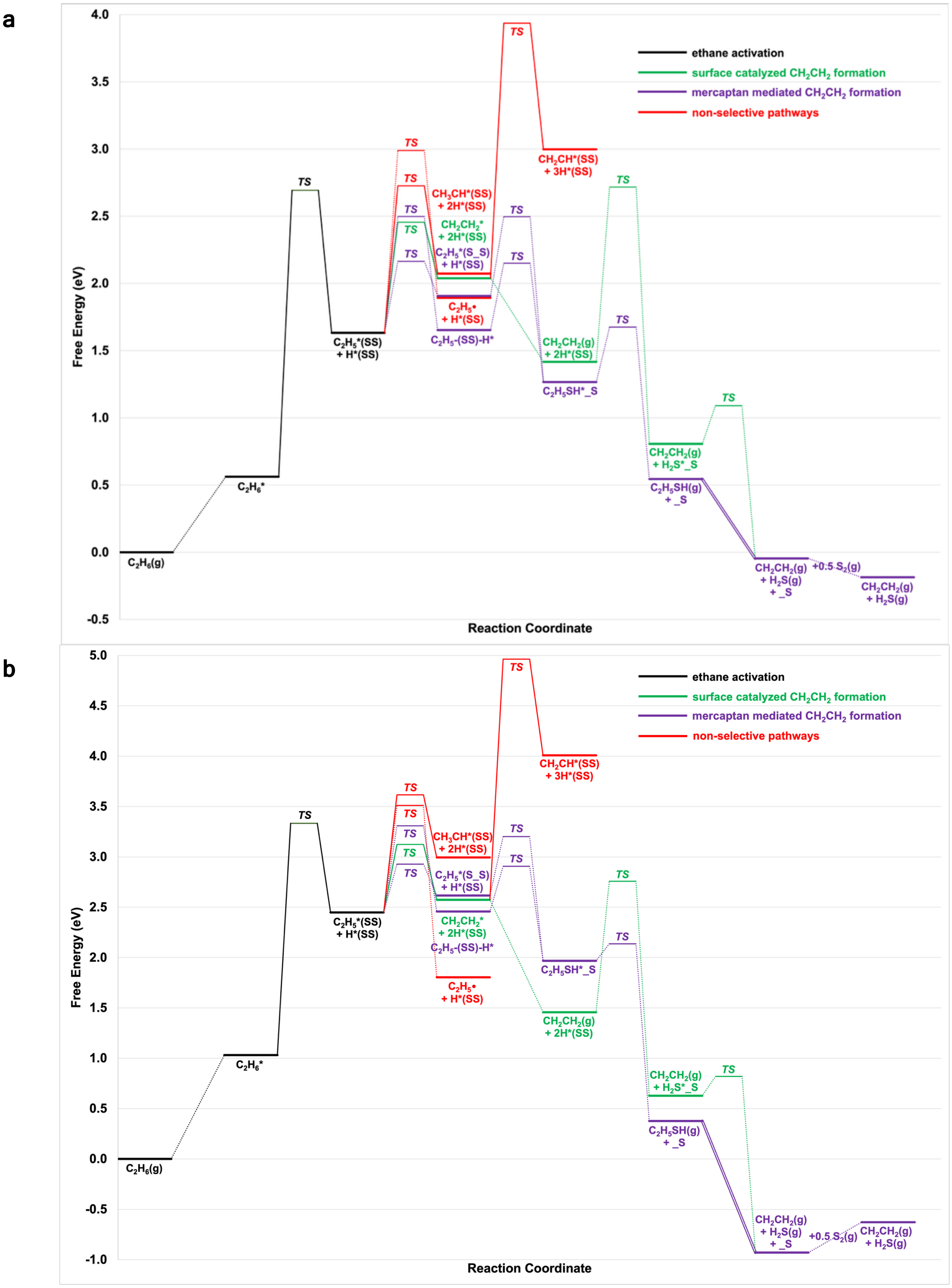

Figure S16. Reaction free energy diagrams of S_2-ODHE on the 1ML S-dimer covered (210)-2S′ facet at temperatures of a) 800 K and b) 1200 K and a partial pressure of 0.01 atm for gas phase S_2.

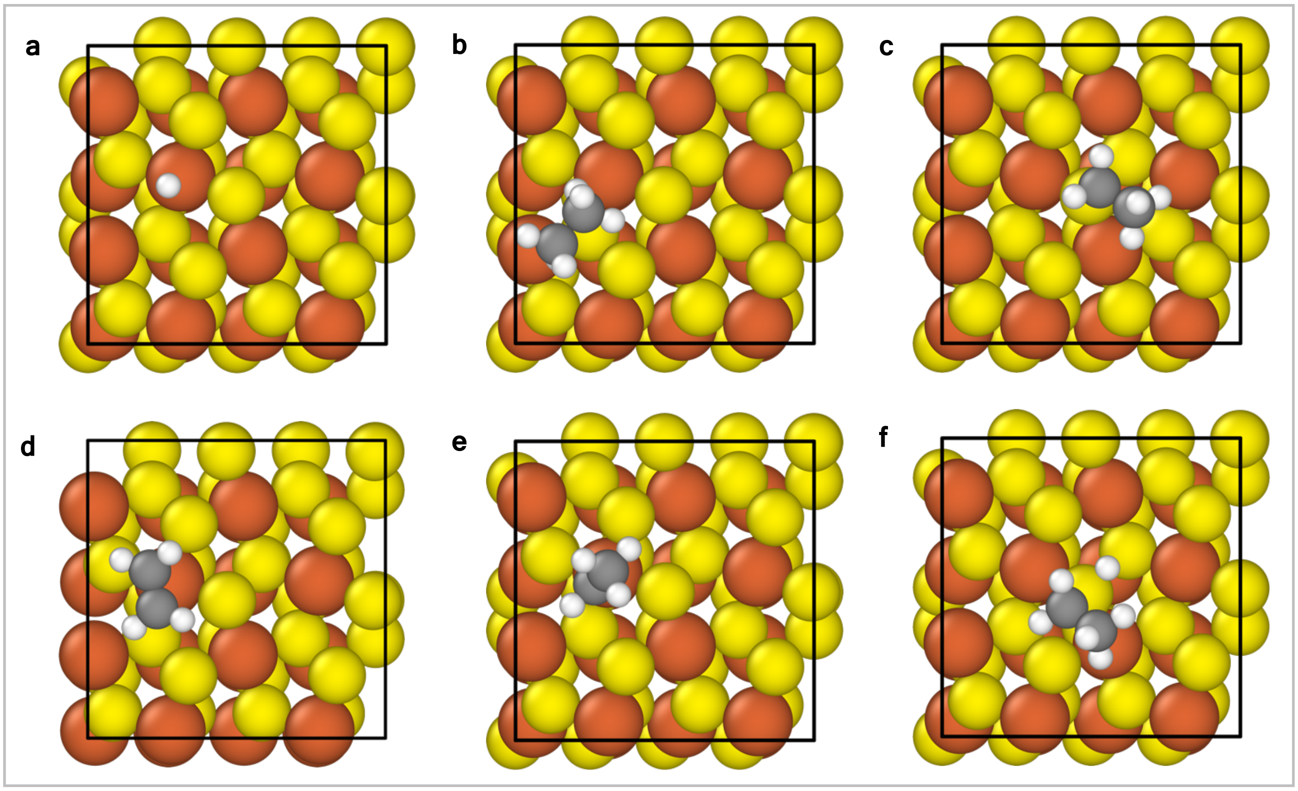

Figure S17. Binding modes of adsorbates *(top views)* at their strongest binding sites on the pristine (001)-S surface with a) H on Fe top site, b) CH_3CH_3 physiosorbed, c) CH_3CH_2 on S top site, d) CH_2CH_2 on Fe site with button configuration, e) CH_3CH on Fe top site, and f) C_2H_5SH involving a surface S-site.

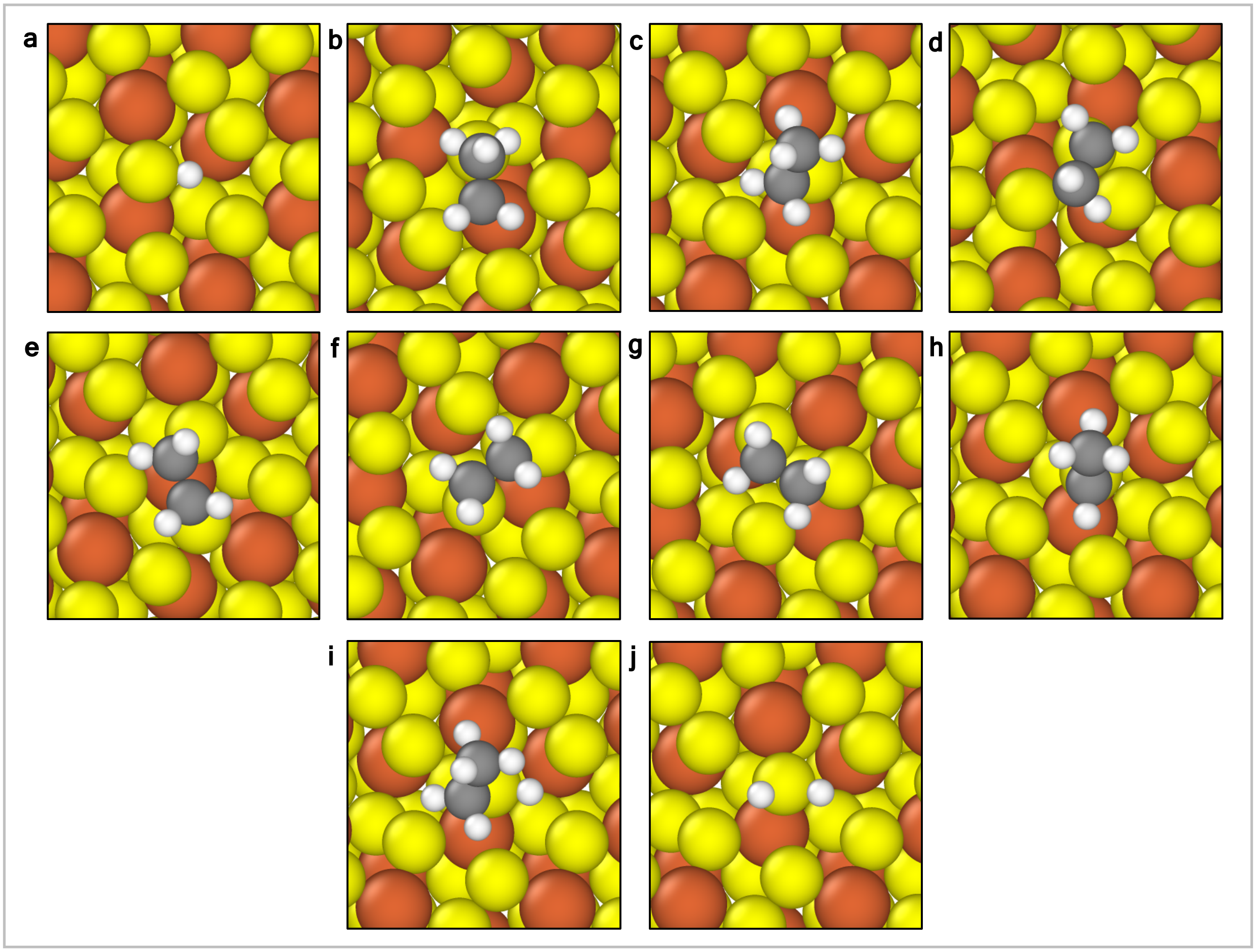

Figure S18. Binding modes of adsorbates *(top views)* at their strongest binding sites on the pristine (210)-2S′ surface with a) H on Fe top site, b) CH_3CH_3 physiosorbed, c) CH_3CH_2 on S_{1A} top site, CH_2CH_2 on d-e) S_{1A}-S_{1B} sites, f) S_{1A}-S_{2A} site, g) S_{1A}-S_{2B} site, h) CH_3CH on S_{1A} site, i) C_2H_5SH involving a surface S_{1A}-site, and j) H_2S on a surface S_{1A}-site.

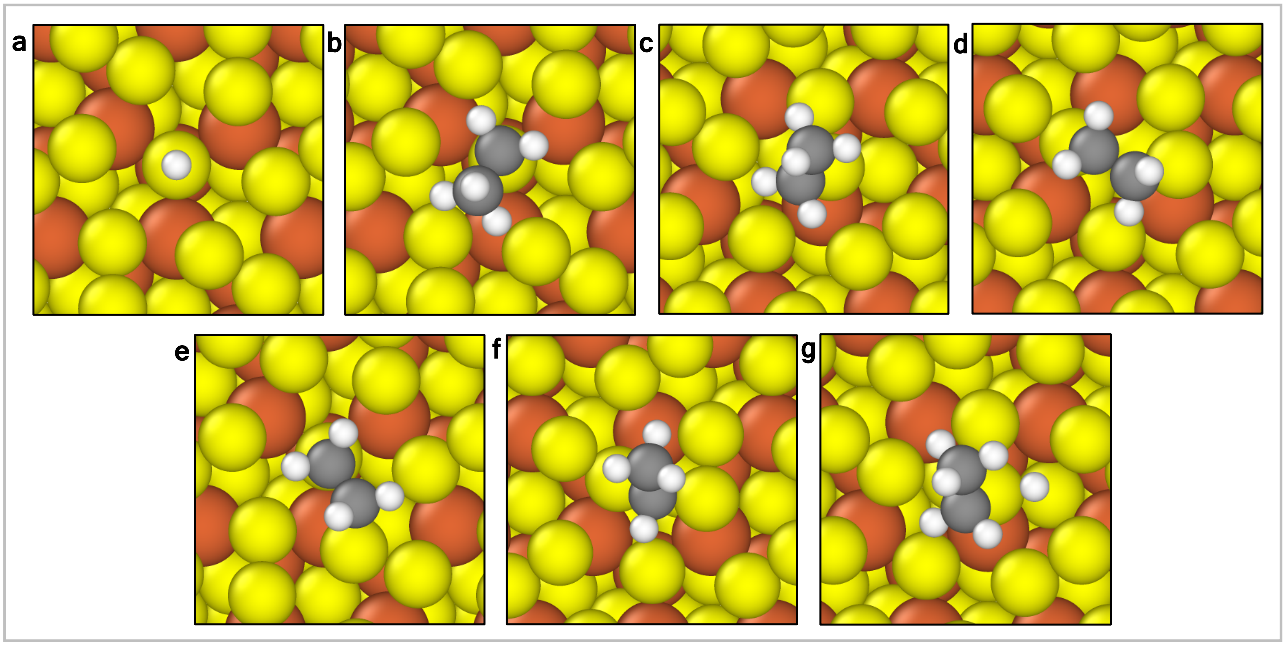

Figure S19. Binding modes of adsorbates *(top views)* at their strongest binding sites on the pristine (111)-3S surface with a) H on S_3 site, b) CH_3CH_3 physiosorbed, c) CH_3CH_2 on S_1 top site, CH_2CH_2 on d) S_1-S_2 site, e) S_1-S_3 site, f) CH_3CH on S_1-S_2 bridge site, and g) C_2H_5SH involving a surface S_1-site.

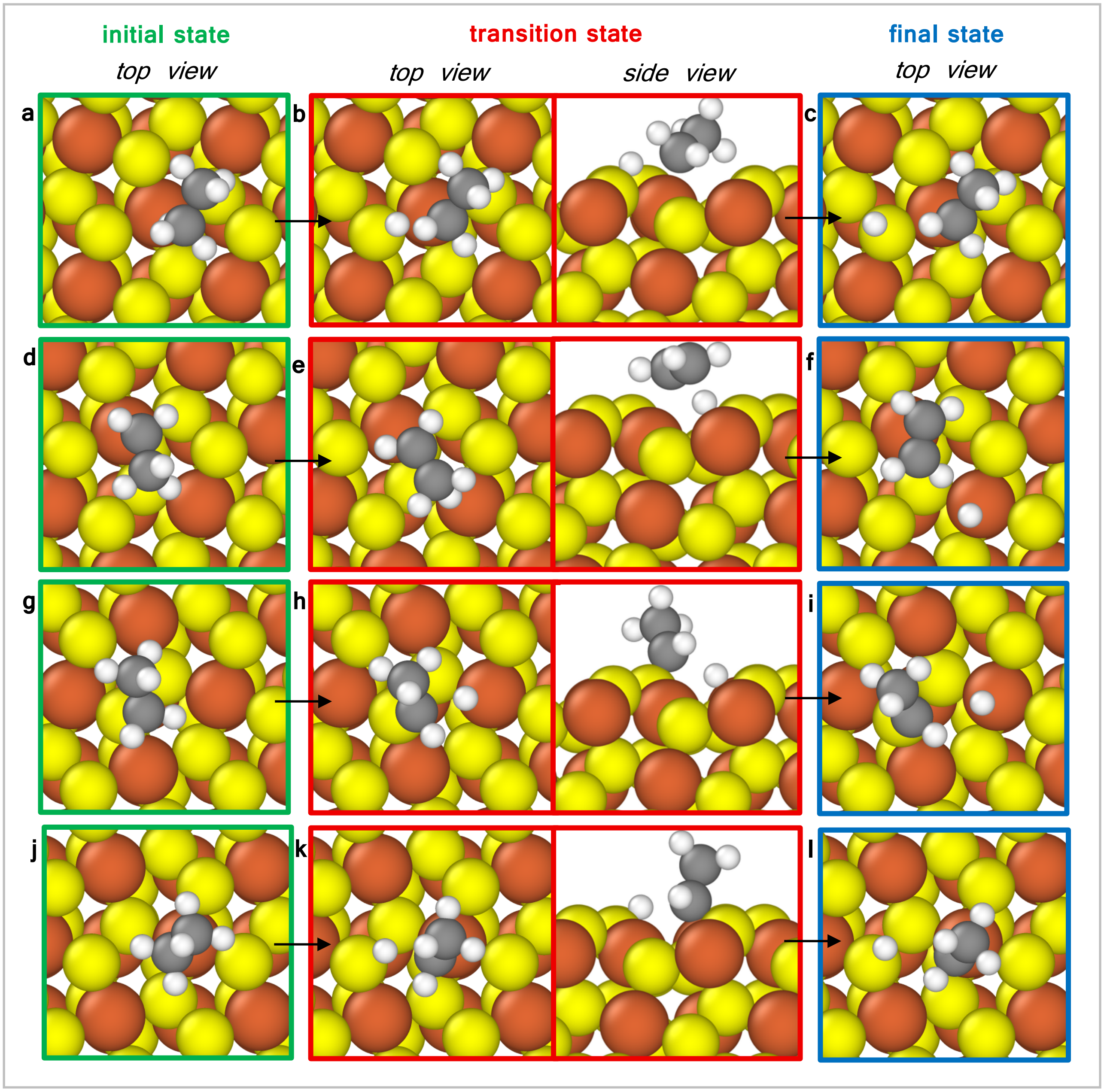

Figure S20. Minimum energy pathway geometries for elementary reactions on the pristine (001)-S surface. For the reaction $CH_3CH_3^* \rightarrow CH_3CH_2^* + H^*$, a) initial *(top view)*, b) transition state *(top and side views)*, and c) final state *(top view)* geometry. For the reaction $CH_3CH_2^* \rightarrow CH_2CH_2^* + H^*$, d) initial *(top view)*, e) transition state *(top and side views)*, and f) final state *(top view)* geometry. For the reaction $CH_3CH_2^* \rightarrow CH_3CH^* + H^*$, there are two minimum energy pathways with similar transition state energy. For the first minimum energy pathway g) initial *(top view)*, h) transition state *(top and side views)*, and i) final state *(top view)* geometry, and for the second minimum energy pathway j) initial *(top view)*, k) transition state *(top and side views)*, and l) final state *(top view)* geometry.

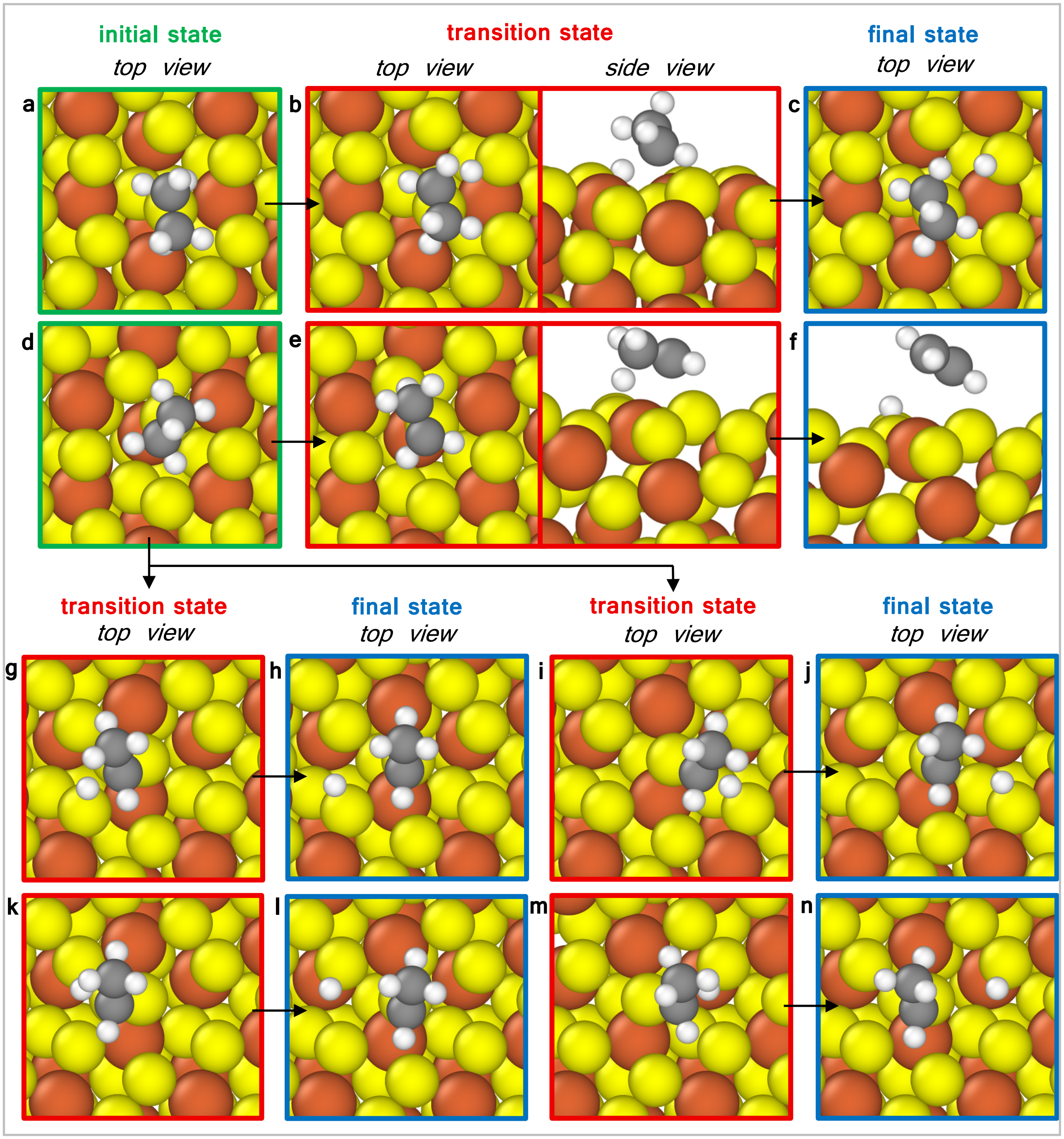

Figure S21. Minimum energy pathway geometries for elementary reactions on the pristine (210)-2S′ surface. For the reaction $CH_3CH_3^* \rightarrow CH_3CH_2^* + H^*$, a) initial *(top view)*, b) transition state *(top and side views)*, and c) final state *(top view)* geometry. For the reaction $CH_3CH_2^* \rightarrow CH_2CH_2^* + H^*$, d) initial *(top view)*, e) transition state *(top and side views)*, and f) final state *(top view)* geometry. For the reaction $CH_3CH_2^* \rightarrow CH_3CH^* + H^*$, there are four minimum energy pathways with similar transition state energy. All of those have the same initial state d. 1st minimum energy pathway g) transition state *(top view)* and h) final state *(top view)*. 2nd minimum energy pathway i) transition state *(top view)* and j) final state *(top view)*. 3rd minimum energy pathway k) transition state *(top view)* and l) final state *(top view)*. 4th minimum energy pathway m) transition state *(top view)* and n) final state *(top view)*.

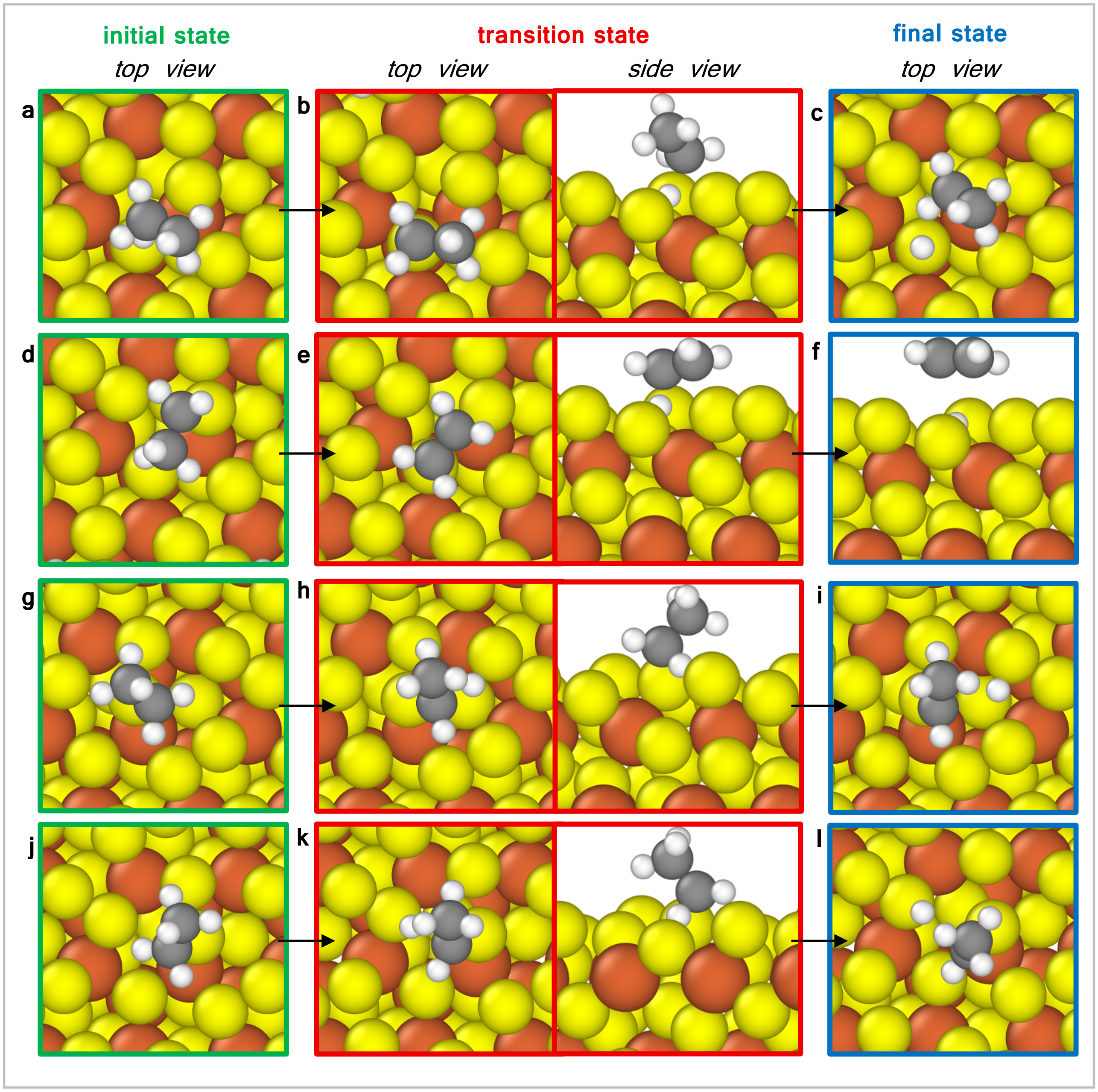

Figure S22. Minimum energy pathway geometries for elementary reactions on the pristine (111)-3S surface. For the reaction $CH_3CH_3* \rightarrow CH_3CH_2* + H*$, a) initial *(top view)*, b) transition state *(top and side views)*, and c) final state *(top view)* geometry. For the reaction $CH_3CH_2* \rightarrow CH_2CH_2* + H*$, d) initial *(top view)*, e) transition state *(top and side views)*, and f) final state *(top view)* geometry. For the reaction $CH_3CH_2* \rightarrow CH_3CH* + H*$, there are two minimum energy pathways with similar transition state energy. For the first minimum energy pathway g) initial *(top view)*, h) transition state *(top and side views)*, and i) final state *(top view)* geometry, and for the second minimum energy pathway j) initial *(top view)*, k) transition state *(top and side views)*, and l) final state *(top view)* geometry.

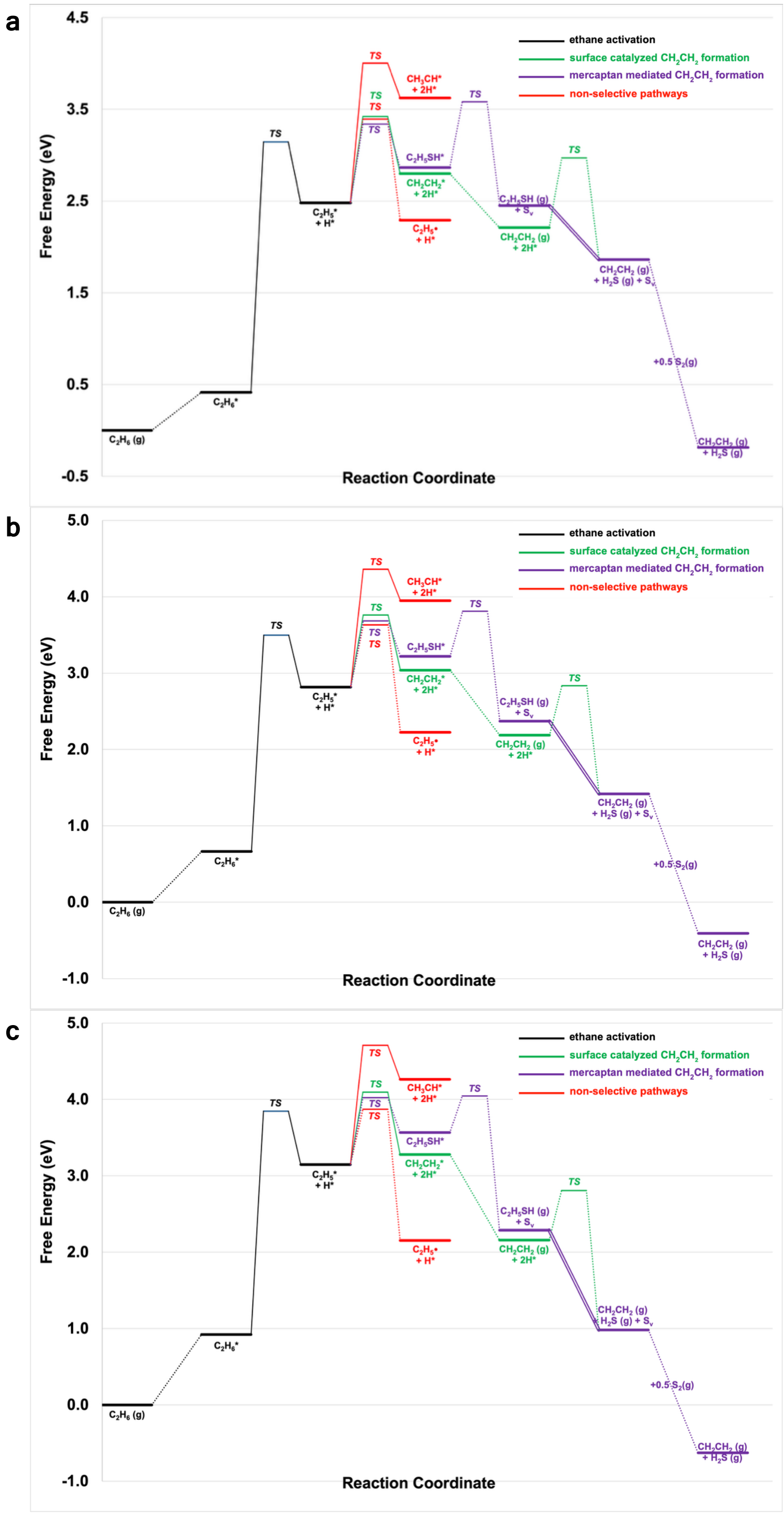

Figure S23. Reaction free energy diagrams of S_2-ODHE on the pristine (001)-S facet at a) 800 K, b) 1000 K, and c) 1200K and at a partial pressure of 0.01 atm for gas phase S_2.

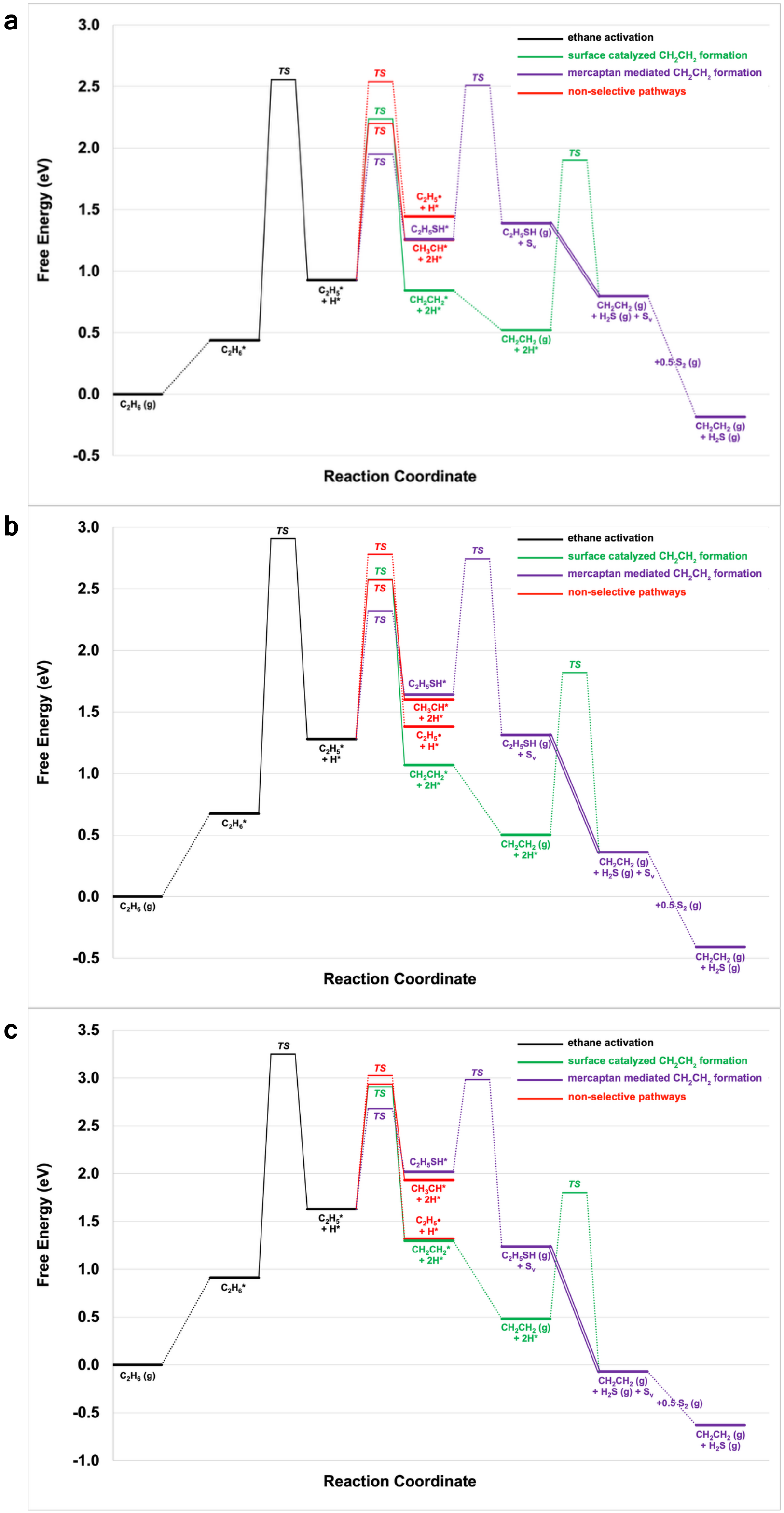

Figure S24. Reaction free energy diagrams of S_2-ODHE on the pristine (210)-2S′ facet at a) 800 K, b) 1000 K, and c) 1200K and at a partial pressure of 0.01 atm for gas phase S_2.

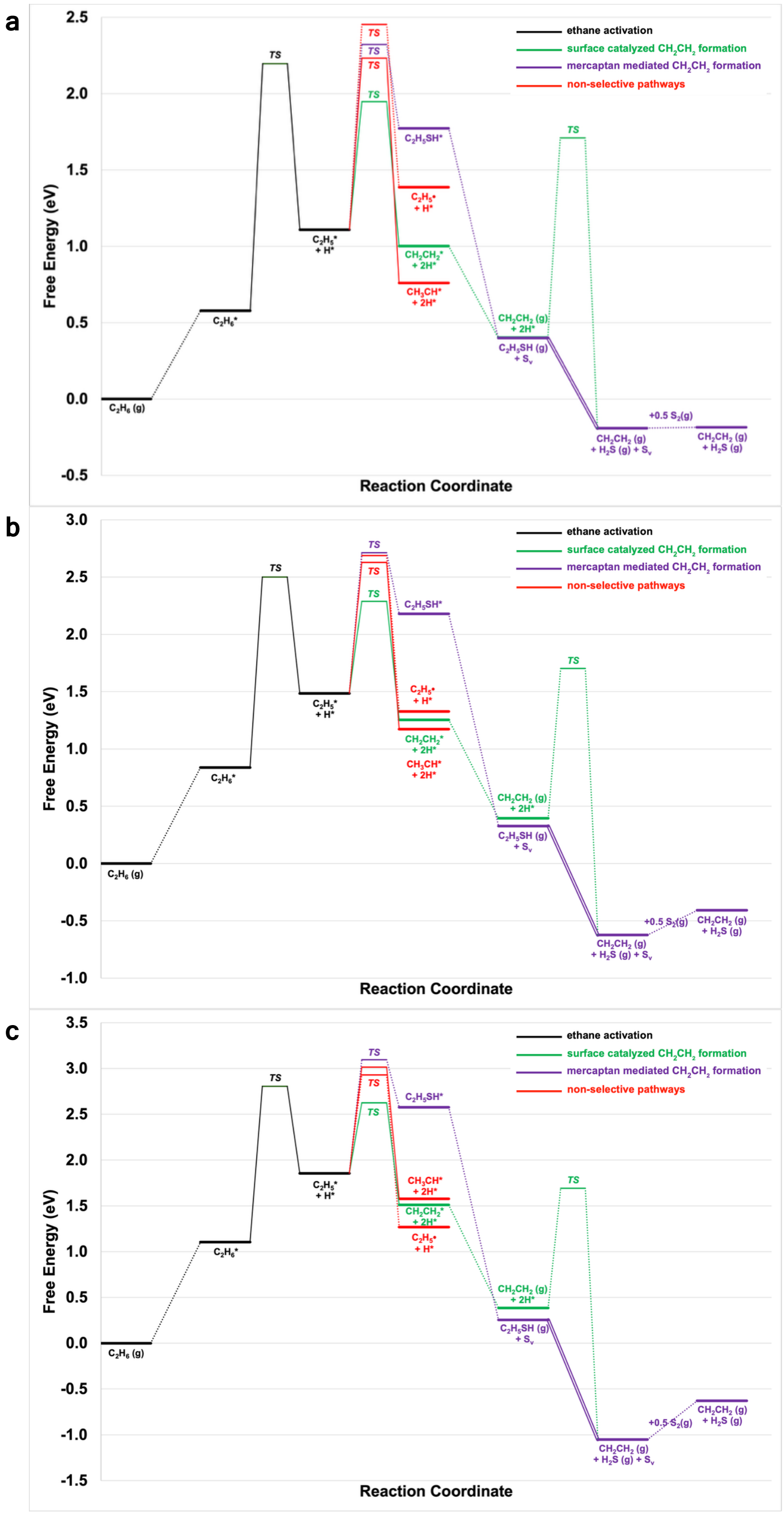

Figure S25. Reaction free energy diagrams of S_2-ODHE on the pristine (111)-3S facet at a) 800 K, b) 1000 K, and c) 1200K and at a partial pressure of 0.01 atm for gas phase S_2.

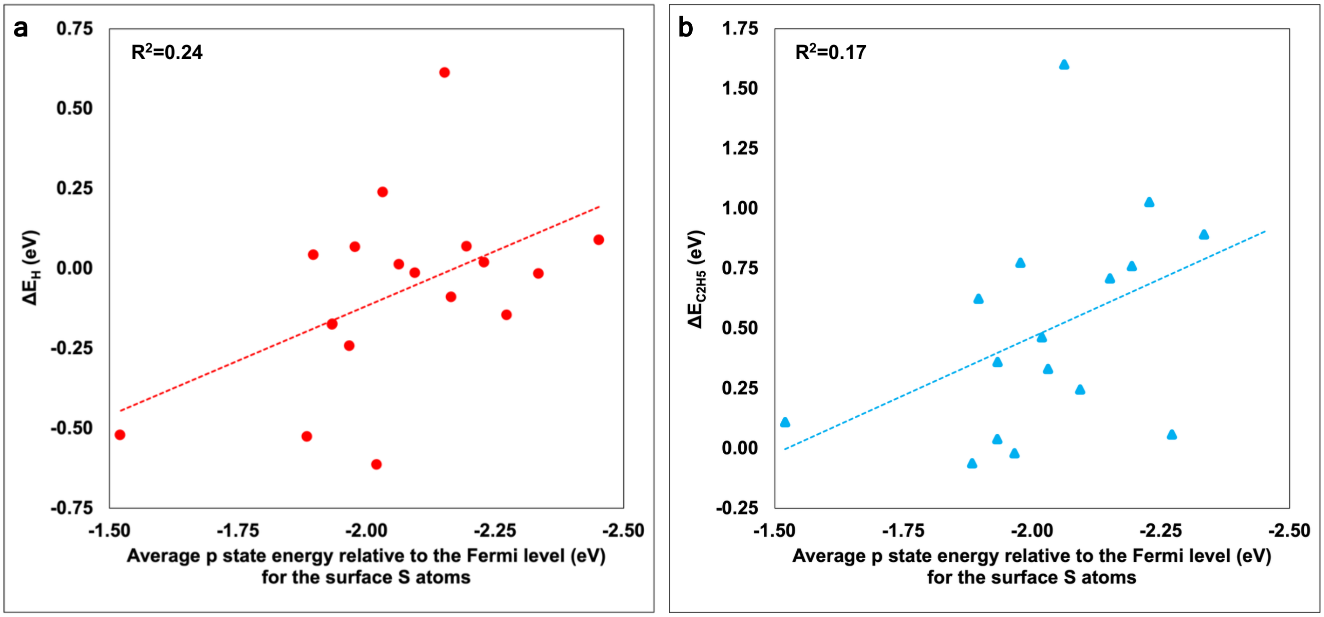

Figure S26. Correlation of a) H and b) C_2H_5 binding energy with average sulfur site valence p-state energy

References

(1) NIST-JANAF Thermochemical Tables - SRD 13, 2013. https://doi.org/10.18434/T42S31.
(2) Reuter, K.; Scheffler, M. Composition, Structure, and Stability of RuO 2 (110) as a Function of Oxygen Pressure. *Phys. Rev. B* **2001**, *65* (3), 035406. https://doi.org/10.1103/PhysRevB.65.035406.
(3) Bollinger, M. V.; Jacobsen, K. W.; Nørskov, J. K. Atomic and Electronic Structure of MoS_2 Nanoparticles. *Phys. Rev. B* **2003**, *67* (8), 085410. https://doi.org/10.1103/PhysRevB.67.085410.
(4) Alfonso, D. R. Computational Investigation of FeS $_2$ Surfaces and Prediction of Effects of Sulfur Environment on Stabilities. *J. Phys. Chem. C* **2010**, *114* (19), 8971–8980. https://doi.org/10.1021/jp100578n.
(5) Sprowl, L. H.; Campbell, C. T.; Árnadóttir, L. Hindered Translator and Hindered Rotor Models for Adsorbates: Partition Functions and Entropies. *J. Phys. Chem. C* **2016**, *120* (18), 9719–9731. https://doi.org/10.1021/acs.jpcc.5b11616.
